# A resilient and connected network of sites to sustain biodiversity under a changing climate

**DOI:** 10.1073/pnas.2204434119

**Published:** 2023-02-06

**Authors:** Mark G. Anderson, Melissa Clark, Arlene P. Olivero, Analie R. Barnett, Kimberly R. Hall, Meredith W. Cornett, Marissa Ahlering, Michael Schindel, Bob Unnasch, Carrie Schloss, D. Richard Cameron

**Affiliations:** ^a^Center for Resilient Conservation Science, The Nature Conservancy, Boston, MA 0211; ^b^Center for Resilient Conservation Science, The Nature Conservancy, Atlanta, GA 30307; ^c^LANDFIRE, The Nature Conservancy, Lansing, MI 48906; ^d^Minnesota Field Office, The Nature Conservancy, Duluth, MN, 55803; ^e^Minnesota Field Office, The Nature Conservancy, Moorhead, MN 56560; ^f^Oregon Field Office, The Nature Conservancy, Portland, OR 97214; ^g^Idaho Field Office, The Nature Conservancy, Hailey, ID 83333; ^h^California Field Office, The Nature Conservancy, San Francisco, CA 94105

**Keywords:** biodiversity, connectivity, resilience, conservation, climate change

## Abstract

Motivated by declines in biodiversity exacerbated by climate change, we identified a network of conservation sites designed to provide resilient habitat for species, while supporting dynamic shifts in ranges and changes in ecosystem composition. Our 12-y study involved 289 scientists in 14 study regions across the conterminous United States (CONUS), and our intent was to support local-, regional-, and national-scale conservation decisions. To ensure that the network represented all species and ecosystems, we stratified CONUS into 68 ecoregions, and, within each, we comprehensively mapped the geophysical settings associated with current ecosystem and species distributions. To identify sites most resilient to climate change, we identified the portion of each geophysical setting with the most topoclimate variability (high landscape diversity) likely to be accessible to dispersers (high local connectedness). These “resilient sites” were overlaid with conservation priority maps from 104 independent assessments to indicate current value in supporting recognized biodiversity. To identify key connectivity areas for sustaining species movement in response to climate change, we codeveloped a fine-scale representation of human modification and ran a circuit-theory-based analysis that emphasized movement potential along geographic climate gradients. Integrating areas with high values for two or more factors, we identified a representative, resilient, and connected network of biodiverse lands covering 35% of CONUS. Because the network connects climatic gradients across 250,000 biodiversity elements and multiple resilient examples of all geophysical settings in every ecoregion, it could form the spatial foundation for targeted land protection and other conservation strategies to sustain a diverse, dynamic, and adaptive world.

## Significance

In response to biodiversity loss, scientists have called for the protection of well-connected systems of protected areas covering 30 to 50% of the planet. However, as climate change drives shifts in species, conservation plans based on current biodiversity patterns will become less effective. We collaboratively develop and map a conservation network for the conterminous United States designed to represent all habitats, while anticipating and facilitating changes in species composition. The network is based on principles of representation, resilience, connectivity, and recognized biodiversity value, with each factor mapped in a way that anticipates climate change. The results are being used to inform land-acquisition and management decisions by The Nature Conservancy, many state and federal agencies, and hundreds of land trusts.

Conservationists in the United States are not winning the battle to sustain biological diversity. Despite broad public support and unprecedented bipartisan agreement on Earth Day 1970, followed by landmark environmental laws, expanded regulatory efforts, and the establishment of hundreds of private conservation organizations, the species and ecosystems that characterize the natural world continue to decline. In North America, the abundance of birds has fallen 29% since 1970 (1); 32% of insect taxa are in decline (2); and 56% of mammalian carnivore and ungulates have shown notable range contractions since 1950 (3). Amphibians have declined an average (avg.) of 33% since 2002 (4). Of the 51,936 species of plants, vertebrates, and macroinvertebrates tracked by NatureServe for the conterminous United States (CONUS), 9% are ranked vulnerable, 12% imperiled, and 1% possibly extinct (5).^*^

Changes in climate are exacerbating species declines, especially for small, isolated populations. As temperature and moisture regimes change, species ranges are shifting with speed and magnitude unprecedented in recent millennia. In the eastern United States, trees have shifted their centers of distribution 10 km north and 11 km west per decade since 1980 (6). Southern bird ranges have shifted northward by an avg. of 23.5 km per decade (7). These changes are on par with global shifts of 10 km north and 11 m upslope per decade across taxa groups (8).

A primary driver of biodiversity decline is habitat loss and degradation resulting from land-use change (9, 10). Land- and water-conservation efforts can reverse these trends when strategically located and enabled by the necessary investments. In North America, billions of dollars spent on wetland restoration and management, combined with more stringent hunting regulations, reversed bird-abundance declines in wetlands (1). Globally, conservation investment from 1996 to 2008 reduced the extinction risk for mammals and birds by a median value of 29% (10). However, the effectiveness of land and water conservation in sustaining biodiversity depends on the representativeness of the conserved area network, the resilience and condition of the sites, and the connectivity between sites to allow for movement and adaptation (11, 12).

To sustain biodiversity and facilitate adaptation of species to a changing climate, the Convention on Biological Diversity (CBD) Target 2 (13) calls for the protection of well-connected and effective systems of protected areas covering at least 30% of the planet. However, as climate change drives changes in species distributions and ecosystem composition, conservation plans based on current biodiversity patterns may become less effective at sustaining species (14). In particular, the current configuration of protected areas may fail to adequately provide access to the diverse climatic conditions needed for species populations to persist amid changing regional climates (12, 15, 16). Accordingly, conservation planners are beginning to focus on conserving sites that represent the earth’s eco-physiographic regions (hereafter “ecoregions”) and the spectrum of geophysical variation and a diversity of connected topographic microclimates (hereafter “topoclimates”) to allow species to adapt in situ or move to newly favorable areas, an approach known as Conserving Nature’s Stage (CNS) (15–19).

Most studies of climate effects on biota use regional-scale climate-projection models combined with species vulnerability assessments to identify areas of relatively high threat or stability at a coarse scale. Here, we take a different approach. By focusing on geophysical diversity that shapes species distributions and fine-scale climate variation directly relevant to species persistence (20, 21), we aimed to identify enduring climate strongholds relevant under many climate scenarios and to map them at scales appropriate for land-conservation decisions.

For species in topographically diverse locations, variability in temperature locally may exceed the degree of warming expected over the next century (22, 23). These areas have the potential to provide species with microclimatic buffering from regional climatic change by allowing local dispersal to more favorable microclimates or providing stepping stones to facilitate longer-distance range shifts (24, 25). Paleoecological records highlight the dynamic nature of species responses to Quaternary climate change, including the role of topography in creating climate refugia (26–28), and suggest that the CNS strategy may be appropriate for many taxa if it is purposefully designed to accommodate species responses to climate change (29).

Species persisted under past climatic changes through in situ refugia combined with range shifts to track suitable climates (30–32). Rapid warming projected for the next century will likely require many species to adapt in a similar way (33–35), and many species’ ranges are already shifting (8). However, high levels of habitat loss and fragmentation due to anthropogenic activities are isolating populations and creating barriers to species movement that were not present during past periods of rapid climate change (29, 36, 37). Thus, conservation actions that maintain or increase connectivity are essential for effective conservation under climate change, as connectivity facilitates movement and gene flow, bolstering adaptive capacity by maintaining genetic diversity (38–40).

To sustain biodiversity, a conservation network must also include sites that support living biotic assemblages reflecting each ecoregion’s geophysical properties, such as dominant habitats, unique communities, and viable examples of rare and specialist species populations. We refer to these as sites with “recognized biodiversity value.” Including them in a conservation network ensures that it is embedded with species and habitats that provide the capacity for adapting to climate change (41, 42). In the United States, state agencies and nongovernment organizations (NGOs) have identified over a thousand areas with recognized biodiversity value through comprehensive ecoregional or state-based assessments specifically targeting viable rare species populations, exemplary natural communities, and intact ecosystems. Integrating the footprint of these sites with spatial information on connected topoclimates and representative geophysical features helps confirm that the sites are collectively distributed across all abiotic “stages” needed to sustain biodiversity into the future.

## Design Approach

To identify a network of lands most likely to sustain biodiversity under a changing climate, we developed CONUS-wide, spatially explicit datasets targeting aspects of a climate-smart conservation network essential to site selection: representation, resilience, connectivity, and recognized biodiversity value (Table 1). Each dataset was reviewed by a steering committee of scientists with expert knowledge of each study region. Map products were evaluated and delivered at a 30-m spatial resolution appropriate for informing local, regional, and national decisions. Subsequently, we assessed the conservation status and ecosystem representation of the network using independent datasets. This study focused exclusively on terrestrial ecosystems and did not assess aquatic or subterranean environments.

**Table 1.** Essential aspects of a network to sustain diversity under climate change

Aspect	Characteristics	Strategy and justification	Metric
Representation	Sites representing an ecologically meaningful portion of every ecoregion distributed across geophysical settings.	Conserve ecological gradients by distributing conservation across ecoregions and among geophysical settings, such as soil and bedrock types, and elevation zones. Ensures capture of the full spectrum of biodiversity.	15 to 40% of each ecoregion distributed across all geophysical settings
Site resilience	Sites with a high diversity of connected topoclimates linked by natural cover and accessible to species.	Conserve representative sites with microclimatic buffering to help species persist longer and turn over more slowly under a changing climate. These sites also serve as natural strongholds for current and future diversity.	Cells with high (>0.5 SD) resilience score relative to ecoregion and geophysical setting
Connectivity and climate flow	Sites positioned along climatic gradients within areas of low human modification (i.e., high climate flow).	Conserve connected corridors and zones of natural cover that follow climatic gradients to allow species to move in response to changing temperature and moisture conditions.	Cells with high (>0.5 SD) climate-flow scores relative to CONUS
Recognized biodiversity value	Sites supporting biotic assemblages characteristic of their geophysical setting (i.e., vegetation types, natural communities, rare and specialist species).	Conserve places that have been recognized for their current biodiversity value to protect species and natural communities where they are already thriving and to provide source areas for dispersing populations.	Cells identified as important for targeted biodiversity in state-based or TNC ecoregional assessments
Network	Co-occurrence of one or more of the above. Resilient sites overlapping with connectivity or biodiversity values.	Integrate the above aspects into an RCN aimed at sustaining biodiversity while facilitating movement and adaptation to change.	Cells in each ecoregion that meet two or three of the above criteria (resilience, flow, biodiversity)

Detail on the quantitative metric representing each characteristic is explained in *Materials and Methods*.

## Results

### Representation and Resilience.

**Fig. 1.**
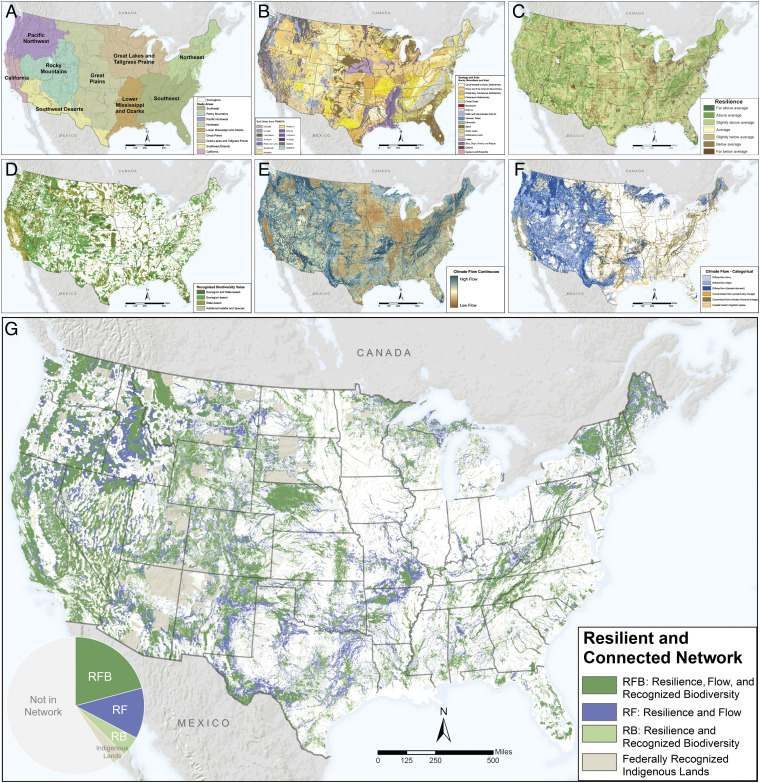
Six component datasets and integrated results. (*A*) Study areas. (*B*) Geophysical settings. (*C*) Site resilience. (*D*) Recognized biodiversity value. (*E*) Climate flow—continuous. (*F*) Climate flow—categorical. (*G*) RCN in three categories: RFB, RF, and RB. Federally recognized indigenous lands are grayed out. For full-sized maps, see *SI Appendix*, Figs. S1–S10.

To represent abiotic variation, we delineated a total of 30 bedrock and surficial geology classes (Fig. 1*B*) and 8 elevation zones distributed across 68 ecoregions. Each ecoregion averaged 8 geology classes (4–13) and included 1 to 8 elevation zones. Differences among geophysical settings (combined elevation and geology classes) corresponded to differences in species and ecosystems associated with soil, bedrock, or elevation zone. Geophysical settings also differed markedly in their number of topoclimates and degree of human modification, the two components of site resilience. This was detected by using resilience scores calculated for each 30-m pixel, transformed to standard normal *z* scores relative to the ecoregional mean. For example, in the Northern Tallgrass Prairie ecoregion, low-elevation calcareous loams had more topographic diversity and less fragmentation than low-elevation fine-grained silts (avg. resilience +1 SD vs. −1.2 SD).

Areas of relatively high site resilience averaged 38% per ecoregion (Fig. 1*C*) and varied from 15% in the heavily developed North Atlantic Coast ecoregion to 86% in the topographically complex Okanagan ecoregion. Among study regions, the Great Lakes and Tallgrass Prairie had the lowest percentage of resilient land (27%), while California (CA; 69%) had the highest.

### Connectivity and Climate Flow.

The continuous and categorical maps of climate flow (Fig. 1 *E* and *F*) indicate that 36% of the country currently had high climate flow (flow > 1 SD), defined as connected areas of low human modification containing local and regional climatic gradients. Flow was unevenly distributed both geographically and locally, with broad diffuse flow common in the West (31%) and concentrated flow found mostly as corridors in the Central and Eastern United States (5%). Zones of high climate flow were biased toward acidic soils, steep slopes, high elevation, and very dry environments, factors that limit human land use. In flat, low-elevation regions fragmented by agriculture or development, flow tended to concentrate in stream corridors following natural landcover in riparian areas. Seventy percent of high-climate-flow areas also scored high for site resilience.

### Recognized Biodiversity Value.

Over 46% of CONUS has been recognized for its value in supporting biodiversity, according to 104 target-based assessments implemented by The Nature Conservancy (TNC) or a state-based entity (Fig. 1*D*). Collectively, TNC sites encompass over 15,000 biodiversity targets consisting of rare species populations (57%) or natural community types (43%) in 300,000+ finer-scale locations. Collectively, TNC portfolio covered 30% of CONUS, although the extent varied widely by ecoregion. State-based conservation priority areas overlapped 9% of TNC portfolios and added an additional 12% to the total area. Regional species-specific assessments, such as Greater Sage-Grouse (*Centrocercus urophasianus*) priority areas, added another 4%. Two-thirds of the area recognized for supporting biodiversity also scored high for site resilience, and this portion contained over 80% of the finer-scale occurrences of biodiversity elements.

### Resilient and Connected Network.

Spatially integrating the components of representation, resilience, connectivity, and recognized biodiversity value resulted in a network of lands covering 34.5% of CONUS (Fig. 1*G* and *SI Appendix*, Figs. S7–S8 and Table S7). The network was designed to represent the full range of geographic, geologic, and local climatic variation in CONUS, but it was unevenly distributed. On average, it included 38% of each ecoregion, but ranged from 82% (Okanagan) to 5% (North Central Tillplain). The Eastern and Central study regions had less area in the network (14 to 30%) than the Great Plains and Western study regions (42 to 60%). Overall, 21% of the network had all three components: high resilience, high climate flow, and recognized biodiversity value (RFB); 12% had high resilience and flow (RF), and 3% had high resilience and biodiversity (RB). In 93% of the ecoregions, the RFB category dominated in area, while in the rest, RF was the most extensive. Land with low site resilience was not included in the network, but could be added if the sites were assessed locally and found to have highly intact or restorable biotic communities. The resilient and connected network (RCN) can be explored in the Resilient Land Mapping Tool (https://maps.tnc.org/resilientland/).

The RCN was slightly biased toward acidic sedimentary bedrocks (4% more than expected) and away from calcareous loamy soils (9% less than expected). Of LANDFIRE’s 402 mapped Biophysical Settings (43), 99.8% had some acreage in the RCN (avg. 51%; *SI Appendix*, Table S9). By distribution, 99% had over 5% of their total area in the RCN, 97% had over 10%, 94% had over 15%, and 77% had over 30%. Only one setting (Mississippi Delta Maritime Forest, 923 acres) was not represented. For settings with low representation (<10%), most of their distribution (avg. 86%) did not meet the site-resilience criteria.

### Securement.

The RCN is 44% secured against conversion by fee, easement, or public ownerships, with 21% protected explicitly for biodiversity (Gap Analysis Project [GAP] 1 and 2) and 23% secured on multiple-use public lands or conservation easements (GAP 3). The secured portion of the RCN covers 15% of CONUS by area, and the unsecured RCN (56%) covers 20% of CONUS. Within the secured RCN, 30% is on land with all three components (RFB), 9% RF, and 5% RB. Two percent of the network occurs on federally recognized tribal lands held by the 344 Sovereign Nations within CONUS. These lands are currently concealed on our web tool at the request of one nation. We respect the sovereignty of Tribal Nations and are committed to undergoing review in collaboration with each Sovereign Nation for their determination as to whether the data for their lands may be shared.

## Discussion

Motivated by declines in biodiversity accelerated by climate change, we identified an RCN of land designed to support the full spectrum of CONUS terrestrial biodiversity both currently and given anticipated changes in species ranges and ecosystem composition. The network contains representative examples of all ecoregions and geophysical environments selected for their connected topoclimates, supports over 250,000 biodiversity elements identified in 104 conservation plans, and is configured to facilitate species population shifts along climatic gradients. Covering 35% of CONUS, the RCN is currently 44% secured against conversion (15% of CONUS). If fully conserved, the network could potentially sustain existing species, while enabling movement, rearrangement, and adaptation to changing climatic conditions. The resulting high-resolution maps are intended to inform local, regional, and national land-protection efforts and investments in sustainable management practices to support biodiversity.

Representation is a critical strategy in biodiversity conservation. Focusing on biodiversity hotspots alone misses suites of species in lower-diversity ecosystems that do not occur elsewhere. We stratified our analysis by ecoregions and geophysical settings to ensure representation of all ecosystems and biota. Overlay of LANDFIRE ecological system data (43), as a validation check on our methods, indicated that the RCN captured the distribution of system types almost completely. Systems underrepresented in acreage included certain prairies, savannahs, and woodlands associated with surficial soils on relatively flat landscapes. As most of these systems have undergone intensive land conversion, the results highlight a bias in the resilience criteria toward less converted and more topographically rich ecosystems (17). Some occurrences of these ecosystems may have high connectivity or extraordinary species diversity that improves their ecosystem resilience, and, as such, they warrant further investigation.

Conservation of landscape features that allow species to persist in situ is an important complement to conservation approaches based on climate projections that characterize patterns in climate-change exposure (25). Regional-scale climate models are invaluable for understanding the direction and magnitude of change, but they have considerable uncertainty (44) and do not account for the local climatic variation that was vital for sustaining refugial populations during past periods of climate change (45, 46). Assessing topoclimatic heterogeneity adds spatial precision to our understanding of how temperature and moisture gradients are distributed across the landscape. A productive next step might be to integrate these two approaches into a model that links geographic variation in climate exposure to the availability of topoclimatic gradients for buffering, as has been done for the western interior United States (47). Because species use topoclimates in multiple ways—to persist for a limited time under deteriorating climatic conditions, to facilitate range shifts, and to persist through a long period of unfavorable climates (24)—our assumption was that the presence of connected topoclimatic variation will help sites remain more diverse over many levels of exposure. The RCN landscapes, and component ecosystems and species populations, could become the focus of measuring climate effects over upcoming decades by monitoring rates of species turnover or compositional change relative to the resilience score.

Current research reinforces the value of connectivity in facilitating adaptation and reversing the impacts of fragmentation (48). The RCN includes a CONUS-wide connectivity assessment at two scales. The fine-scale (30-m) local-connectedness model assesses ecological neighborhoods for topoclimatic options, while the coarser (180-m) wall-to-wall climate-flow model assesses the potential for large-scale directional movement, such as range shifts and migrations. The latter can serve as a blueprint for linking conservation actions across states or organizations to achieve greater conservation impact. For example, TNC has developed a five-state Central Appalachian program aimed at maintaining regional connectivity.

We acknowledge geographic variation in our methods across CONUS. Because our intent was to inform local conservation decisions, we prioritized accuracy within each study region over national consistency. We found that the results were less sensitive to this variation than we expected because subtle differences that mattered locally (e.g., giving less weight to local elevation gradients in the resilience score) tended to affect only a small percentage of the total land area. The greatest geographic difference in our results was in the relative importance of wetlands to site resilience (49). As our study progressed from humid regions to the dry Southwest, we gave increased importance to wetlands as a component of the resilience score (*SI Appendix*, Table S2) and perhaps underestimated their importance in humid landscapes.

The RCN likely encompasses important strongholds for future diversity because of their enduring geophysical characteristics and current biodiversity value, but other contextual and local information will be needed when making conservation protection and management decisions. The extent to which specific species are sustained, and similar levels of biodiversity persist, will vary with the magnitude of climate change and the ecological condition of the sites. Addressing the latter requires information on habitat quality, vegetation composition, disturbance regimes, past and current land uses, and risk of conversion (50). Land managers will need to consider all aspects of a site in making decisions, and proven actions, such as prescribed fire, may be needed to help sustain biodiversity in current or transformed ecosystems.

Our emphasis on enduring land characteristics purposefully implies a long-term perspective. The maps and accompanying reports have allowed us to see the sites and landscapes we seek to protect through a different lens. Rather than solely reacting to near-term drivers of species and habitat loss, this network can help conservationists envision how to allow nature to adjust to a rapidly changing earth, with a goal of sustaining a dynamic, diverse, and adaptive natural world. It is already changing how conservation practitioners work. Encouraged by the Land Trust Alliance (51), hundreds of land-trust and agency staff are using the Resilient Land Mapping Tool to quantify site resilience, connectivity, and other factors to inform land-acquisition decisions. TNC has started focusing conservation on climate corridors and migration space, and TNC’s CA office has developed a property database that uses the RCN to evaluate opportunities for conservation. Several conservation funders now require grant applicants to assess a site’s resilience factors as part of their standard applications. Land managers are also finding creative ways to use the data to inform their work, such as using the landscape-diversity maps to determine areas that favor establishment and long-term regeneration potential for the introduction of heat-tolerant tree species (52).

The area of the RCN suggests that 35% could be the minimum area needed to safeguard CONUS biodiversity if the network is specifically configured to allow for adaptation under a changing climate. Comparable global estimates range from 30 to 50%, depending on how biodiversity targets are defined (53) and the degree to which the objectives also address climate change and ecosystem services (54, 55). Echoing the CBD’s call for a global system of protected areas (13), policymakers in the US Biden–Harris Administration have called for conservation of 30% of the United States, based on the best available science, informed by subject-matter experts, and built on transparent and accessible information (56). To sustain a diverse and dynamic CONUS, it is imperative that an area-based conservation network meet criteria for representation, resilience, connectivity, and recognized biodiversity value. The RCN represents a rigorous spatial blueprint for such a network, meeting the Administration’s science and transparency objectives and available to the public for viewing or download via a web tool. We seek to engage and empower the public in making conservation decisions, because conserving the RCN calls for extensive conservation in regions that are largely privately owned (e.g., Central and Eastern United States). We hope that by inviting users to explore these datasets, test their integrity, and incorporate the information into their own decision-making processes, the RCN will facilitate more collaborative and effective conservation.

## Materials and Methods

### Study Region and Approach.

Our 12-y study covered the entire United States and adjacent parts of Canada, involving 289 scientists representing 50 states and 3 Canadian provinces. To perform the work, we divided the CONUS study area into nine terrestrial and five coastal study regions, each comprising clusters of TNC ecoregions or coastal zones (57). Six study regions were shared with Canada or Mexico, but for this paper, we clipped data at the continental US border to ensure data compatibility (Fig. 1*A*). Here, we present the terrestrial results for the CONUS, amounting to 48 states and 68 full or partial ecoregions, excluding the 2-m sea-level-rise coastal zone.

In all study regions, we applied a similar systematic method; however, each region was allowed to tailor the methods to reflect the local terrain and ecology. Additionally, as this effort unfolded over a decade, our methods evolved to take advantage of new information and improved computational approaches. Thus, the exact techniques for defining geophysical settings, measuring microclimates, determining thresholds, and applying mathematical weightings varied slightly by study region. Ten of the study region assessments were led by one TNC North America team. Project teams for the CA and Pacific Northwest (PNW) study areas were led by staff from their respective TNC state offices and developed innovations and customizations unique to their regions. Relevant variations in methodology are described where applicable and compared in detail in *SI Appendix*, Tables S1, S2, and S4.

Within each study region, we convened a steering committee of TNC scientists from each included state, plus additional conservationists from agencies, academia, and other NGOs. Committee composition varied by geography, but, in aggregate, included contributors from 6 federal agencies, 17 state or provincial agencies, 22 NGOs, 17 universities, 8 Natural Heritage Programs (NHPs), and 48 TNC state offices (*SI Appendix*, Table S10). We used bimonthly virtual meetings to explain the basic methodology, identify relevant datasets, review drafts of results, obtain feedback, iterate, and finalize results. The process took 1 to 3 y per study region, and, for each, we produced a 200- to 300-page report that includes the analytical methods, input datasets, geographic information system (GIS) processing steps, maps of all the components, and a summary for each ecoregion that brings the work together at a decision-relevant scale. The reports were reviewed by steering-committee members and are publicly available for download (*SI Appendix*, *SI Text*).

We began the assessment of each study region with a depiction of geophysical diversity, using data on geology, soils, and elevation to identify abiotic settings that could meaningfully represent key drivers of biodiversity patterns within each ecoregion. Next, we developed maps of site resilience, connectivity, and recognized biodiversity value. Here, we describe the base methods used to develop the foundational data layers that were integrated into a national dataset.

### Representation.

To ensure that the network represented the full spectrum of biodiversity in CONUS, we used TNC ecoregions (57) as a base stratification unit. TNC ecoregions are a spatially continuous collection of 68 physiographic regions that share geographically distinct assemblages of natural communities and species shaped by similar climatic, hydrologic, and geologic factors (Fig. 1*A*). TNC ecoregions were developed in conjunction with the US Department of Agriculture Forest Service and are a modification of Keys et al. ecological units (58). Each ecoregion was further subdivided into geophysical settings based on bedrock geology, soil order or texture, elevation zone, and/or slope class (Fig. 1*B*).

Bedrock geology was mapped by using the US Geological Survey State Geologic Mapping Compilation (59), a seamless, spatial database of 48 state geologic maps at 1:50,000 to 1:1,000,000 scale. All geologic units were crosswalked by major and minor lithologies. Within each study region, we simplified the lithologies into 9 to 14 classes that had a recognizable signature in the current flora and fauna (60).

In areas where bedrock was deeply buried (61) or where soil order was thought to have a stronger influence on diversity, we mapped surficial deposits using SSURGO (62) as our primary data source. We supplemented the dataset with STATSGO2 (63) and POLARIS (64) in areas where SSURGO was not mapped or conflicted with all other soil datasets. Surficial datasets were used at the level of soil order or simplified to three to six texture classes that corresponded to recognizable habitats.

Elevation was taken directly from a 30-m digital elevation model (DEM) (65) and classified into elevation zones corresponding to major changes in dominant vegetation. The boundaries of specific zones differed substantially from the East to the West (*SI Appendix*, Table S1).

In most ecoregions, we confirmed the relevance of each bedrock, soil class, or elevation zone by overlaying independently derived information on vegetation types, natural communities, or rare-species distributions. Our primary point dataset was 300,000+ NHP Element Occurrences, indicating the precise location of natural communities or rare plant and animal populations. These were obtained and used with permission from NatureServe and the 50-state NHP Network (5).

To assess the effectiveness of our representation methods, we overlaid the LANDFIRE Biophysical Settings (BpS undeveloped portion) (43) on our resulting datasets. BpS is a 30-m national dataset representing the ecological systems (vegetation types and natural plant communities) that may have been dominant on the landscape prior to Euro-American settlement.

“Site resilience” is the capacity of a place to maintain species diversity and ecological function as the climate changes. We estimated site resilience as the average of a site’s landscape diversity and local connectedness scores following Anderson et al. (17).

“Landscape diversity” is an estimate of local climatic options (microclimates), defined as the number of topoclimates, density of wetlands, and range of elevations surrounding a given point on the landscape. To evaluate topoclimate variation, we used standard geospatial algorithms in GIS applied to a 30-m DEM to generate terrain-surface indices including the topographic position index, the compound topographic index, the heat-load index, slope, and aspect. These were used to evaluate local variation in slope, aspect, land position, and moisture accumulation and describe the terrain as continuous surfaces of climatic variation. In the PNW and CA, indices were used in focal statistic operations to estimate the range of moisture and temperature settings within a 450-m circular area (*SI Appendix*, Tables S1 and S2). In all other study regions, the topoclimatic variation was partitioned into relatively uniform moisture and temperature combinations equating to specific landform units (e.g., “north-facing cove”) (17), and the variety of landforms around each 30-m cell was calculated by using a 0.41-ha focal window. We accounted for local elevation gradients by calculating the total elevation range within each 0.41-ha circle, regressing the results on the number of landforms, and using the residuals to measure the actual amount of local elevation change relative to the expected amount predicted by the variety of landforms.

Wetland density was measured by using a composite dataset of mapped wetlands derived from the National Wetlands Inventory (66) and the National Land Cover Database (67). Density was calculated as a weighted score of two search areas (0.41 ha and 4.1 ha), giving twice the weight to the smaller area, to account for the size and flatness of large wetland landscapes. In the Southwest Desert study region, an additional boost was also given to moisture-accumulating landforms (wet flat, moist flat, and slope-bottom flat) in the landform variety count by doubling their weight.

We integrated the data surfaces into a single metric of Landscape Diversity with variable weight given to landform variety, wetland density, and/or elevation range, as appropriate in each study region (*SI Appendix*, Table S2).

“Local connectedness” describes the amount and arrangement of connected natural land surrounding each grid cell. The input dataset for this assessment was a spatially continuous grid of “resistance” created by compiling a comprehensive dataset of human modification and assigning weights to each fragmenting feature, reflecting its resistance to movement by species. Human modification components came from standard sources for roads and landcover/land use (67), supplemented with fine-scale data on building footprints, oil and gas wells, solar farms, industrial agriculture, and industrial forestry (*SI Appendix*, Table S3). Relative resistance weights were determined by expert opinion and ranged from 1 (natural land cover) to 20 (high-density development), with values refined following pilot tests and steering-committee review. The map of human modification was converted into a continuous resistance grid by assigning a resistance value to every cell based on its feature type and expert-derived weight.

To measure the degree of local connectedness surrounding each 30-m cell, we applied a resistant kernel analysis to the resistance grid using the algorithm described in Compton et al. (68). The analysis measures the connectivity of each cell to its ecological neighborhood (defined as a 3-km radius) where the cell is viewed as a source of moving organisms or other ecological flows radiating out in all directions. The degree of spread outward from a focal cell is a function of the resistance values of the neighboring cells and their distance from the focal cell. Cell scores were calculated as the modeled area of spread divided by the radius and ranged from 0 to 100.

#### Resilience scores.

Within each ecoregion, scores for landscape diversity and local connectedness were combined to create an estimate of each cell’s site resilience. In most study areas, factor scores were converted to standard normal units (*z* scores) relative to ecoregion and averaged together with equal weight. The PNW and CA regions used quintiles (Qs) instead of *z* scores for binning their resilience scores. The Rocky Mountains and Desert Southwest used a hybrid method to account for the skewed distribution of the connectedness scores.

Ecoregional resilience scores were adjusted to account for the differences in average scores between ecoregions and among geophysical settings. We did this by applying a series of overrides derived from comparing each cell with a different population of cells to ensure that we included the most resilient areas with each stratification method. Our two overrides were: 1) Geophysical Setting, where *z* scores were calculated for each setting/ecoregion combination, and cells that scored > 0.5 SD were applied as an override if the score was higher than the ecoregion score; and 2) Study Region, where *z* scores were calculated for all cells in each study region, and cells that scored > 1 SD for the study region were applied as an override if the score was higher than the ecoregional score and the geophysical setting score.

The overrides had the effect of increasing the resilience scores of cells in ecoregions that were largely intact and topographically complex (i.e., the highest in the study region) or cells that were the best available option for a setting that scored low in its ecoregion (i.e., highest in the setting). Thus, the overrides account for variation in the proportional distribution of resilience scores within ecoregions.

We integrated the ecoregion scores into a single map of site resilience, where cells with scores exceeding the mean by 0.5 SD (or in the top two Qs) were considered “more resilient,” cells near the mean (0) with scores between −0.5 SD and +0.5 SD (middle Q) were considered “average resilience,” and cells below X −0.5 SD (or in the lower two Qs) were considered “less resilient.”

### Connectivity and Climate Flow.

Climate flow was defined as the gradual movement of species in response to climate change across a human-modified landscape. To identify areas potentially important for climate flow, we used an approach that modeled movement potential as a continuous surface based on degree of human modification and geographical climatic gradients. This was done with a minor adaptation of the circuit-theory-based software program Circuitscape (69) to allow for the creation of omnidirectional (“patch-free”) connectivity maps that emphasize variations in the density of current flow corresponding to variations in a resistance surface (*SI Appendix*, Table S4). Our intention was not to map individual species movement, but to identify and locate land areas that 1) had relatively unfragmented natural cover connecting topographically and hydrologically derived climatic gradients, and 2) were positioned in areas likely to intercept a large quantity of potential movement in response to climate change.

To create the climate-flow models for each study region, we started by creating a model of anthropogenic flow using the same resistance grid as described above for local connectedness. Following the methods of Pelletier et al. (70), we executed the circuit model in overlapping regional-scale (3,000 × 3,000 pixel) tiles that collectively covered the entire study region. We nested the tiles within a larger calculation area to avoid noise introduced at the tile edges. Current was passed across the resistance surface of each calculation area in orthogonal directions (N–S, S–N, E–W, and W–E) and summed. The tiles were reassembled and rescaled to the same range of current density by using areas of overlap, creating an omnidirectional map of current density for the study region. The resulting data layer of current density provided a continuous view of current flow across the study region, based solely on anthropogenic features that create resistance. Variations in current flow are driven by interacting and directional resistance factors and reveal both diffuse flow zones (broad regions of high flow) and concentrated corridors (narrow regions where flow converges due to reduced flow in neighboring areas).

To introduce climatic gradients into the anthropogenic flow model, we created a second resistance grid based on slope, gradient, land position, and elevation. This was done by assigning varying resistance values to each unit in the landform model based on its slope and gradient (*SI Appendix*, Table S3). Weights were assigned so that the least resistance was given to units that promoted gradual upslope movement and more resistance to gradients that were very steep or too flat to provide climate relief (change in temperature or moisture). We augmented this resistance grid by intersecting it with the results of a downslope model and giving low resistance to downslope movement that simulated moisture channels and riparian areas. To model downslope movement, we created a continuous 30-m dataset that assigned a relative elevation value to every cell by comparing its elevation to its neighbors within a 3-km neighborhood and identifying areas that were downgradient and lower in elevation than the surrounding landscape.

To model climate flow, we merged the anthropogenic and climate-gradient-resistance grids into one surface, giving equal weight to each factor. Current was passed along the surface orthogonally in all four directions and then combined into one omnidirectional result. To simulate populations moving northward in response to climatic change, the northward directional grid was given 50% more weight than other directions.

In CA and in the PNW, climate flow was modeled by using Omniscape (71), a recent moving-window implementation of Circuitscape that produces comparable results to the wall-to-wall approach. Connectivity important for climate adaptation was identified by using Omniscape to connect natural lands with a current climate to natural lands with climate projected to be similar in the future along topographically diverse routes. Discrete climate linkages were identified as areas with more flow than expected in the absence of both barriers and climate considerations.

To facilitate interpretation, the continuous climate-flow results from both methods were converted to a categorical map using a neighborhood analysis (Fig. 1 *E* and *F*). We calculated the mean and variation of flow in a 1,000-acre focal area around every cell and defined groups as follows: Corridors (X > 1, VAR > 0.5), Diffuse (X > −0.5, VAR < −0.5), Constrained (X = −0.5 to 1, VAR > 0.5), and Blocked (X < −0.5, VAR = any).

### Recognized Biodiversity Value.

Our intention in this section was to evaluate sites for evidence that they support rare or specialized species and characteristic communities. Subsequently, this allowed us to identify areas where these representative targets were provided with microclimatic buffering to help them persist under a changing regional climate and ensure that the connected network contained species and habitats to serve as source material for movement and rearrangement. To identify areas recognized for their biodiversity value, we compiled data from 104 published assessments representing two main sources: TNC Ecoregional Assessments and State Wildlife Action Plans (*SI Appendix*, Table S5).

TNC’s portfolio of biodiversity sites was developed through 68 separate ecoregional assessments completed between 1999 and 2010 (72). Each assessment developed a comprehensive list of targets for the ecoregion consisting of rare or specialist species and characteristic natural communities (*SI Appendix*, Table S8). NHP element occurrences were used to identify multiple locations of each target, and viability criteria based on the size, condition, and landscape context were used to rank each occurrence. Representation goals were set based on the distribution, rarity, and spatial pattern of the targets. A spatial portfolio of sites was identified that aimed to meet representation goals for all viable target occurrences. This resulted in a set of sites that, if conserved, would collectively protect the biological diversity of the ecoregion. The portfolio datasets have a high degree of consistency, as the target lists and sites were reviewed by experts in the various taxa groups.

We also incorporated sites recognized in 38 state-based wildlife and habitat assessments. The majority were Conservation Opportunity Areas (COAs) mapped as part of each state’s Wildlife Action Plan, but where COAs had not been mapped, we also compiled comparable state-wide assessments if they were spatially explicit and had clearly defined terrestrial targets. The state datasets varied widely in terms of conservation targets and expansiveness. Some COAs were identical to the TNC portfolio, while others incorporated different priorities identified through multiple assessments with their own objectives and methods. Most COAs focused on nongame animal species and habitats.

We supplemented the TNC and COA data in some study regions with additional sources of biodiversity data, such as more recent NHP element occurrences and state or regional assessments of priority species (*SI Appendix*, Table S5). Land protected for biodiversity (GAP 1 or 2) (73) was added as examples of intact natural habitat, as TNC portfolios in Western ecoregions had been developed to complement, not include, existing conservation lands.

### Secured Lands.

To evaluate the conservation status of the network and its components, we compiled a national dataset of lands permanently secured against conversion to development (hereafter “secured lands”). These were compiled from national, regional, and state data sources and included private fee-owned land, conservation easements, and permanent conservation restrictions, as well as state and federally owned public lands (*SI Appendix*, Table S6 and Fig. S9). The datasets were combined into a nonoverlapping single layer, and the results were reviewed in each geography by the steering committee. Where missing, the Gap Status (73) was assigned by knowledgeable experts based on owner intent and management regime. Land intended for nature conservation with all natural processes was assigned to GAP 1 or, if subject to management, assigned to GAP 2. Land intended for multiple uses was assigned to GAP 3.

### Integration.

Our integration methods were designed to connect the most resilient portion of each ecoregion and geophysical setting into a network that maximized its co-occurrence with land recognized for biodiversity and climate flow. Statistically, the area of the network was defined by the area of above-average resilience in each ecoregion (avg. 38%) decreased to the subset that co-occurred with medium (med)-high flow or had recognized biodiversity value. To perform the integration, we first classified the continuous site resilience (R) and climate flow (F) datasets into statistical classes (high, med, or low) and the site-based biodiversity (B) dataset into binary classes (in or out). The continuous datasets were in standard normal distributions with a mean of zero and SD of one (*z* scores), and we set the following thresholds: high, >0.5 SD; medium, −0.5 to 0.5 SD; and low, <X −0.5 SD (the Q equivalent for CA and PNW were: high, Q1 and Q2; medium, Q3; and low, Q4 and Q5). We intersected the three datasets to create a single grid of all categorical combinations and intersected that with GAP 1 to 3 secured land (Table 2). We considered the area representing the three-way combination of high resilience, high flow, and biodiversity value (RFB) to form the base network. To emphasize climate adaptation, we also included in the network the two-way combination of high resilience and high flow (RF), as these necessary linkages may have unassessed biodiversity value now or in the future. The two-way combination of high resilience and biodiversity value (RB) was also investigated for inclusion, but inspection revealed that these areas were often small and isolated. Hence, we added them to the network (along with isolated R) only if they were on land already secured against conversion. Our final RCN thus included four combinations (Table 2). Unsecured RB and other combinations might warrant inclusion in the network, but, by definition, the excluded areas are isolated or have average to low resilience; thus, field investigation into the condition and context of these areas should be used to determine if they should be added to the RCN.

**Table 2.** Integration of the thematic components into the RCN

Code	Resilience (R) High: >0.5 SD Med: −0.5 to 0.5 Low: <−0.5 SD	Flow (F) High: >0.5 SD) Med: −0.5 to 0.5) Low: <−0.5 SD)	Biodiversity (B) Yes: TNC or SWAP No: Not in any plan	Securement (s) s/Yes: GAP 1 to 3 u/No: Unsecured	Network
RFB	High	Med-high	Yes	N/A	Yes
RF	High	Med-high	No	N/A	Yes
sRB	High	Low	Yes	Yes	Yes
sR	High	Low	No	Yes	Yes
uRB	High	Low	Yes	No	
uR	High	Low	No	No	
FB	Med or low	Med or high	Yes	N/A	
F*	Med or low	Med or high (VH > 1 SD)*	No	N/A	
B*	Med or Low	Low	Yes (irreplaceable)*	N/A	

The code identifies the components that co-occur at the cell—for example, RFB indicates that the cell has high resilience, high climate flow, and is within an area recognized for biodiversity value. The top four rows form the basic framework of a network design, while other rows indicate components that could be added to the network after local investigation. N/A, not applicable; VH, very high.

^*^Sites of extraordinary value were included in CA.

## Supplementary Material

Appendix 01 (PDF)

## Data Availability

All data included in the article and/or *SI Appendix* may be downloaded at the Center for Resilient Conservation Science (https://crcs.tnc.org/) (74).

## Footnotes

^*^Element occurrence data from NatureServe was provided through agreements developed for each study region.
