# Supplementary material for: A resilient and connected network of sites to sustain biodiversity under a changing climate
Source: Proc Natl Acad Sci U S A. 2023 Feb 6;120(7):e2204434119. doi: 10.1073/pnas.2204434119 (PMC9963216; doi:10.1073/pnas.2204434119)
Supplement: Supplementary file 1 — Appendix 01 (PDF) [file pnas.2204434119.sapp.pdf]

PNAS

Supplementary Information for

A Resilient and Connected Network of Sites to Sustain Biodiversity under a Changing Climate

Mark G. Anderson manderson@tnc.org , Melissa Clark melissa_clark@TNC.ORG , Arlene P. Olivero arlene_olivero@TNC.ORG , Analie R. Barnett abarnett@TNC.ORG , Kimberly R. Hall kimberly.hall@TNC.ORG , Meredith W. Cornett mcornett@TNC.ORG , Marissa Ahlering mahlering@TNC.ORG , Michael Schindel mschindel@TNC.ORG, Bob Unnasch bob@sound-science.org, Carrie Schloss cschloss@TNC.ORG , Richard Cameron dcameron@TNC.ORG

Corresponding Author: Mark Anderson manderson@tnc.org

This PDF file includes:

Supplementary Text

Figures S1 to S10

Tables S1 to S10

Supplementary References

Table of Contents:

Supplementary Text

General Data and Mapping Resources…………………………………………………………...p.4

List of Terrestrial Resilience Study Region Reports…..……………………………………….. p. 5-6

List of Coastal Resilience Study Region Reports …………………………………………… p. 7

Figures

Figure 1. Ecoregions and Study Regions… ………………………………………………….p. 8

Figure 2. Geologic Settings………………………………………………………………......p. 9

Figure 3. Resilience……………………………………………………………………….…….. p. 10

Figure 4. Climate Flow Continuous……………………………………………………….……... p. 11

Figure 5. Climate Flow Categorical ……………………..…………………………………….…….. p. 12

Figure 6. Recognized Biodiversity Value………………………………………………………... p. 13

Figure 7. Resilient and Connected Network (detailed classes)…………………………….…. p. 14

Figure 8. Resilient and Connected Network (summary classes)……………….………………p. 15

Figure 9. Secured Areas……………………………………………………….…………….. p. 16

Figure 10a. Extent and Securement of the Resilient and Connected Network by Ecoregion p. 17

Figure 10b. List of Ecoregions by number and name as shown on Figure 10a……………… p. 18

Tables

Table S1. Landscape diversity components and geophysical setting sources by region … p. 19

Table S2. Regional Methods for Component Weighting and Integration into

Landscape Diversity Scores……………………………………………………………………p. 20-21

Table S3. Resistance grid variables and data sources…………………………………………p. 22

Table S4. Connectivity modeling approaches, parameters, and classification………………p. 23

Table S5. Recognized biodiversity data sources……………………………………………….

A. Summary component table……………………………………..………….. p. 24

B. TNC Ecoregional plan sources…………………………………….…………..p. 24-33

C. State sources……………………………………………………….………….. p. 34-43

D. Additional sources…………………………………………………….………… .p. 44

Table S6. Secured lands data sources..…………………………………………………………p. 45

Table S7. Distribution and securement of the network by ecoregion………………………… p. 46-49

Table S8. Example of TNC Ecoregional Plan Conservation Target List: Central Appalachian Ecoregion

Vertebrates………………………………………………………………………….…p. 50

Plants…………………………………………………………………………………..p. 51-53

Invertebrates…………………………………………………………………………..p. 54-58

Terrestrial and Palustrine Communities…………………………………………………p. 59-64

Table S9. LANDFIRE Biophysical System Representation within the

Resilient and Connected Network (RCN)……...………………………………………..p. 65-78

Table S10. List of scientists and conservationists who contributed time or participated on a steering committee to create the Resilient and Connected Network for the Continental U.S…………p.79-84

Supplementary References ………………………………………………………………………p.85

General Data and Mapping Resources

Data and Report Access: http://nature.org/climateresilience
Download the GIS data (national, state clips), access mapping tools, and link to the supporting documents and regional reports.

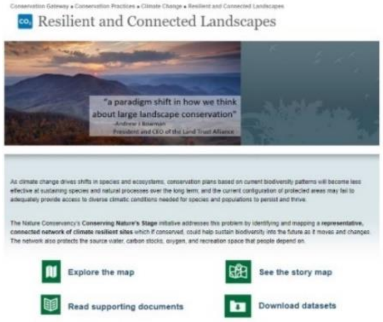

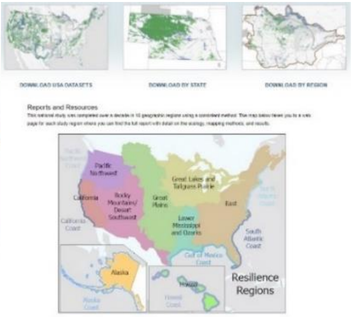

Resilient Land Mapping Tool: http://maps.tnc.org/resilientland/
Visualize the core resilience, connectivity, and network data, explore the component data, access the carbon estimate maps, and generate reports for user defined polygon areas.

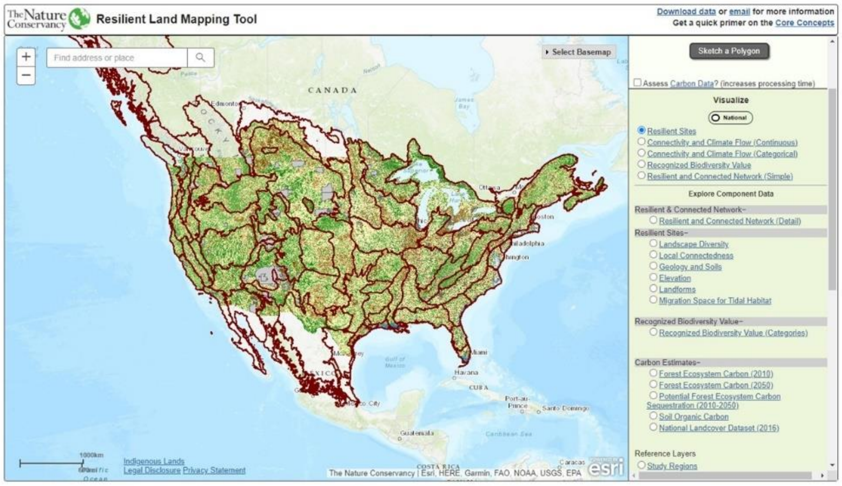

Authoritative Data and AGOL Services: https://crcs.tnc.org/pages/data
Access each of the core data layers published for online mapping.

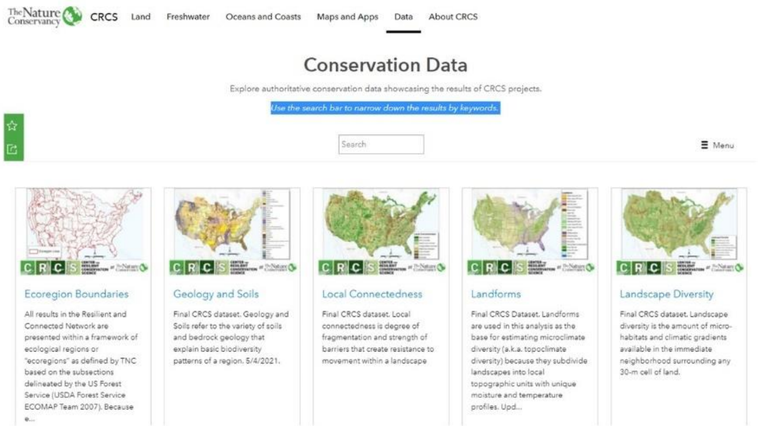

List of Terrestrial Resilience Study Region Reports

TNC's Resilient and Connected Network (RCN) is a proposed conservation network of representative climate-resilient sites designed to sustain biodiversity and ecological functions into the future under a changing climate. The network was identified and mapped over a 10-year period by Nature Conservancy scientists using public data available at the state and national scale, and an inclusive process that involved over 250 scientists from agencies, academia and NGOs across the US.

All region's resilience reports can be accessed from the Interactive Reports and Resources Map found on http://nature.org/climateresilience or from the individual websites and direct links below.

Eastern U.S. Region: Website
https://www.conservationgateway.org/ConservationByGeography/NorthAmerica/UnitedStates/edc/reportsdata/terrestrial/resilience/Pages/default.aspx

Resilient and Connected Landscapes: Report
Anderson, M.G., Barnett, A., Clark, M., Prince, J., Olivero Sheldon, A. and Vickery B. 2016. Resilient and Connected Landscapes for Terrestrial Conservation. The Nature Conservancy, Eastern Conservation Science, Eastern Regional Office. Boston, MA.
http://easterndivision.s3.amazonaws.com/Resilient_and_Connected_Landscapes_For_Terrestial_Conservation.pdf

Resilient Sites: Report
Anderson, M.G., A. Barnett, M. Clark, C. Ferree, A. Olivero Sheldon, J. Prince. 2016. Resilient Sites for Terrestrial Conservation in Eastern North America. The Nature Conservancy, Eastern Conservation Science.
http://easterndivision.s3.amazonaws.com/Resilient_Sites_for_Terrestrial_Conservation.pdf

Central U.S Region: Website
https://www.conservationgateway.org/ConservationByGeography/NorthAmerica/UnitedStates/centralUS/ConnectedLandscapes/Pages/default.aspx

Resilient and Connected Landscapes Central U.S.: Report
Anderson, M.G., M. Clark, A. Olivero Sheldon, K. Hall, J. Platt, J. Prince, M. Ahlering, and M. Cornett. 2018a. Resilient and Connected Landscapes for Terrestrial Conservation in the Central U.S.. The Nature Conservancy, Eastern Conservation Science, Eastern Regional Office. Boston, MA. https://tnc.app.box.com/s/50r22xaf7aaxhs5tx4ep1hsuc24pfg0c

Resilient Sites Great Plains Region: Report
Anderson, M.G., M.A. Ahlering, M. M. Clark, K.R. Hall, A. Olivero Sheldon, J. Platt and J. Prince. 2018b. Resilient Sites for Terrestrial Conservation in the Great Plains. The Nature Conservancy, Eastern Conservation Science and North America Region. Boston MA.
https://easterndivision.s3.amazonaws.com/GP_Resilience/Great_Plains_Resilience.pdf

Resilient Sites Great Lakes and Tallgrass Prairie Region: Report
Anderson, M.G., M. M. Clark, M.W. Cornett,,K.R. Hall, A. Olivero Sheldon, J. Prince. 2018c. Resilient Sites for Terrestrial Conservation in the Great Lakes and Tallgrass Prairie. The Nature Conservancy, Eastern Conservation Science and North America Region.
https://easterndivision.s3.amazonaws.com/Terrestrial/Great_Lakes_Resilience/Great_Lakes_and_Tallgrass_Prairie_Resilience_05_11_18.pdf

Lower Mississippi-Ozark Region: Website
https://www.conservationgateway.org/ConservationByGeography/NorthAmerica/UnitedStates/centralUS/lowerMississippiOzarks/Pages/default.aspx

Resilient Sites and Connected Landscapes: Report
Anderson, M.G., M. M. Clark, A. Olivero, and J. Prince. 2019b. Resilient Sites and Connected Landscapes for Terrestrial Conservation in the Lower Mississippi-Ozark Region. The Nature Conservancy, Eastern Conservation Science. https://tnc.app.box.com/file/612375896177

Rocky Mountains and Desert Southwest Region: Website
https://www.conservationgateway.org/ConservationByGeography/NorthAmerica/UnitedStates/westernUS/Pages/Rocky-Mountains-Desert-Southwest-Resilient-and-Connected-Lands.aspx

Resilient Sites and Connected Landscapes: Report
Anderson, M.G., M. M. Clark, A. Olivero, and J. Prince. 2019a. Resilient Sites and Connected Landscapes for Terrestrial Conservation in the Rocky Mountain and Southwest Desert Region. The Nature Conservancy, Eastern Conservation Science.
https://tnc.app.box.com/file/622379073752

Pacific Northwest: Website: http://nature.org/resilienceNW

Pacific Northwest Resilient Terrestrial Landscapes: Report
Buttrick S, Popper K, Schindel M, McRae BH, Unnasch B, Jones A, Platt J. 2015. Conserving Nature's Stage: Identifying Resilient Terrestrial Landscapes in the Pacific Northwest. The Nature Conservancy, Portland, Oregon. 104 pp.

Pacific Northwest Connectivity for Resilient Terrestrial Landscapes: Paper
McRae BH, Popper K, Jones A, Schindel M, Buttrick S, Hall K, Unnasch RS, Platt JT. 2016a. Conserving Nature's Stage: Mapping Omnidirectional Connectivity for Resilient Terrestrial Landscapes in the Pacific Northwest. The Nature Conservancy, Portland Oregon. 47 pp.

California:

California: Resilient and Connected Network Report
Schloss, C.A and Cameron, D.R. 2021. The Resilient Connected Network in California Technical Documentation. The Nature Conservancy, California.
https://tnc.box.com/s/a4bd75ogf5dlah6jds66lxgbnyd29ho9

California: Connectivity Papers
Schloss, C.A., Cameron, D.R., McRae, B.H., Theobald, D.M. and Jones, A. 2022. "No-regrets" pathways for navigating climate change: planning for connectivity with land use, topography, and climate. Ecological Applications, 32(1), p.e02468.
https://esajournals.onlinelibrary.wiley.com/doi/full/10.1002/eap.2468
Webmap: https://omniscape.codefornature.org/#/analysis-tour.

Cameron, D. R., Schloss, C. A., Theobald, D. M., & Morrison, S. A. 2022. A framework to select strategies for conserving and restoring habitat connectivity in complex landscapes. Conservation Science and Practice, 4(6), e12698.
https://conbio.onlinelibrary.wiley.com/doi/full/10.1111/csp2.12698

List of Coastal Resilience Study Region Reports

Coastal areas provide critical habitat for wildlife and are home to more than 40 percent of the U.S. population, but coastal sites vary widely in their ability to accommodate rising sea levels. Scientists from The Nature Conservancy evaluated over 12,000 coastal sites along the Atlantic Seaboard and Gulf of Mexico for their capacity to sustain biodiversity and natural services under increasing inundation from sea level rise. Each site received a resilience "score" based on the likelihood that its coastal habitats can and will migrate to adjacent lowlands. A coastal site was considered more resilient if it had more options for adapting to, or accommodating risk, and more vulnerable if it had less options. Resilience scores for coastal sites were integrated into the national Resilient and Connected Network. Please see these individual reports for details on the coastal resilience analyses and results.

Coastal Northeast and Mid-Atlantic U.S.

Anderson, M.G. and Barnett, A. 2017. Resilient Coastal Sites for Conservation in the Northeast and Mid-Atlantic U.S.. The Nature Conservancy, Eastern Conservation Science. Boston, MA
View the interactive map, download the data, and read the report at:
https://www.nature.org/resilientcoasts

Coastal South Atlantic U.S.

Anderson, M.G. and Barnett, A. 2019. Resilient Coastal Sites for Conservation in the South Atlantic US. The Nature Conservancy, Eastern Conservation Science.

View the interactive map, download the data, and read the report at: https://www.nature.ly/SEcoast

Gulf of Mexico U.S

Anderson, M.G. and Barnett, A. 2019. Resilient Coastal Sites for Conservation in the Gulf of Mexico US. The Nature Conservancy, Eastern Conservation Science.

View the interactive map, download the data, and read the report at: https://www.nature.ly/Gulfcoast

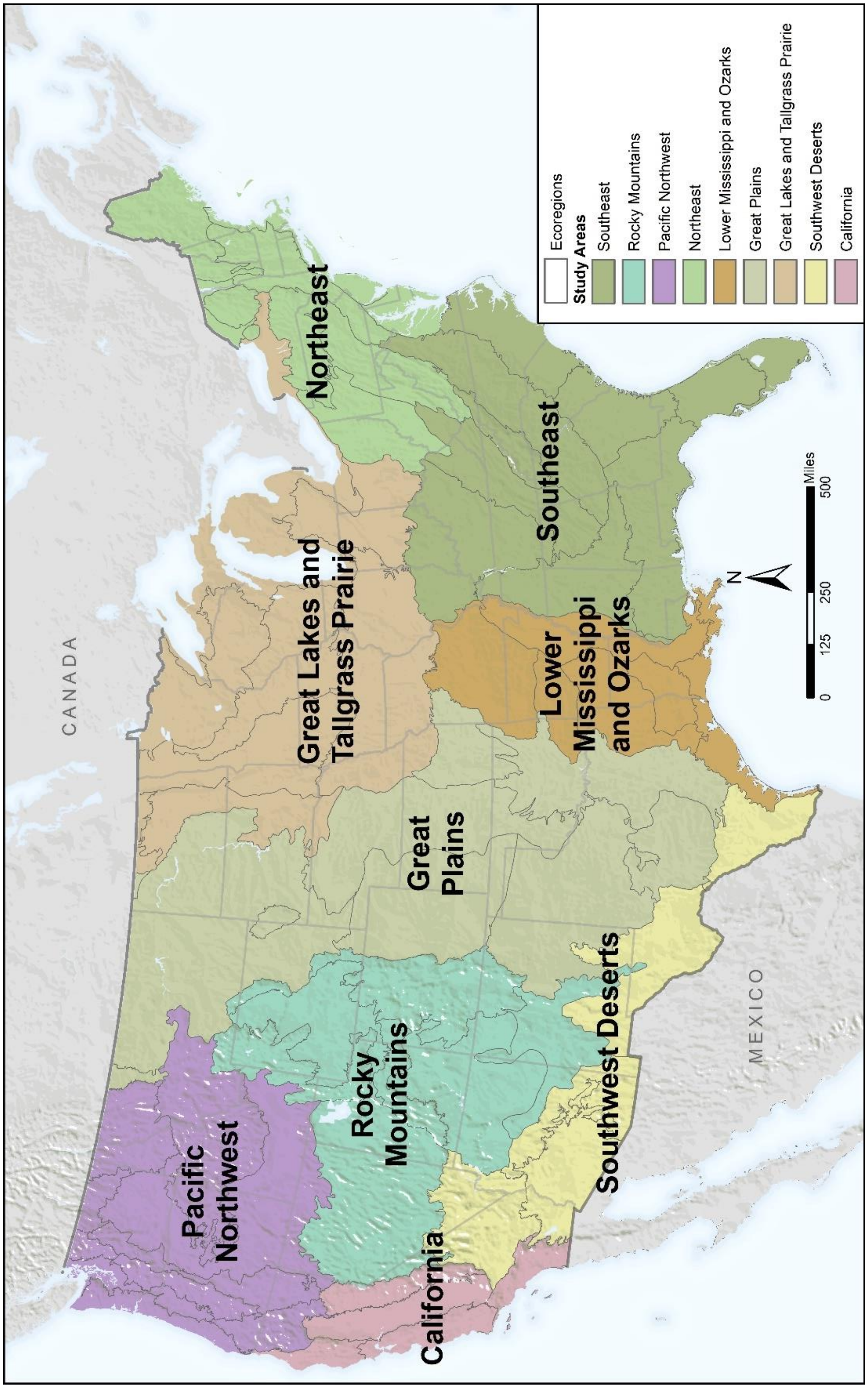

Figure 1. Ecoregions and Study regions

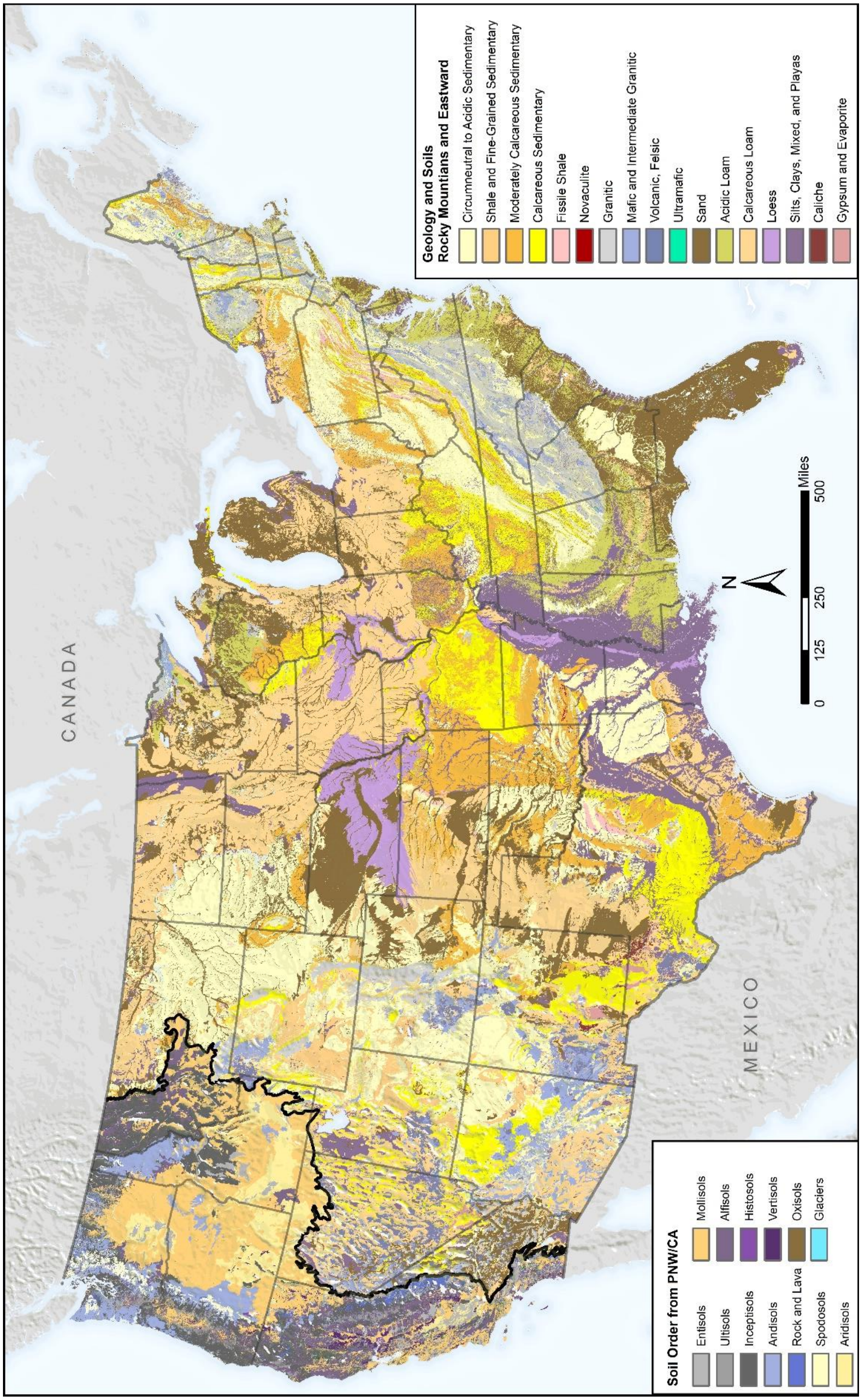

Figure 2. Geologic Settings

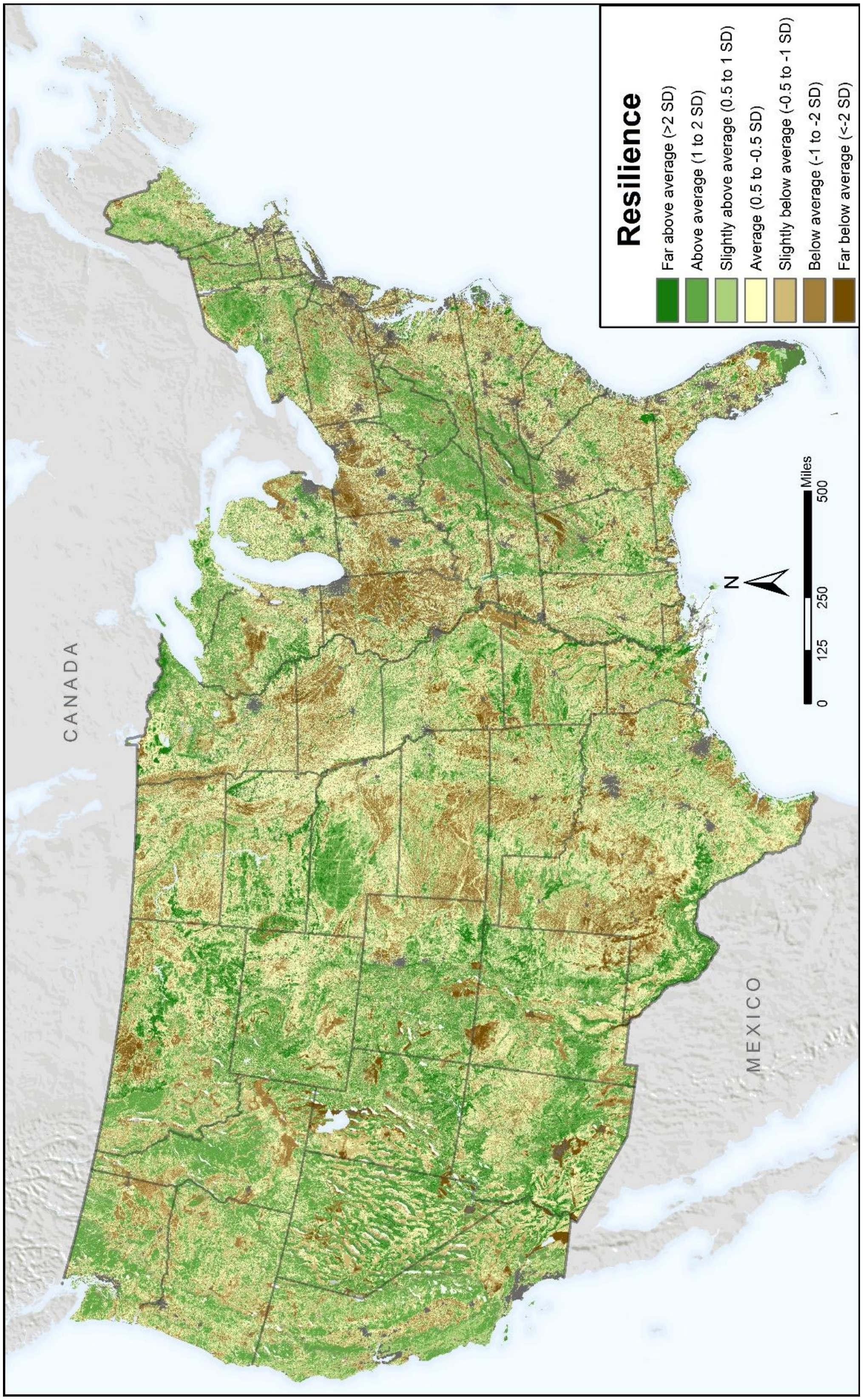

Figure 3. Resilience

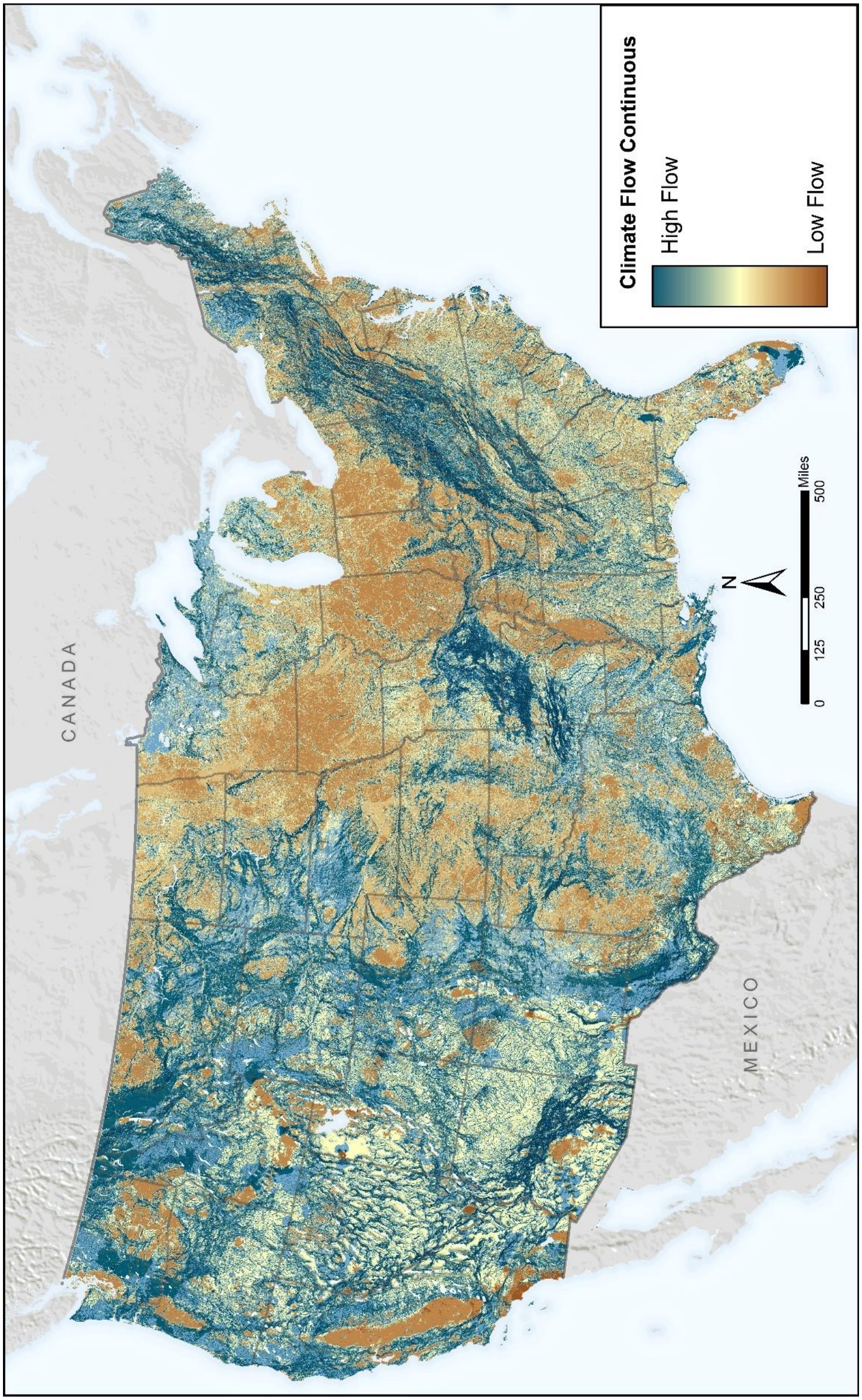

Figure 4. Climate Flow Continuous

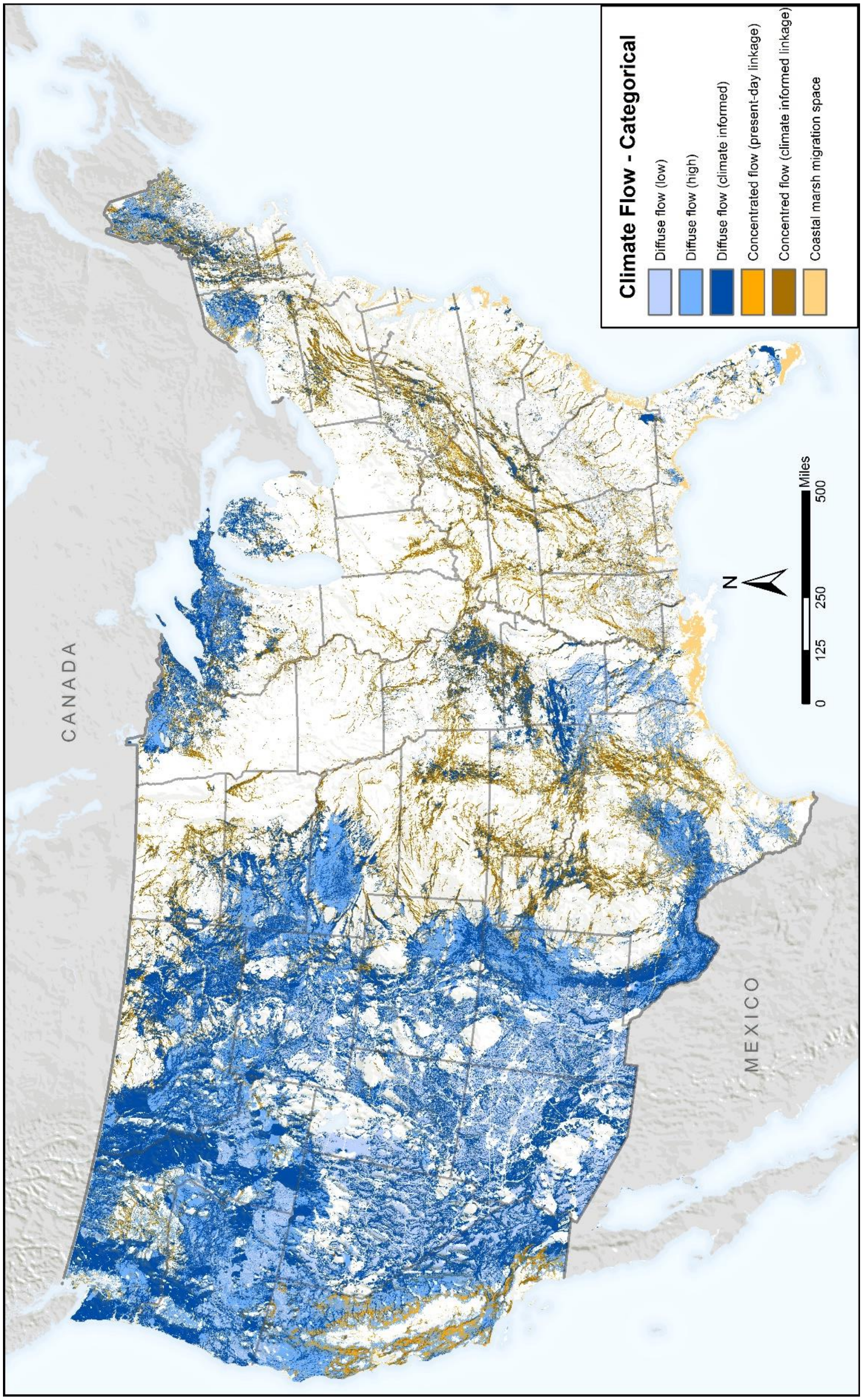

Figure 5. Climate Flow Categorical

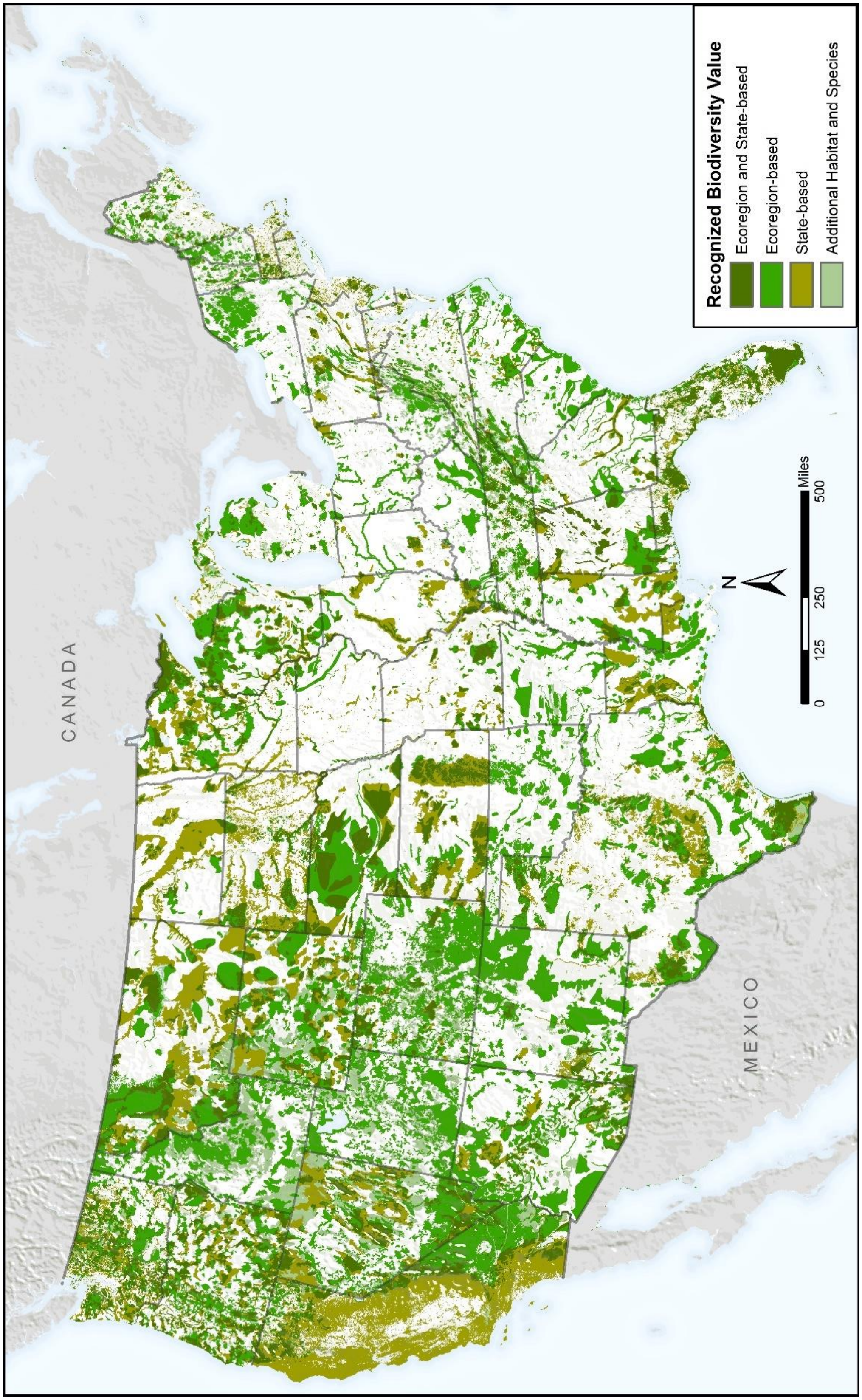

Figure 6. Recognized Biodiversity Value

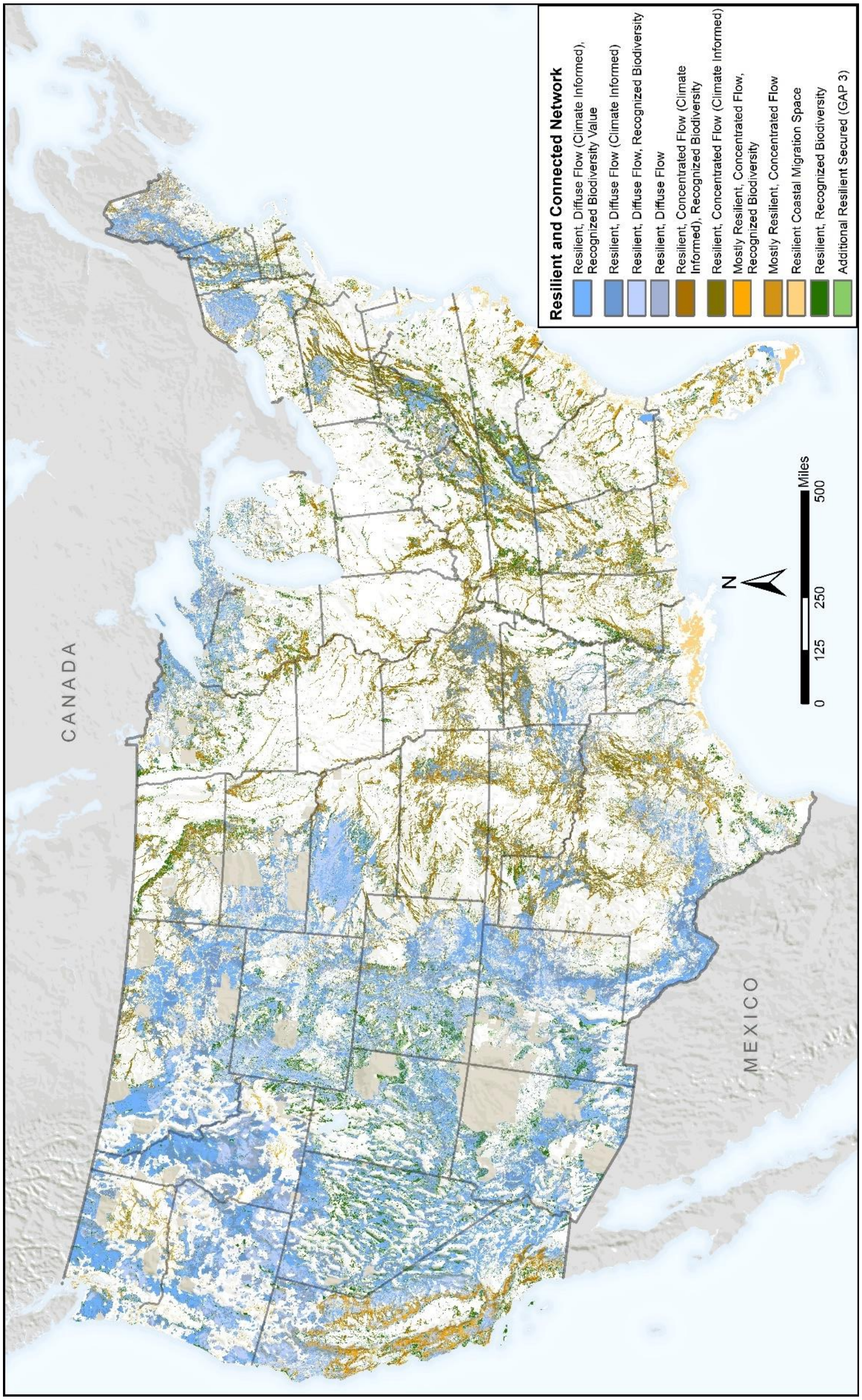

Figure 7. Resilient and Connected Network (detailed classes)

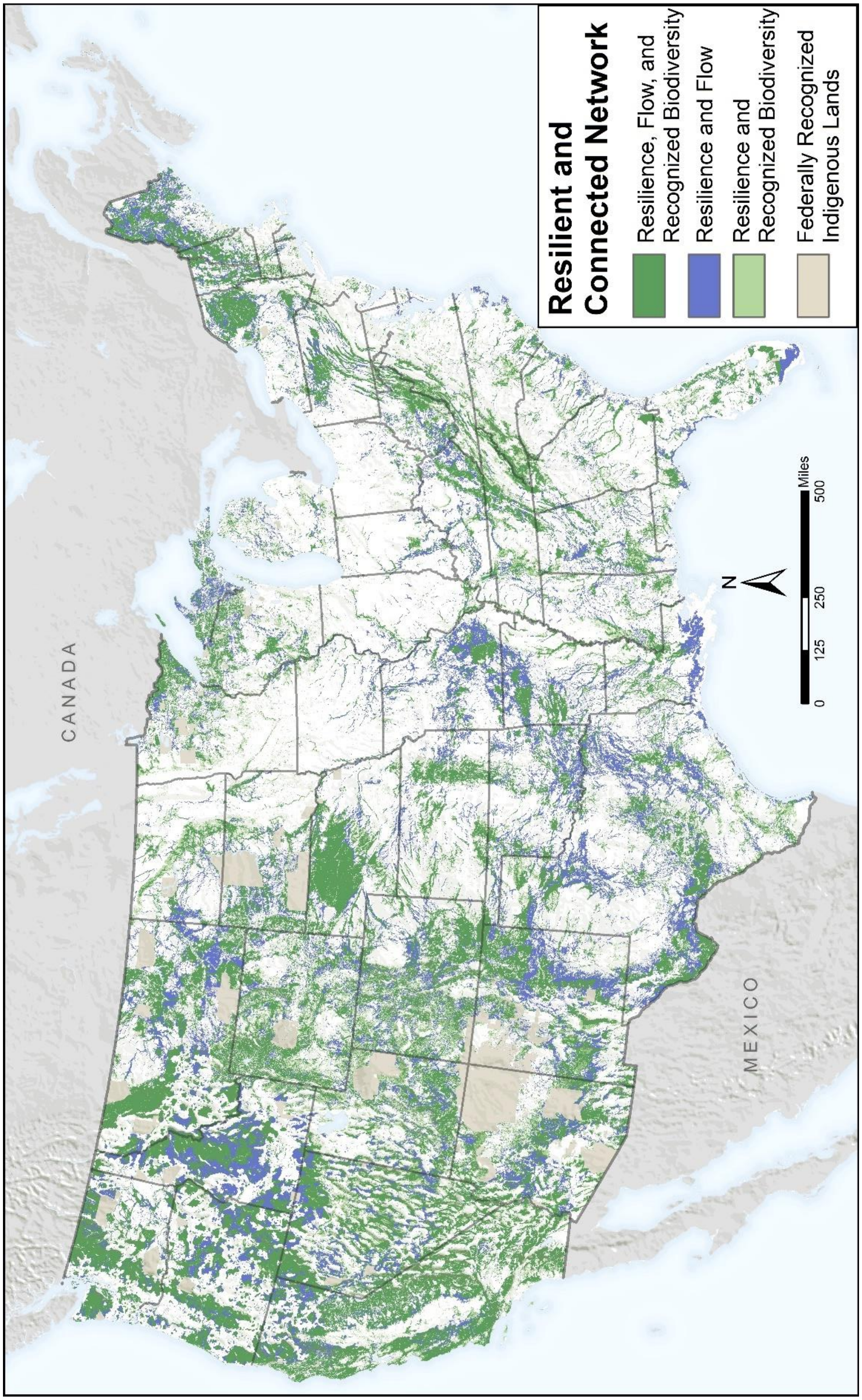

Figure 8. Resilient and Connected Network (summary classes)

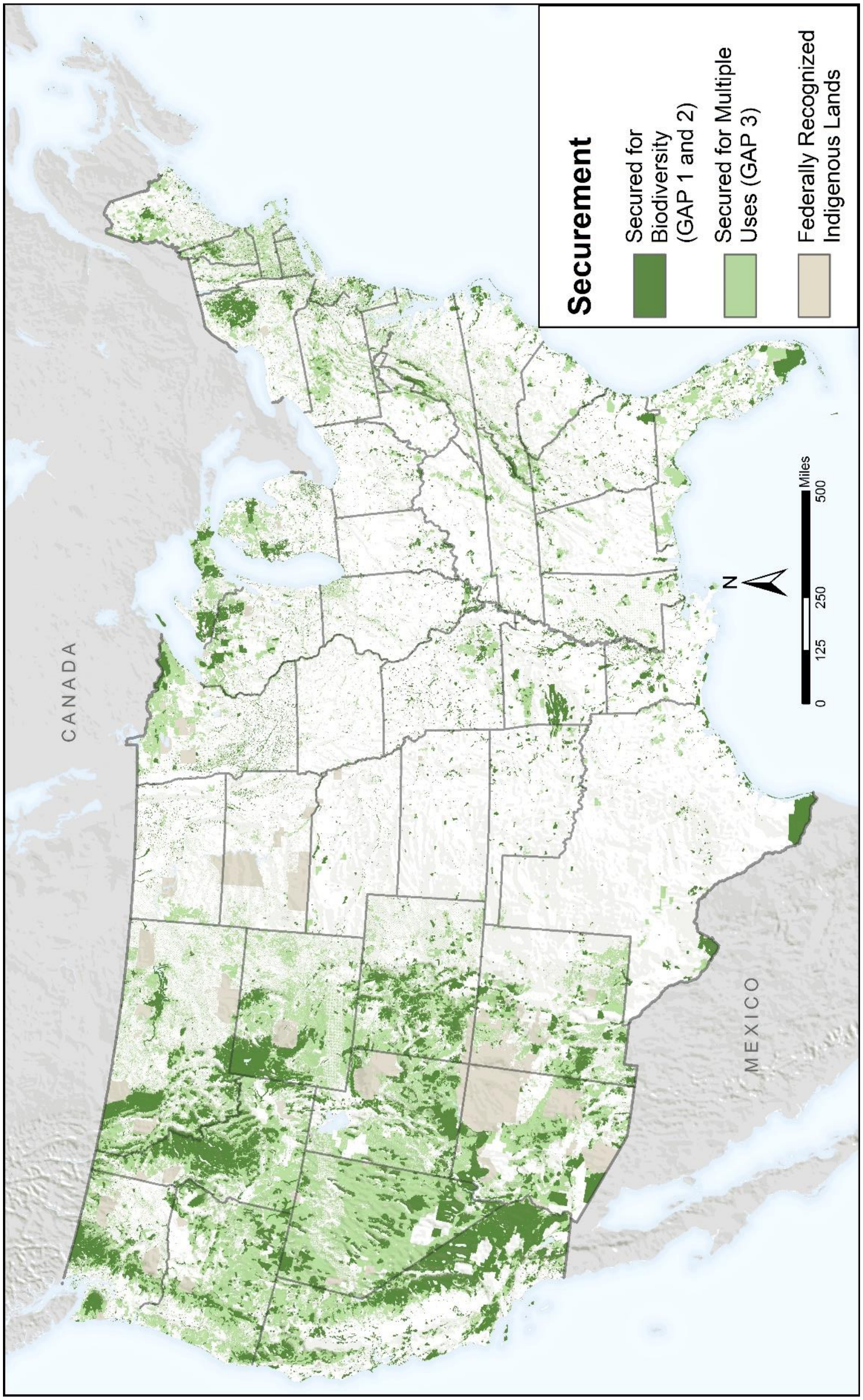

Figure 9. Secured Areas

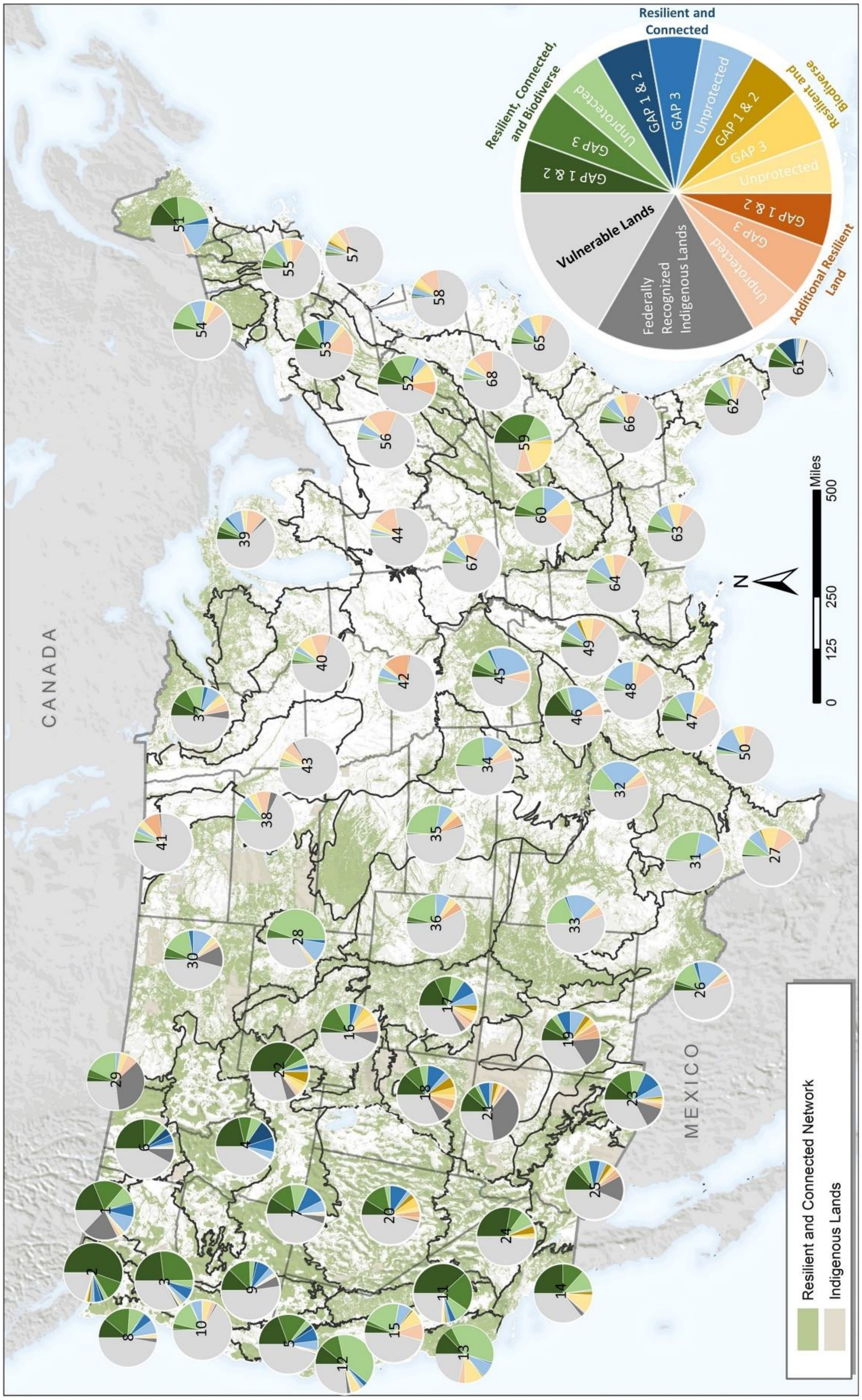

Figure 10a. Extent and Securement of the Resilient and Connected Network by Ecoregion

PACIFIC NORTHWEST

1 Okanagan
2 North Cascades
3 West Cascades
4 Middle Rockies - Blue Mountains
5 Klamath Mountains
6 Canadian Rocky Mountains
7 Columbia Plateau
8 Pacific Northwest Coast
9 East Cascades - Modoc Plateau
10 Willamette Valley - Puget Trough

CALIFORNIA

11 Sierra Nevada
12 California North Coast
13 California Central Coast
14 California South Coast
15 Great Central Valley

ROCKY MOUNTAINS

16 Utah-Wyoming Rocky Mountains
17 Southern Rocky Mountains

ROCKY MOUNTIANS (CONT.)

18 Utah High Plateaus
19 Arizona-New Mexico Mountains
20 Great Basin
21 Colorado Plateau
22 Wyoming Basins

WARM DESERTS/TAMAULIPAN

23 Apache Highlands
24 Mojave Desert
25 Sonoran Desert
26 Chihuahuan Desert
27 Tamaulipan Thorn Scrub

GREAT PLAINS

28 Black Hills
29 Fescue-Mixed Grass Prairie
30 Northern Great Plains Steppe
31 Edwards Plateau
32 Crosstimbers /S. Tallgrass Prairie
33 Southern Shortgrass Prairie
34 Osage Plains/Flint Hills Prairie

GREAT PLAINS (CONT.)

34 Osage Plains/Flint Hills Prairie
35 Central Mixed-Grass Prairie
36 Central Shortgrass Prairie

GREAT LAKES/TALLGRASS PRAIRIE

37 Superior Mixed Forest
38 Dakota Mixed-Grass Prairie
39 Great Lakes
40 Prairie-Forest Border
41 Aspen Parkland
42 Central Tallgrass Prairie
43 Northern Tallgrass Prairie
44 North Central Tillplain

LOWER MISSISSIPPI AND OZARKS

45 Ozarks
46 Ouachita Mountains
47 West Gulf Coastal Plain
48 Upper West Gulf Coastal Plain
49 Mississippi River Alluvial Plain
50 Gulf Coast Prairies and Marshes

EAST

51 Northern Appalachian / Acadian
52 Central Appalachian Forest
53 High Allegheny Plateau
54 St. Lawrence - Champlain Valley
55 Lower New England/N. Piedmont
56 Western Allegheny Plateau
57 North Atlantic Coast
58 Chesapeake Bay Lowlands
59 Southern Blue Ridge
60 Cumberlands/ S. Ridge & Valley
61 Tropical Florida
62 Florida Peninsula
63 East Gulf Coastal Plain
64 Upper East Gulf Coastal Plain
65 Mid-Atlantic Coastal Plain
66 South Atlantic Coastal Plain
67 Interior Low Plateau
68 Piedmont

Figure 10b. List of Ecoregions by number and name as shown on Figure 10a. Extent and Securement of the Resilient and Connected Network by Ecoregion

Table S1. Landscape diversity components and geophysical setting sources by region

Study Region	Components of Landscape Diversity							Components of Geophysical Settings			
	Land Position (TPI)	Slope and Aspect	Moisture Index C=CTI, F= FATHOM	Wetlands (NWI / NLCD)	Landform Types	Heat Load Index (HLI)	Elevation Range	Bedrock Classes	Soil Texture (T) or Orders (O)	Elevevation or Life Zones	Slope Classes
Northeast	Y	Y	C	Y	Y (17)		Y	B(7)	T(3)	E(6)	
Southeast	Y	Y	C	Y	Y (17)		Y	B(7)	T(3)	E(6)	
Great Lakes	Y	Y	C	Y	Y (17)			B(6)	T(5)	E(1)	
Mississippi	Y	Y	C	Y	Y (17)			B(7)	T(6)	E(3)	
Great Plains	Y	Y	C	Y	Y (17)			B(7)	T(4)	E(1)	
Rocky Mountains	Y	Y	F	Y	Y (21)		Y	B(8)	T(5)	LZ(8)	
Desert Southwest	Y	Y	F	Y	Y(21)		Y	B(8)	T(5)	LZ(8)	
Pacific Northwest			C	Y		Y		B(1)	O(10)	E(8)	S(3)
California			C			Y		B(1)	O(11)	E(8)	S(3)

Types of inputs with count of classes in parentheses

Geology Classes	
BEDROCK CLASSES	Acidic to Circumneutral Sedimentary, Calcareous Sedimentary, Caliche, Fissile Shale, Granitic, Mafic and Intermediate Granitic, Moderately Calcareous Sedimentary, Novaculite, Shale and Fine-Grained Sedimentary, Ultramafic, Volcanic Felsic, Unidentified Bedrock
SURFICIAL CLASSES	Acidic Loam, Calcareous Loam, Deep Loess, Gypum and Evaporite, Sand, Silt-Clay-Mixed, and Playa
SOIL ORDERS (only PNW and CA Regions)	Alfisol, Andisol, Aridisol, Entisol, Glacier, Histosol, Inceptisol, Mollisol, Oxisol, Spodosol, Ultisol, Vertisol

Elevation Zones		*Northeast*	*Southeast*	*Mississippi*		*California and Pacific Northwest*	
	Coastal	0-6 m	0-6 m	Coastal	0-6 m	Zone 1	0 - 600 m
	Very Low	6-244 m	6-244 m	Moderate	6-549 m	Zone 2	600 - 1200 m
	Low	244-518 m	244-518 m	High	>549 m	Zone 3	1200 - 1800 m
	Mid	518-762 m	518-762 m			Zone 4	1800 - 2400 m
	High	762-1097 m	762-1372 m			Zone 5	2400 - 3000 m
	Very High	>1097 m	>1372 m			Zone 6	3000 - 3600 m
						Zone 7	3600 - 4200 m
						Zone 8	4200 - 4800 m

Rocky Mountain and Desert Southwest: Life Zones	
Alpine	Life Zones from LANDFIRE (2014) with elevational adjustments.
Subalpine	
Upper Montane	
Lower Montane	
Mountain Lowlands	Areas below Lower Montane in following ecoregions: Arizona-New Mexico Mountains, Southern Rocky Mountains, Utah High Plateaus, Utah-Wyoming Rocky Mountains
Cold Desert Lowlands	Areas below Lower Montane in following ecoregions: Colorado Plateau, Great Basin, Wyoming Basins
Warm Desert Lowlands	Areas below Lower Montane in following ecoregions: Apache Highlands, Chihuahuan Desert, Mojave Desert, Sonoran Desert
Coastal/Tropical Lowlands	Areas below Lower Montane in following ecoregions: Tamaulipan Thorn Scrub

Data sources: USGS 30m Digital Elevation Data (1 arc-second) for elevation, slope, and topographically derived products such as Topographic Land Position - TPI (Fels 1995, Wilson and Gallant 2000), Compound Topographic Index - CTI (Moore et al. 1993), Heat Load Index - HLI (McCune and Keon, 2002), and Landforms (Anderson et al. 2016). Pluvial and fluvial 100yr flood zones from FATHOM, 2017. Wetlands from USFWS National Wetland Inventory and NLCD 2011, 2016; Bedrock from digital USGS state geologic maps ; soils from NRCS STATSGO or SSURGO or POLARIS (Princeton University, 2016)

Table S2. Regional Methods for Component Weighting and Integration into Landscape Diversity Scores

1. EASTERN REGION<br><br>To create a standardized metric of landscape diversity (LD) we transformed all three indices (landform variety (LV), elevation range (ER), and wetland density (WD) to standardized normal distributions ("Z-scores" with a mean of 0 and standard deviation of 1) then combined them into a single index.<br>In the combined index, we weighted landform variety twice as much as the other two values because of the importance of this feature in creating well defined microclimates. Further, wetland density was only added when the setting was a flat landform (dry flat, wet flat, slope bottom flat). The final index was:<br>Landscape Diversity on Flat Landforms = (2 LV + 1 ER + 1WD)/4<br>Landscape Diversity on Slopes = (2 LV + 1 ER)/3<br><br>Where<br>LV = Landform Variety = (1*# Landforms)<br>WD = Wetland Density = (2*Density in 0.41 ha) + 1* Density in 4.1 ha+ 1* #Wetland polygons in 0.41 ha) / 4<br>ER = Local Elevation Range = residual of elevation range in 0.41 ha circle regressed on LV
2. GREAT LAKES REGION<br><br>To create a final map of landscape diversity, we combined the landform variety score (LV) and the wetland influence score (WS) into a single index, using the transformed z-score values to ensure they were on the same scale. Landform variety was given twice the weight of wetland influence to reflect fundamental importance of microclimates to all types of terrestrial and wetland species. The wetland score was only added if the average combined value was higher with the wetland influence score than without it.<br><br>If LV > (LV+LV+WS)/3 then use LV<br>If LV < (LV+LV+WS)/3 then use (LV+LV+WS)/3<br><br>Where<br>LV = Landform Variety = (1*# Landforms)<br>WS = Wetland Influence Score which is based on the density (2*Density in 0.41 ha) + 1* Density in 4.1 ha) and patchiness (#Wetland polygons in 0.41 ha) of current wetlands to form the WD Score (2*Density in 0.41 ha) + 1* Density in 4.1 ha+ 1* #Wetland polygons in 0.41 ha) / 4 which was then combined with the connectedness of topographic basins and riparian areas to yield Final Wetland Influence Score = (WD Score + Connectedness of Wetland Topography Score) / 2

3. GREAT PLAINS REGION AND LOWER MISSISSIPPI REGION

To create a final score of landscape diversity, we combined the landform variety score and the wetland density score into a single index using the transformed Z-score values to ensure they were all data were on the same scale.
For each cell, we used the maximum of the following two options as the final landscape diversity score:
1. Landform Variety Z Score
2. Landform Variety Z + Wetland Density Z) / 2

Where
LV = Landform Variety = (1*# Landforms)
WD = Wetland Density = (2*Density in 0.41 ha) + 1* Density in 4.1 ha) / 3

By using the maximum value, the wetland density was only incorporated in cells where it increased the base landform variety score.

4. ROCKY MOUNTAINS DESERT SOUTHWEST REGION

To create a final map of landscape diversity, we created a regional score within the 3 regions and an ecoregion score within each of the 12 ecoregions.
For each cell, base score was the landform variety Z score within the given geography (region or ecoregion). The cell scores were then increased if they were identified by any of the boosting criteria for elevation, moisture, or wetland density. The boosts were limited to areas by comparing cell values to the landform variety score alone, and the magnitude of the boost varied depending on the magnitude of this difference. For example, to implement the wetland density boost, we subtracted the landform variety score from the wetland density score such that a positive difference indicated the wetland density was greater than the landform variety relative to their respective means. We then identified areas where wetland density was both 1) above the mean (>0.5 SD) and where 2) the difference between wetland density and landform variety was also above the mean (>0.5 SD) To these areas, we gave a slight boost (0.50 – 2 SD) to the landscape diversity score scaling the boost to the size of the difference. See the full report for more details on each boost.
Boosts varied between:
1. Elevation Range boost: 0.25-1 SD
2. Moist Landform boost: 0.25-1 SD
3. Wetland Density boost: 0.25-2 SD

The final Landscape Diversity score was equal to landscape variety score plus the sum of the boosts. This was then divided by the standard deviation of the ecoregion to appropriately spread out the distribution and approximate standard normal units.

5. PACIFIC NORTHWEST REGION

In the Pacific Northwest:
Landscape Diversity = (HLI index * CTI index) / STD (HLI index * CTI index).
Where:
HLI index = Heat Load Index range normalized 0-1,
CTI index = Compound Topographic Index normalized 0-1

6. CALIFORNIA

Landscape Diversity = ((HLI index*CTI index) + Maximum (HLI index, CTI index)).
Where:
HLI index = Heat Load Index range normalized 0-1,
CTI index = Compound Topographic Index normalized 0-1

Table S3. Resistance grid variables and data sources

Anthropogenic Components	Weight	Source
**Land Cover**		
Developed, High Intensity	20	NLCD 2011 (24)
Developed, Medium Intensity	9	NLCD 2011 (23)
Barren Land, non-natural	9	NLCD 2011 (31)
Developed, Open Space or Low Intensity	8	NLCD 2011 (21/22)
Cultivated Crops	7	NLCD 2011 (82)
Hay or Pasture	3	NLCD 2011
Natural (Forest, Shrub, Grassland, Wetland,Barren)	1	NLCD 2011 (32, 41-43, 52, 71, 81,90, 95)
**Structures**		
Building footprints	9*	Microsoft 2019
**Roads & Linear**		
Major Roads	20	Tiger 2016 & National Road Network
Minor Roads	10	Tiger 2016 & National Road Network
Dirt Roads	R+1	Open Street Map Tracks / Tiger Vehicular Trail
Railroads	9	CTS 2016
Transmission Lines	7	Ventyx 2017
Pipelines	9	Ventyx 2017
**Agriculture and Forestry**		
Industrial Agriculture (persistent Corn/Soy)	9	Cropscape 2016
Other Agriculture	7	Cropscape 2017
Industrial Forestry	4	Global Forest Watch
Forest Loss or Gain	3	Global Forest Change Dataset (2016)
Prairie/Grassland Areas	1	Nature Serve Eos, Remnant Prairies in Iowa,
Grassland/Pasture	3	Cropscape 2016: most years Grassland
**Energy infrastructure**		
Oil & Gas Wells	0-1*	State datasets -1,750,000 active/inactive wells,
Wind turbines	0-1*	27000 turbines, FAA Digital Obstacle File, U.S. Wind Turbine Database
Solar farms	20	U.S. EPA 2019
Surface mine/developed barrens	10	NLCD 32 with inspection
**Natural Components**		
**Waterbodies: Distance to Shoreline**		
<200 m /200-400m / >400	1/3/5	NLCD, NHD, NHN, ArcGIS Analysis
**Landforms**		
Cliff	10	DEM Landform Model
Steep slope	9	DEM Landform Model
Flat summit , Hilltop flat, Dry flat, Wet flat, SB flat	7	DEM Landform Model
Gentle slope , Slope bottom	4	DEM Landform Model
Slope crest, Sideslope, Cove, Low slope	1	DEM Landform Model
Downslope	0-1.5	DEM Landform Model + relative elevation grid

Table S4. Connectivity Modeling Approaches, Parameters, and Classification

Study Region	Model	Source	Resistance	Determining classes: Concentrated Flow	Determining classes: Diffuse	Incorporating climate: model	Incorporating climate: modified parameters
**Northeast, Southeast, Mississippi, Desert, Rocky Mountains, Great Plains, Great Lakes**	Wall-2-Wall Circuitscape (540 km tiles)	Current is injected uniformly across the landscape.	Assigned based on land cover and land use	High flow areas that had more flow when compared with the 1000ac neighborhood (i.e. high standard deviation)	High flow areas that had similar flow to the 1000ac neighborhood (i.e. low standard deviation)	Wall-2-Wall Circuitscape	Modeled movement towards cooler temperatures (upslope or northward movement) or wetter environments (downslope movement) by modifying resistance values (upslope/downslope) or weighting Circuitscape output from a south to north model run
**Pacific Northwest**	Omniscape (50km radius)	Assigned 0-1 values based on land cover and land use	Assigned based on land cover and land use	More flow (ratio >=1.3) than would be expected in the absence of barriers to movement	As much flow (ratio of 0.7 - 1.3) as would be expected in the absence of barriers to movement	Wall-2-Wall Circuitscape	Modeled movement towards cooler temperatures (upslope or northward movement) or wetter environments (downslope movement) by modifying resistance values (upslope/downslope) or weighting Circuitscape output from a south to north model run
**California**	Omniscape (50km radius)	Inverse of a human modification index; ranges between 0 and 1	squared human modification index	More flow (ratio >=1.3) than would be expected in the absence of barriers to movement	As much flow (ratio of 0.7 - 1.3) as would be expected in the absence of barriers to movement	Omniscape	Modeled movement from current climate to future similar climate based on 2050 projections by modifying source and along microcliamte stepping stones by modifying resistance values in areas with high topodiversity

Table S5. Recognized biodiversity data sources

A. Summary Component Table

Study Region	Components			
	TNC Portfolio Sites	State Wildlife Action Plans or other statewide biodiversity data	Additional Habitat or Species Areas	GAP 1 and 2 Secured Lands
Northeast	Y	Y	Y	Y
Southeast	Y	Y	Y	Y
Great Lakes	Y	Y		Y
Mississippi	Y	Y		Y
Great Plains	Y	Y	Y	Y
Rocky Mountains	Y	Y	Y	Y
Deserts	Y	Y	Y	Y
Pacific Northwest	Y	Y		Y
California		Y		Y

B. TNC Ecoregional Plan Sources

Ecoregion	Year	Citation	Primary Report Link	Additional Report and Data link
Apache Highlands	2004	Marshall, R.M., D. Turner, A. Gondor, D. Gori, C. Enquist, G. Luna, R. Paredes Aguilar, S. Anderson, S. Schwartz, C. Watts, E. Lopez, P. Comer. 2004. An Ecological Analysis of Conservation Priorities in the Apache Highlands Ecoregion. Prepared by The Nature Conservancy of Arizona, Instituto del Medio Ambiente y el Desarrollo Sustentable del Estado de Sonora , Agency and Institutional partners. 152.pp.	Report	-
Arizona-New Mexico Mountains	1999	Bell, G, J. Baumgartner, J. Humke, A. Laurenzi, P. McCarthy, P. Mehlhop, K. Rich, M. Silbert, E. Smith, B. Spicer, T. Sullivan, and S. Yanoff. 1999. Ecoregional Conservation Analysis of the Arizon-New Mexico Mountains. Arizona-New Mexico Ecoregional Conservation Team. The Nature Conservancy. Santa Fe, New Mexico.	Report	-
Aspen Parkland	2007	Riley, J.L, S.E. Green and K.E. Brodribb. 2007. A Conservation Blueprint for Canada's Prairies and Parklands. Nature Conservancy of Canada, Toronto, Ontario.	Report	
Black Hills	2000	Hall, J., H. Marriott, and J. Perot. 2002. Ecoregional Conservation in the Black Hills. The Nature Conservancy. Midwest Conservation Science Center. Minneapolis, MN.	Report	-

California North Coast	2001	The Nature Conservancy. 2001. California North Coast Ecoregional Plan. The Nature Conservancy, California Field Office. San Francisco, CA	Report	-
Canadian Rocky Mountains	2004	The Nature Conservancy. 2004. Candian Rocky Mountains Ecoregional Assessment. Volume One: Report Version 2.0.	Report	-
Central Appalachian Forest	2001	Anderson, M.G., A. Olivero, C. Ferree, D. Morse, S. Khanna, and S. Bernstein. 2001. Central Appalachian Forest Ecoregional Plan. The Central Appalachian Ecoregion: Ecoregional Assessment, Conservation Status and Resource CD. The Nature Conservancy. Boston, MA.	Report	Additional Link
Central Mixed-Grass Prairie	2003	Steuter Al, Jennifer S. Hall and Mary Lammert Khoury. 2003. Conserving the biological diversity of the Central Mixed-Grass Prairie: A portfolio designed for conservation action. The Nature Conservancy, Nebraska Field Office, Omaha NE.	Report	-
Central Shortgrass Prairie	2006	Neely, B., S. Kettler, J. Horsman, C. Pague, R. Rondeau, R. Smith, L. Grunau, P. Comer, G. Belew, F. Pusateri, B. Rosenlund, D. Runner, K. Sochi, J. Sovell, D. Anderson, T. Jackson and M. Klavetter. 2006. Central Shortgrass Prairie Ecoregional Assessment and Partnership Initiative. The Nature Conservancy of Colorado and the Shortgrass Prairie Partnership. 124 pp. and Appendices.	Report	-
Central Tallgrass Prairie	2008	The Nature Conservancy. 2008. Central Tallgrass Prairie Ecoregion Assessment: Update on Biodiversity. The Nature Conservancy, Missouri Field Office. St. Louis, MO	Report	-
Chesapeake Bay Lowlands	2003	Samson, D.A., M.G. Anderson et al. 2003. Chesapeake Bay Lowlands Ecoregional Conservation Plan; First Iteration, Edited. The Nature Conservancy, Mid-Atlantic Division, Charlottesville, VA	Report	Additional Link
Chihuahuan Desert	2004	The Nature Conservancy, 2004. Ecoregional Conservation Assessment of the Chichuahan Desert. Second Edition Revised 2004. Pronatura In partnership with The Nature Conservancy and The World Wildlife Fund.	Report	-

Colorado Plateau	2002	Tuhy, J., P. Comer, G. Bell, D. Dorfman, B. Neely, M. Lammert, S. Silbert, H. Humke, L. Whitham, B. Cholvin, and B. Baker. 2002. A Conservation Assessment of the Colorado Plateau Ecoregion. The Nature Conservancy Colardo Plateau Ecoregional Planning Team. Moab. Utah.		-
Columbia Plateau	2003	The Nature Conservancy. 1999 (revised 2003). The Columbia Plateau Ecoregional Assessment: A Pilot Effort in Ecoregional Conservation. The Nature Conservancy's Columbia Plateau Ecoregional Planning Team.	Report	-
Crosstimbers And Southern Tallgrass Prairie	2009	The Nature Conservancy. 2009. A Conservation Blueprint for the Crosstimbers & Southern Tallgrass Prairie Ecoregion. CSTP Ecoregional Planning Team, The Nature Conservancy, San Antonio, TX.	Report	-
Cumberlands And Southern Ridge And Valley	2003, 2013 Update in AL, GA, TN & KY.	The Nature Conservancy, 2003. The Cumberlands and Southern Ridge & Valley Ecoregion: A Plan for Biodiversity Conservation. The Nature Conservancy. Arlington, Virginia.; 2013 Updated Southeastern U.S. Terrestrial Portfolios. Eastern Conservation Science team of The Nature Conservancy, TNC State Chapter Science and Protection staff of AL, FL, GA, SC, NC, TN, KY.	Report	Additional Link
Cypress Upland	2007	J.L. Riley, S.E. Green and K.E. Brodribb. 2007. A Conservation Blueprint for Canada's Prairies and Parklands. Nature Conservancy of Canada, Toronto, Ontario.	Report	
Dakota Mixed-Grass Prairie	2010	Harkness, Mary, Jennifer S. Hall, Paula Gagnon, Phil Gerla, Meredith W. Cornett, Brian Schreurs, and Sarah Eichhorst. 2010. Conserving the biological diversity of the Dakota MixedGrass Prairie. The Nature Conservancy, Minneapolis MN.	Report	-

East Cascades - Modoc Plateau	2007	Popper, K., G. Wilhere, M. Schindel, D. VanderSchaaf, P. Skidmore, G. Stroud, J. Crandall,<br>J. Kagan, R. Crawford, G. Kittel, J. Azerrad, L. Bach. 2007. The East Cascades - Modoc Plateau and West Cascades Ecoregional Assessments. Prepared by The Nature Conservancy<br>and the Washington Department of Fish and Wildlife with support from the Oregon Natural Heritage Information Center, Washington Heritage Program, and Natureserve. The Nature<br>Conservancy, Portland, Oregon.	Main Report	-
East Gulf Coastal Plain	1999, 2013 Update in AL, FL & GA.	The Nature Conservancy. 1999 (revised 2001). East Gulf Coastal Plain Ecoregional Plan. East Gulf Coastal Plain Core Team.; 2013 Updated Southeastern U.S. Terrestrial Portfolios. Eastern Conservation Science team of The Nature Conservancy, TNC State Chapter Science and Protection staff of AL, FL, GA, SC, NC, TN, KY.	Report	Additional Link
Edwards Plateau	2004	The Nature Conservancy. 2004. A Biodiversity and Conservation Assessment of the Edwards Plateau Ecoregion.<br>Edwards Plateau Ecoregional Planning Team, The Nature Conservancy, San Antonio, TX, USA	Report	-
Fescue-Mixed Grass Prairie	2007	J.L. Riley, S.E. Green and K.E. Brodribb. 2007. A Conservation Blueprint for Canada's Prairies and Parklands.<br>Nature Conservancy of Canada, Toronto, Ontario. 226 pp. plus DVD-ROM.	Report	
Florida Peninsula	2005, 2013 Update	A Conservation Blueprint for Canada's Prairies and Parklands.	Report	Additional Link
Great Basin	2001	Nachlinger, J., K. Sochi, P. Comer, G. Kittel, and D. Dorfman. 2001. Great Basin: an ecoregion-based conservation blueprint. The Nature Conservancy, Reno, NV. 160 pp. + appendices.	Report	-
Great Lakes: US	2000	The Nature Conservancy. 2000. Toward a New Conservation Vision for the Great Lakes Region: A Second Iteration Ecoregional Plan. The Nature Conservancy, Great Lakes Program. Chicago, IL;	Report	
Great Lakes: Canada	2005	Henson, B.L, K.E. Brodribb, and J.L. Riley 2005. Great Lakes Conservation Blueprint for Terrestrial Biodiversity. Vol. 1 and Vol. 2. Nature Conservancy of Canada.	Report	
Gulf Coast Prairies And Marshes	2002	The Nature Conservancy. 2002. The Gulf Coast Prairies and Marshes Ecoregional Conservation Plan. Gulf Coast Prairies and Marshes Ecoregional Planning Team, The Nature Conservancy, San Antonio, TX, USA.	Report	-

High Allegheny Plateau	2004	Zaremba, R., M.G. Anderson, A. Olivero, D. Morse, S. Khanna, and S. Bernstein. 2002. The High Allegheny Plateau Ecoregional Plan: Ecoregional Assessment, Conservation Status and Resource CD. The Nature Conservancy. Boston, MA.	Report	Additional Link
Interior Low Plateau	2001, 2013 Update in TN & KY.	The Nature Conservancy. 2001. The Interior Low Plateau Ecoregion: A Conservation Plan. The Interior Low Plateau Eoregional Planning Team.; 2013 Updated Southeastern U.S. Terrestrial Portfolios. Eastern Conservation Science team of The Nature Conservancy, TNC State Chapter Science and Protection staff of AL, FL, GA, SC, NC, TN, KY.	Report	Additional Link
Klamath Mountains	2003	Vander Schaaf, D., M. Schindel, D. Borgias, C. Mayer, D. Tolman, G. Kittel, J. Kagan, T. Keeler-Wolf, L. Serpa, J. Hak, K. Popper. 2004. Klamath Mountains Ecoregional Conservation Assessment. The Nature Conservancy. Portland, Oregon.	Report	-
Lower New England / Northern Piedmont	2003	Anderson, M.G., A. Olivero, D. Morse, S. Khanna and S. Bernstein. 2003. The Lower New England/Northern Piedmont Ecoregion: Ecoregional Assessment, Conservation Status and Resource CD. The Nature Conservancy. Boston, MA.	Report	Additional Link
Mid-Atlantic Coastal Plain	2001, 2013 Update in SC & NC.	The Nature Conservancy. 2001. Mid-Atlantic Coastal Plain Ecoregional Plan. The Core Ecoregional Planning Team and Southeastern Regional Office of The Nature Conservancy. ; 2013 Updated Southeastern U.S. Terrestrial Portfolios. Eastern Conservation Science team of The Nature Conservancy, TNC State Chapter Science and Protection staff of AL, FL, GA, SC, NC, TN, KY.	Report	Additional Link
Middle Rockies - Blue Mountains	2000	The Nature Conservancy. 2000. Middle Rockies-Blue Mountains Ecoregional Conservation Plan. Middle Rockies – Blue Mountains Planning Team.	Report	-
Mississippi River Alluvial Plain	2002	The Nature Conservancy. 2002. Conservation Planning in the Mississippi River Alluvial Plain. The Nature Conservancy. Baton Rouge, LA, USA.	Report	-
Mojave Desert	2010	Randall, J. M., S.S. Parker, J. Moore, B. Cohen, L. Crane, B. Christian, D. Cameron, J. MacKenzie, K. Klausmeyer and S. Morrison. 2010. Mojave Desert Ecoregional Assessment. Unpublished Report. The Nature Conservancy, San Francisco, California. 106 pages + appendices.	Report	-

North Atlantic Coast	2006	Anderson, M.G., C. Ferree, D. Morse, A. Olivero, S. Khanna, and S. Bernstein. 2006. The North Atlantic Coast Ecoregion: Ecoregional Assessment, Conservation Status and Resource CD. The Nature Conservancy. Boston, MA.	Report	Additional Link
North Cascades	2007	Iachetti, P., J. Floberg, G. Wilhere, K. Ciruna, D. Markovic, J. Lewis, M. Heiner, G. Kittel, R. Crawford, S. Farone, S. Ford, M. Goering, D. Nicolson, S. Tyler, and P. Skidmore. 2006. North Cascades and Pacific Ranges Ecoregional Assessment, Volume 1 - Report. Prepared by the Nature Conservancy of Canada, The Nature Conservancy of Washington, and the Washington Department of Fish and Wildlife with support from the British Columbia Conservation Data Centre, Washington Department of Natural Resources Natural Heritage Program, and NatureServe. Nature Conservancy of Canada, Victoria, BC.	Main Report	-
North Central Tillplain	2003	The Nature Conservancy. 2003. The North Central tillplain Ecoregion: A Conservation Plan. North Central Tillplain Ecoregional Planning Team.	Report	-
Northern Appalachian / Acadian	2006	Anderson, M.G., B. Vickery, M. Gorman, L. Gratton, M. Morrison, J. Mailet, A. Olivero, C. Ferree, D. Morse, Kehm, G., Rosalska, K., Khanna, S., and S. Bernstein. 2006. The Northern Appalachian / Acadian Ecoregion: Ecoregional Assessment, Conservation Status and Resource CD. The Nature Conservancy. Boston, MA.	Report	Additional Link
Northern Great Plains Steppe	1998 (US), 2007 (Canada)	The Nature Conservancy. 1999. Ecoregional Planning in the Northern Great Plains Steppe. Northern Great Plains Steppe Ecoregional Planning Team. ; J.L. Riley, S.E. Green and K.E. Brodribb. 2007. A Conservation Blueprint for Canada's Prairies and Parklands. Nature Conservancy of Canada, Toronto, Ontario.	Report	Additional Link

Northern Tallgrass Prairie	1999	The Nature Conservancy, Northern Tallgrass Prairie Ecoregional Planning Team. 1998. Ecoregional planning in the Northern Tallgrass Prairie ecoregion. The Nature Conservancy, Midwest Regional Office, Minneapolis, MN, USA. 208 pp.+ iv.	Report	-
Okanagan	2007	Nature Conservancy of Canada and The Nature Conservancy of Washington, and the Washington Department of Fish and Wildlife. 2006. Okanagan Ecoregional Assessment.	Executive Summary	-
Osage Plains/Flint Hills Prairie	2000	The Nature Conservancy, Osage Plains/Flint Hills Prairie Ecoregional Planning Team. 2000. Ecoregional Conservation in the Osage Plains/Flint Hills Prairie. The Nature Conservancy, Midwestern Resource Office, Minneapolis, MN. 48 pp. + 73 appendices.	Report	-
Ouachita Mountains	2003	The Nature Conservancy. 2003. Ouachita Mountains Ecoregional Assessment. Ouachita Ecoregional Assessment Team. The Nature Conservancy, Little Rock, AR. Tulsa, OK. USA.	Report	-
Ozarks	2003	The Nature Conservancy, Ozarks Ecoregional Assessment Team. 2003. Ozarks Ecoregional Conservation Assessment. Minneapolis, MN: The Nature Conservancy Midwestern Resource Office. USA.	Report	-
Pacific Northwest Coast	2006	Vander Schaaf, D., G. Wilhere, Z. Ferdaña, K. Popper, M. Schindel, P. Skidmore, D. Rolph, P. Iachetti, G. Kittel, R. Crawford, D. Pickering, and J. Christy. 2006. Pacific Northwest Coast Ecoregion Assessment. Prepared by The Nature Conservancy, the Nature Conservancy of Canada, and the Washington Department of Fish and Wildlife. The Nature Conservancy, Portland, Oregon.	Report	-
Piedmont	2005, 2013 Update in AL, GA, SC & NC.	The Nature Conservancy, 2005. The Piedmont Ecoregion: A Plan for Biodiversity Conservation – Draft Implementation Document. The Nature Conservancy. Arlington, Virginia.; 2013 Updated Southeastern U.S. Terrestrial Portfolios. Eastern Conservation Science team of The Nature Conservancy, TNC State Chapter Science and Protection staff of AL, FL, GA, SC, NC, TN, KY.	Report	Additional Link
Prairie-Forest Border	2000	The Nature Conservancy. 2000. The Prairie-Forest Border Ecoregion: A Conservation Plan. The Prairie-Forest Border Ecoregion Core Team.	Report	-

Sonoran Desert	2000	Marshall, R.M., S. Anderson, M. Batcher, P. Comer, S. Cornelius, R. Cox, A. Gondor, D. Gori, J. Humke, R. Paredes Aguilar, I.E. Parra, S. Schwartz. 2000. An Ecological Analysis of Conservation Priorities in the Sonoran Desert Ecoregion. Prepared by The Nature Conservancy Arizona Chapter, Sonoran Institute, and Instituto del Medio Ambiente y el Desarrollo Sustentable del Estado de Sonora with support from Department of Defense Legacy Program, Agency and Institutional partners. 146 pp	Report	-
South Atlantic Coastal Plain	2002, 2013 Update in FL, GA, & SC.	The Nature Conservancy. 2002. South Atlantic Coastal Plain Ecoregion Plan. South Atlantic Coastal Plain Ecoregion Plan South Atlantic Coastal Plain Ecoregional Planning Team. ; 2013 Updated Southeastern U.S. Terrestrial Portfolios. Eastern Conservation Science team of The Nature Conservancy, TNC State Chapter Science and Protection staff of AL, FL, GA, SC, NC, TN, KY.	Report	Additional Link
Southern Blue Ridge	2000, 2013 Update in NC, TN, GA, & SC.	The Nature Conservancy and Southern Appalachian Forest Coalition. 2000. Southern Blue Ridge Ecoregional Conservation Plan: Summary and Implementation Document. The Nature Conservancy: Durham, North Carolina. ; 2013 Updated Southeastern U.S. Terrestrial Portfolios. Eastern Conservation Science team of The Nature Conservancy, TNC State Chapter Science and Protection staff of AL, FL, GA, SC, NC, TN, KY.	Report	Additional Link
Southern Rocky Mountains	2001	Neely, B., P. Comer, C. Moritz, M. Lammert, R. Rondeau, C. Pague, G. Bell, H. Copeland, J. Humke, S. Spackman, T. Schulz, D. Theobald, and L. Valutis. 2001. Southern Rocky Mountains: An Ecoregional Assessment and Conservation Blueprint. Prepared by The Nature Conservancy with support from the U.S. Forest Service, Rocky Mountain Region, Colorado Division of Wildlife, and Bureau of Land Management.	Report	-
Southern Shortgrass Prairie	2007	The Nature Conservancy. 2007. A Biodiversity and Conservation Assessment of the Southern Shortgrass Prairie Ecoregion. Southern Shortgrass Prairie Ecoregional Planning Team, The Nature Conservancy, San Antonio, TX.	Report	-
St. Lawrence - Champlain Valley	2003	Anderson, M.G., C. Ferree, A. Olivero, S. Khanna, and S. Bernstein. 2003. The St. Lawrence Ecoregion: Ecoregional Assessment, Conservation Status and Resource CD. The Nature Conservancy. Boston, MA.	Report	Additional Link

Superior Mixed Forest	2002	Van Helden, N., K. Bassler, and M. Madsen. 2002. The Superior Mixed Forest Ecoregion: A Conservation Plan. Core Team included The Nature Conservancy, Nature Conservancy of Canada, Ontario Ministry of Natural Resources, and Manitoba Conservation Data Centre.	Report	-
Tamaulipan Thorn Scrub	2010	The Nature Conservancy and Pronatura Noreste. 2010. A Conservation Blueprint for the Tamaulipan Thornscrub Ecoregion. Tamaulipan Thornscrub Ecoregional Planning Team, The Nature Conservancy, San Antonio, TX.		-
Tropical Florida	2005, 2013 Update.	The Nature Conservancy. 2004. Tropical Florida Ecoregional Plan. The Core Technical and Planning Team The Nature Conservancy & The University of Florida Geoplan Center. Tallahassee and Gainesville, Florida. ; 2013 Updated Southeastern U.S. Terrestrial Portfolios. Eastern Conservation Science team of The Nature Conservancy, TNC State Chapter Science and Protection staff of AL, FL, GA, SC, NC, TN, KY.	Report	Additional Link
Upper East Gulf Coastal Plain	2003, 2013 Update in AL, GA, TN & KY.	The Nature Conservancy & NatureServe, 2003. The Upper East Gulf Coastal Plain: An Ecoregional Assessment. ; 2013 Updated Southeastern U.S. Terrestrial Portfolios. Eastern Conservation Science team of The Nature Conservancy, TNC State Chapter Science and Protection staff of AL, FL, GA, SC, NC, TN, KY.	Report	Additional Link
Upper West Gulf Coastal Plain	2002	The Nature Conservancy. 2002. Upper West Gulf Coastal Plain Ecoregional Plan. Final Implementation Draft Prepared by Dave Gosse, Russell McDowell, Rob Evans and the UWGCP Technical and Planning Teams.	Report	-
Utah High Plateaus	2006	Comer, P., Tuhy, J. and R. Esselman, 2006. Scenario Building in the Utah High Plateaus Ecoregion. Case Study in Ecoregion Asessments and Biodiverity Vision Toolbox. The Nature Conservancy		-
Utah-Wyoming Rocky Mountains	2001	Noss, R., Wuerthner, G, Vance-Borland, K., and Carroll, C. 2001. A Biological Conservation Assessment for the Utah-Wyoming Rocky Mountains Ecoregion: Report to The Nature Conservancy. Conservation Science, Inc. Corvallis, OR. USA.	Report	-

West Cascades	2006	Popper, K., G. Wilhere, M. Schindel, D. VanderSchaaf, P. Skidmore, G. Stroud, J. Crandall,<br>J. Kagan, R. Crawford, G. Kittel, J. Azerrad, L. Bach. 2007. The East Cascades - Modoc Plateau and West Cascades Ecoregional Assessments. Prepared by The Nature Conservancy<br>and the Washington Department of Fish and Wildlife with support from the Oregon Natural Heritage Information Center, Washington Heritage Program, and Natureserve. The Nature<br>Conservancy, Portland, Oregon.	Report	-
West Gulf Coastal Plain	2003	The Nature Conservancy. 2003. The West Gulf Coastal Plain Ecoregional Conservation Plan. Wesst Gulf Coastal Plain Ecoregional Planning Team, The Nature Conservancy, San Antonio, TX, USA.	Report	-
Western Allegheny Plateau	2005	The Nature Conservancy. 2000 Draft Report (Datasets 2005). Ecoregional Plan for the Western Allegheny Plateau. The Nature Conservancy, Ohio Chapter. Dublin, OH	Draft Report.	
Willamette Valley - Puget Trough - Georgia Basin	2004	Floberg, J., M. Goering, G. Wilhere, C. MacDonald, C. Chappell, C. Rumsey, Z. Ferdana, A. Holt, P. Skidmore, T. Horsman, E. Alverson, C. Tanner, M. Bryer, P. Iachetti, A. Harcombe, B. McDonald, T. Cook, M. Summers, D. Rolph. 2004.<br>Willamette Valley-Puget Trough-Georgia Basin Ecoregional Assessment, Volume One: Report. Prepared by The Nature Conservancy with support from the Nature Conservancy of Canada, Washington Department of Fish and Wildlife,<br>Washington Department of Natural Resources (Natural Heritage and Nearshore Habitat programs), Oregon State Natural Heritage Information Center and the British Columbia Conservation Data Centre.	Report	-
Wyoming Basins	2013	Sochi, K., M. Heiner, H. Copeland, A. Pocewicz, and J. Keisecker. 2013. Systematic Conservation Planning in the Wyoming Basins. The Nature Conservancy. Boulder, CO. 134pp.		

C. State Sources

State	State Name	State Based Assessment Included	State & plan date, Title of map	Comments & link to the plan and dataset if publicly posted Abbreviations: SWAP = State Wildlife Action Plan, COA = Conservation Opportunity Areas, SCGN = Species of Greatest Conservation Need, TNC= The Nature Conservancy
AL	Alabama	Y	Alabama (2017). SWAP.	SWAP areas based on TNC Original Portfolio.
AZ	Arizona	Y	Arizona (2004). Native Grasslands in high quality	No Statewide SWAP available. Used portions of statewide grasslands study: http://azconservation.org/downloads/category/grassland_assessment A GIS data set depicting the results of a two-year study to delineate grasslands and evaluate their ecological condition in Arizona, southwestern New Mexico, and northern Mexico. This study was completed with the assistance of resource professionals from U.S. and Mexico universities and public agencies. We extracted class "A", "B", "A&B", these are native grasslands based on this statewide field survey. The Nature Conservancy. Arizona. 2004.
AR	Arkansas	N	Arkansas (2015): None.	In the plan they rank the ecoregions by number of SGCN (Fig 3.3 in the SWAP), but do not present mapped priorities at more local scales. http://www.wildlifearkansas.com
CA	California	Y	California Bird Species Richness Index from Modeling Bird Distribution Responses to Climate Change. 2010. Point Blue Conservation Science.	Recognized Biodiversity Value is based on the species richness index for the historic time period and includes the areas with the top 10% richness index in the state and the top 5% richness index within each ecoregion. http://climate.calcommons.org/dataset/14
CA	California	Y	California Amphibian and Reptile Richness from Wright et al. 2013. California Amphibian and Reptile Species of Future Concern: Conservation and Climate Change. California Department of Fish and Wildlife.	Recognized Biodiversity Value is based on species richness for the historic time period and includes the top 10% richest areas in the state and the top 5% richest areas within each ecoregion for each taxa. https://nrm.dfg.ca.gov/FileHandler.ashx?DocumentID=83972

CA	California	Y	California Mammal Richness Index from Stewart et al. 2016. A Climate Change Vulnerability Assessment for Twenty California Mammal Taxa. California Department of Fish and Wildlife.	Recognized Biodiversity Value is based on a species richness index calculated from the Species Distribution Models described in this report, but for all mammals in CA using the methodology described in 'A Climate Change Vulnerability Assessment for Twenty California Mammal Taxa'. Recognized Biodiversity Value is based on species richness for the historic time period and includes the top 10% richest areas in the state and the top 5% richest areas within each ecoregion for each taxa. https://nrm.dfg.ca.gov/FileHandler.ashx?DocumentID=135825&inline
CA	California	Y	Plant Species Richness Index and Range-restricted Endemic Species Richness Index from Kling et al. 2018. Facets of phylodiversity: evolutionary diversification, divergence and survival as conservation targets. Philosophical Transactions of the Royal Society B Biological Sciences.	Recognized Biodiversity Value is based on a species richness index and a range-restricted endemic species richness index and includes the areas with the top 20% of values in the state and the top 5% values within each ecoregion for each dataset. https://royalsocietypublishing.org/doi/full/10.1098/rstb.2017.0397
CA	California	Y	Rarity-weighted Occurrence Density based on observation from the California Natural Diversity Database. 2018. California Department of Fish and Wildlife.	Recognized Biodiversity Value is based on the top 80% of values from rarity weighted recent occurrence density within 1km of observations. https://wildlife.ca.gov/data/cnddb
CO	Colorado	Y	Colorado (2015): Crucial Habitat for Tier 1 Terrestrial Animal and Plant SGCN (Figure 21).	The state was mapped into 5 priority levels for crucial habitat for SGCN, and we incorporated the two highest levels into our composite SWAP map. Details on the map methodology are in Chapter 8 of the Colorado plan. http://cpw.state.co.us/aboutus/Pages/StateWildlifeActionPlan.aspx

CT	Connecticut	Y	Connecticut (2019). Natural Diversity Areas.	Natural Diversity Areas. The State of Connecticut, Department of Energy and Environmental Protection. June 2019. The Natural Diversity Database Areas is a 1:24,000-scale, polygon feature-based layer that represents general locations of endangered, threatened and special concern species and significant natural communities. The layer includes state and federally listed species and significant natural communities. It does not include Natural Area Preserves, designated wetland areas or wildlife concentration areas. These data are recognized by the State of Connecticut supporting biodiversity and was used for this purpose in the state's SWAP.
DE	Delaware	N		
DC	District of Columbia	N		
FL	Florida	Y	Florida (2016) Priority 1 and 2 CLIP V.4 Biodiversity Resource Category Priorities Model	The Florida biodiversity layer is from: Critical Lands and Waters Identification Project (CLIP) Version 4.0 Biodiversity Resource Category Priorities Model. The CLIP version 4.0 model combines conservation priorities from the SHCA, Vertebrate Richness, FNAIHAB, and Priority Natural Communities Core Data layers. For the TNC Recognized Biodiversity Value Analysis, we included only Priority 1 and 2 land (highest conservation priority). Credits: Florida State University - Florida Natural Areas Inventory, and University of Florida - Center for Landscape Conservation Planning. Credit: Florida Natural Areas Inventory, Florida State University (Jon Oetting) and Center for Landscape Conservation Planning, University of Florida (Tom Hoctor and Michael Volk). https://www.fnai.org/pdf/CLIP_v4_technical_report.pdf
GA	Georgia	Y	Georgia (2006). SWAP Priority Conservation Areas.	Georgia Dept. of Natural Resources Priority Conservation Areas 2006. In Georgia SWAP 2015 report.
ID	Idaho	N		
IL	Illinois	Y	Illinois (2016): COAs currently recognized through the Illinois Wildlife Action Plan (Figure 1).	Defined as "areas with significant existing or potential wildlife and habitat resources; places where partners are willing to plan, implement, and evaluate conservation actions; where financial and human resources are available, and where conservation is motivated by an agreed-upon conservation purpose and set of objectives" Centered on dataset of state's key blocks of habitat & the corridors that connect them. We removed polygons identified as rivers. https://www.dnr.illinois.gov/conservation/iwap/pages/default.aspx

IN	Indiana	Y	Indiana (2015): Indiana conservation opportunity areas (Figure 5-22).	COAs were designated based on SGCN distribution data, unique habitat communities, assessment of long term viability, current conservation actions and partnerships, threat assessment, and connectivity/potential to reconnect, and likelihood of obtaining funding. We used just the terrestrial polygons. https://www.in.gov/dnr/fishwild/7580.htm
IA	Iowa	Y	Iowa (2015): High Opportunity Areas for Cooperative Conservation Actions (Map 8-25).	This map sums the priorities from 22 terrestrial and aquatic assessments from field staff and many partners. Values range from 1-12, indicating the number of plans that highlighted each pixel. We selected areas that scored 4 or above (i.e. were identified in four or more of the component maps). The sources and methods are in Chapter 8. http://www.iowadnr.gov/Conservation/Iowas-Wildlife/Iowa-Wildlife-Action-Plan
KS	Kansas	Y	Kansas (2016): Terrestrial Ecological Focal Areas (Chapter 2, Figure 3B).	Designated “Ecological Focus Areas” – landscapes where conservation actions can be applied for maximum benefit to all Kansas wildlife (Ch. 2, p. 8). Each includes a suite of SCGN and priority habitats, and a “unique set of conservation actions designed to address the specific resource concerns facing these species and habitats.” Data layers include large natural areas & connectivity from the CHAT. https://ksoutdoors.com/Services/Kansas-SWAP
KY	Kentucky	N		
LA	Louisiana	Y	Louisiana (2019) Conservation Opportunity Areas.	LA Wildlife & Fisheries. Conservation Opportunity Areas COAS April 2019
ME	Maine	Y	Maine Focus Areas (2010)	Maine Department of Conservation, Maine Natural Areas Program
MD	Maryland	Y	Maryland (2016) Bionet	Maryland Biodiversity Conservation Network (Bionet). 2016 Tier 1-3 sites. Those sites described in Tiers as Critically (1), Extremely (2), Highly Significant (3) for biodiversity.
MA	Massachusetts	Y	Massachusetts (2010) BioMap2	Woolsey, H., et al. 2010. BioMap2: Conserving the Biodiversity of Massachusetts in a Changing World. MA Department of Fish and Game/Natural Heritage & Endangered Species Program and The Nature Conservancy/Massachusetts Program. 6 Feature types were extracted from BioMap2: Forest Cores, Priority Natural Communities, Species of Conservation Concern, Biomap2 Wetlands,Vernal Pool Core,and Landscape Blocks.
MI	Michigan	Y	Michigan: Biodiversity Stewardship areas	Not from the SWAP but recommended and shared by the SWAP coordinator as the most appropriate dataset for Michigan. Developed through an intensive statewide process to develop a map of high priority areas for protecting biodiversity approximately 10 years ago. Informed the current SWAP, but map not presented in the 2015 plan.

MN	Minnesota	Y	Minnesota (2015): The Wildlife Action Network map, terrestrial components (Fig 1.3)	The Wildlife Action Network incorporates SGCN populations and sites with high SGCN richness, as well as viability. It serves three purposes: 1) addresses large-scale habitat stressors such as climate change, fragmentation, and invasive species; 2) increase the efficiency of actions by the conservation community; 3) prioritize and focus conservation through an additional step of identifying Conservation Focus Areas (a prioritization for the next 10 years). https://www.dnr.state.mn.us/mnwap/index.html https://gisdata.mn.gov/dataset/env-mnwap-wildlife-action-netwrk
MS	Mississippi	Y	Mississippi (2015) Mississippi Conservation Opportunity Areas	Mississippi Conservation Opportunity Areas: Geospatial Data Presentation Form: vector digital data https://www.sciencebase.gov/catalog/item/5849874be4b06d80b7b094fa
MO	Missouri	Y	Missouri (2015): 2015 Conservation Opportunity Areas separated by habitat systems (Fig. 4)	In the MO SWAP, COAs were divided by type (grassland, forest, river, etc.) and each had a different set of scoring criteria. For grasslands, the criteria include a pre-settlement prairie layer, current land condition from NLCD, and community records from the Heritage Program database. We used just the terrestrial system COAs. https://mdc.mo.gov/sites/default/files/downloads/SWAPopt.pdf
MT	Montana	Y	Montana (2015): Tier 1 Terrestrial Focal Areas (Fig. 133)	The plan delineates habitat (plant communities) of most critical conservation need as well as SGCN, emphasizing SGCN with state ranks of S1 or S2. The plan notes differences in the process east and west of Continental Divide; the east focused more on intact landscapes, while teams in the west focused more on connectivity between protected areas. http://fwp.mt.gov/fishAndWildlife/conservationInAction/actionPlan.html
NE	Nebraska	Y	Nebraska (2015): Nebraska Natural Legacy Project: Biologically Unique Landscapes and Demonstration Sites. State Wildlife Action Plan.	Identified Biologically Unique Landscapes (BULs) – based on key habitats, Heritage Program data on locations of natural communities, and at-risk species. Incorporated a fine filter of Tier 1 and Tier 2 species; the list includes vertebrates, mollusks, insects, and plants (768 species). Incorporated Spatial Analysis Optimization Tool (SPOT) and Natural Heritage Program Hotspot analysis but did not attempt to capture corridors/connectivity. Map also includes Natural Legacy demonstration sites. We removed rivers and streams. http://outdoornebraska.gov/naturallegacyproject/
NV	Nevada	Y	Nevada (2017) Wildlife Action Plan.	Focal areas identified in the Nevada Wildlife Action Plan (2012) as discrete landscape units that provide a framework for evaluating the WAP in a statewide context. Feature Layer by cvandellen Created: Mar 13, 2017 Updated: Mar 13, 2017
NH	New Hampshire	N		

NJ	New Jersey	Y	New Jersey (2017). SWAP Conservation Focal Areas.	Conservation Focal Areas Version 1.0 All Landscape Regions (2017). State Wildlife Action Plan Dept of Enviornmantal Protection.
NM	New Mexico	Y	New Mexico (2016): Conservation Opportunity Areas (Fig. 11)	Defined as areas considered to have superior potential for conserving SGCN. Incorporates priority habitats from assessments with the New Mexico CHAT tool. This priority habitat layer was intersected with 5 other GIS layers, including SCGN point locations, species distribution model polygons for SCGN, large intact blocks from CHAT. The weighting scheme included availability of funding. Clusters with scores in the top 10% were selected as COAs. http://www.wildlife.state.nm.us/conservation/state-wildlife-action-plan/
NY	New York	Y	New York	June 2019 Update to TNC Portfolio in NY included polygon shapes for Portfolio Species and Community Element Occurrences obtained for NY Natural Heritage Program and Updated Matrix Forest Blocks revised by TNC. The Matrix Update was composed of 11 new or boundary revised blocks which now match the matrix blocks found in the "Biodiversity and Wind Energy Siting in New York" web map tool (2014) and the "Natural Resource Navigator" web map tool (2017).

NC	North Carolina	Y	North Carolina (2015) State Wildlife Action Plan	Theses NC SWAP Conservation Opportunity Area ShapeFiles were appended and included for the confirmed diversity layer/analysis. COASTAL PLAIN: Blackwater_Floodplains, Brownwater_Floodplains, Caves_Mines, Dry_LL_Pine_Forest, Estuarine_Wetlands, FW_Tidal_Wetlands, Low_Elev_Rocks, Maritime_Grasslands, Maritime_Upland_Forests, Maritime_Wetland_Forests, Mesic_Forests, Nonalluvial_Mineral_Wetlands, Pocosins, Upland_Pools_Depressions, Upland_Seeps_Spray_Cliffs, Wet_Pine_Savannas MOUNTAINS: Bogs_Fens, Caves_Mines, Cove_Forest, Dry_Coniferous_Woodlands, Grass_Heath_Balds, GW_Springs_Cavewaters_coldwater, High_Elev_Cliffs_Rocks, Inland_Floodplains, Low_Elev_Rocks, Mafic_Glades_Barrens, Montane_Oak_Forest, Northern_HW_Forest, Spruce_Fir_Forest, Upland_Pools_Depressions, Upland_Seepages_Spray_Cliffs PIEDMONT: Caves_Mines, Dry_Coniferous_Forest, Dry_LL_Pine_Forest, Low_Elev_Rocks, Mafic_Glades_Barrens, Mesic_Forests, Upland_Pools_Depressions, Upland_Seepages_Spray_Cliffs SANDHILLS: Brownwater_Floodplains, Caves_Mines, Dry_LL_Pines, Inland_Floodplains, Mesic_Forest, Nonalluvial_Mineral_Wetlands, Pocosins, Upland_Pools_Depressions, Wet_Pine_Savannas
ND	North Dakota	Y	North Dakota (2015): North Dakota State Wildlife Plan focal areas (Figure 7)	The plan notes that "focus areas typically exhibited unique or easily identifiable differences in vegetation, soils, topography, hydrology, or land use. Focal areas are highly variable in size and often represent an area of native vegetation or a natural community type rare to North Dakota." We removed the river and stream focal areas. https://gf.nd.gov/wildlife/swap
OH	Ohio	Y	Ohio (2015): COAs in individual maps – for example, Appalachian Foothills Forest COA (Fig 11).	A set COAs were developed by habitat type. "The idea is to concentrate efforts and resources to provide all the necessary habit requirements in a few, relatively large landscapes of major habitat types, along with the remnants of several unique habitats, for species that are of limited distribution or have low populations." COAs tend to connect nearby public lands/protected areas. We obtained a shapefile with all terrestrial COAs from the plan coordinator. http://wildlife.ohiodnr.gov/ohioswap
OK	Oklahoma	N	Oklahoma (2015): None.	Focus area delineation is in progress.

OR	Oregon	Y	Oregon Conservation Strategy. 2016. Oregon Department of Fish and Wildlife, Salem, Oregon	The delineation of the 2016 Conservation Opportunity Area boundaries was based upon a rigorous spatial analysis, using a conservation prioritization and spatial modeling program called Marxan. Marxan provided decision support for the design of conservation areas, using best available data to focus on concentrations of Strategy Species, Strategy Habitats, and additional datasets related to selected Key Conservation Issues.<br>The results of the spatial modeling analysis were reviewed by ODFW Fish and Wildlife Biologists as well as the Stakeholder Advisory Committee convened by the ODFW for the Conservation Strategy.<br>https://oregonconservationstrategy.org/media/kalins-pdf/COAs.pdf.
PA	Pennsylvania	Y	Pennsylvania (2011). Conservation Opportunity Areas.	Pennsylvania Conservation Opportunity Areas from https://www.sciencebase.gov/catalog/item/584991a4e4b06d80b7b0954b Terrestrial sites (freshwater sites removed). This layer displays Conservation Opportunity Areas (COA's), which are places in Pennsylvania that represent clusters of Species, as well as most critically imperiled plants and their associated habitats where collaborative conservation action should be targeted. The COAs are intended to complement, not replace, other conservation planning efforts, by providing specific recommendations focused on Species and their habitats. Credits Pennsylvania DCNR, 2011
RI	Rhode Island	Y	Rhode Island (2019). Natural Heritage Areas and TNC Portfolio Update	1. Natural Heritage Element Occurrence Concentration Areas<br>Citation: RIGIS, 2019. Rhode Island Natural Heritage Areas; natHeritage19. EO_concentrations. Rhode Island Geographic Information System (RIGIS) Data Distribution System, URL: http://www.rigis.org, Environmental Data Center, University of Rhode Island, Kingston, Rhode Island (last date accessed: 6 August 2019).<br>Description: The Natural Heritage Areas were developed from a kernel density analysis of Heritage data element occurrences (EO). These data are recognized by the State of Rhode Island as places supporting biodiversity. This layer was used for this purpose in the state's SWAP.<br>2. Recognized Biodiversity: The Nature Conservancy in Rhode Island's Whole System Portfolio. Citation: Kevin Ruddock, GIS Manager, The Nature Conservancy in Rhode Island. It identifies examples of common habitats (matrix forest) and complementary rare habitats (patch systems). of roadless blocks identified as the best opportunity to provide connectivity between the "Borderlands" matrix forest

SC	South Carolina	N		SWAP map covers most of the state so not precise enough to use.
SD	South Dakota	Y	South Dakota (2015): Map of terrestrial conservation opportunity areas (Fig. 6.6).	Terrestrial and aquatic COAs were proposed to encourage voluntary ecosystem restoration with an emphasis on the occurrence of SGCN and intact native habitats (101 SCGN were identified). Used NRCS Major Land Resource Areas as framework, then within each, attempted to meet the goal of maintaining more than or restoring at least 10% of primary historical ecological ecosystems for each ecological site type. Incorporated large intact blocks from CHAT model, species richness data & other sources. https://gfp.sd.gov/wildlife-action-plan/
TN	Tennessee	Y	Tennessee (2015) SWAP Terrestrial Habitat Priorities High and Very High.	Tennessee SWAP 2015. Terrestrial Habitat Priorities. Category 4 High and 5 Very High
TX	Texas	Y	Texas (2012, revising now):	Texas in in the process of revising their plan and has two types of assessments that were appropriate for this application, but only one was complete at the time of our compilation. We have incorporated an assessment a CHAT product, which incorporates SCGN distributions, but is primarily intended to identify sensitive resources and direct development away from them. This map draws information from an aggregated biodiversity value metric that is not yet complete for the state. The CHAT map uses these terrestrial maps as input, prioritizing areas that have confirmed presence and high-quality habitats. These “in progress” products were shared directly by the plan developers and are not in the current SWAP.
UT	Utah	N		
VT	Vermont	Y	Vermont (2019) Natural communities and species.	Natural communities and species. Vermont Natural Heritage Inventory, VT ANR, F&W. 2-27-2019. RTE and Significant Natural Communities at http://geodata.vermont.gov/datasets/VTANR::rte-and-significant-natural-communities; This is the most recent version (2/27/2019) of the RTE species and state significant natural communities available for the State of Vermont. The Vermont State Wildlife Action Plan (SWAP) adopts a coarse filter /fine filter strategy and relies upon this dataset for the fine filter component of the plan. As such, it is the best representation of field-verified biodiversity in the state. It is also intended to represent the natural community component of Vermont's ecoregional portfolio sites.
VA	Virginia	Y	Virgina (2018). Conserve Virginia	Conserve Virginia NatHabitat (2018). VaNLA Cores YES high priority Conservation Vision Ecological Cores are included NH Conservation Site YES- high priority Natural Heritage Conservation Sites are included
WA	Washington	N		

WV	West Virginia	N		Focus areas that covered most of the state in its SWAP so not precise enough to use.
WI	Wisconsin	Y	Wisconsin 2015: Wisconsin Conservation Opportunity Areas (multiple regional maps).	COAs were defined as places on the landscape that contain significant ecological features, natural communities, or SCGN habitat for which WI has responsibility. These were ranked by global, continental, Upper Midwest, and state priority. The report presents separate terrestrial and aquatic COAs. We incorporated all these levels. https://dnr.wi.gov/topic/wildlifehabitat/actionplan.html A compiled statewide map is here: https://dnr.wi.gov/topic/WildlifeHabitat/documents/MapCOA_statewide.pdf
WY	Wyoming	Y	Wyoming (2010): No map in the 2017 revision, but we incorporated SGCN priority areas from the 2010 plan.	Wyoming defined COAs in the 2010 SWAP based on a MARXAN analysis of priority habitats for SCGN for a suite of habitat types (input maps are shown in Figs 1-10 and 15 in the 2010 plan). This prioritization was not included in the 2017 SWAP revision, as stakeholders in Wyoming preferred access to input datasets on overlap in SCGN ranges, landscape intactness, etc., rather than the final prioritization product. We included this 2010 product but note that this is not a product that WY is currently using to guide implementation. Links to the 2017 and the 2010 plan: https://wgfd.wyo.gov/Habitat/Habitat-Plans/Wyoming-State-Wildlife-Action-Plan

D. Additional Sources

**Additional Recognized Biodiversity Value Areas**	**Title**	**Description**
GAP 1 and 2 Lands	Protected lands in GAP Status 1 or 2	This dataset included polygons from our PADV2-TNC augmented secured lands layer which represented areas of high biodiversity management and value. This included all GAP 1 and 2 Lands, including from National Park Service: National Parks and Wilderness Areas; USFS: Research Natural Areas, Wilderness, Proposed Wilderness, National Forest Roadless Areas; USFWS: Wilderness, National Wildlife Refuge; BLM: Wilderness areas, Research Natural Areas; specific National Monuments (selected for outstanding geodiversity), and The Nature Conservancy fee and easement lands. GAP1 have as their intent "Nature conservation" with little human interference and a mandated management plan in operation to maintain a natural state within which disturbance events can proceed without interference. GAP 2 lands have as their intent "Nature conservation", and allow hands-on management as needed.
Confirmed Biodiversity Sites - Eastern US	State Natural Heritage Species and Natural Community Element Occurrences from 22 Eastern US states. Used with permission.	A-C quality rare species locations and A-C quality community occurrences which which were not captured in the ecoregion or state based recognized biodiversity values. The analysis also included largest resilient patch of each geophysical setting if not already captured by the the ecoregion of state-based datasets, which restricted the actual additions to a few rare and underrepresented geophysical settings.
Confirmed Biodiversity Sites - Central US	State Natural Heritage Species and Natural Community Element Occurrences from Midwestern US states. Used with permission.	One highly converted geophysical setting (Clay/Silt in the Northern Tallgrass Prairie), was not represented in the ecoregion and state-based biodiversity plan . For this setting we identified some sites of confirmed biodiversity by overlaying the natural heritage element occurrences on the areas of above-average resilience and adding in contiguous patches of resilience on this setting if they contained an A or B ranked natural community.
Greater Sage-grouse Priority Areas for Conservation (PACs)	Sage-Grouse Conservation Objectives Team (COT) 2013, USFWS. https://my.usgs.gov/arcgis/rest/services/Catalog/555a2939e4b0a92fa7ea13f6/MapServer/0	This polygon data set represents all sage-grouse Priority Areas for Conservation (PACs) identified in the 2013 Greater Sage-Grouse Conservation Objectives Team (COT) Report. PACs represent areas identified as essential for the long-term conservation of the sage-grouse. The COT determined that the PACs are key for the conservation of the species range wide.

Table S6. Secured areas data sources.

The secured areas dataset shows public and private lands that are permanently secured against conversion to development through fee ownership, easements, or permanent conservation restrictions. The dataset is a mix of federal, state, and local data sources compiled by a variety of agencies. The dataset and source for each polygon is available via the interactive map on the authoritative data page: https://tnc.maps.arcgis.com/home/item.html?id=e033e6bf6069459592903a04797b8b07

**National Sources**
Protected Areas Database of the U.S. (PAD-US 2.1, 2, and 1). U.S. Geological Survey (USGS) Gap Analysis Project (GAP).
National Conservation Easement Database (NCED). Ducks Unlimited and Trust for Public Land.
TNC Lands. The Nature Conservancy. Boundaries of TNC owned and managed land.
Canadian Protected and Conserved Areas Database (CPCAD) 2020, Canadian Council on Ecological Areas (CARTS)
**Regional Sources**
Eastern U.S. Secured Areas. The Nature Conservancy (TNC), State Chapter GIS compilations and contributions covering 22 Eastern US states and Eastern Canada.
Conservation And Recreation Lands (CARL) in the Great Lakes Atlantic Region. Ducks Unlimited
**State Sources**
California Protected Lands Database (CPAD)
California Conservation Easement Database (CCED)
Illinois Protected Natural Lands,(I-view) Prairie State Conservation Coalition
Indiana Managed Lands. Indiana Dept. of Natural Resources
Public Lands for Conservation and Recreation in IOWA
Minnesota Dept. of Natural Resources: State Managed Public Lands

Table S7. Distribution and Securement of the Resilient and Connected Network. The table is organized by Region (West, Midwest, East) and Ecoregions (north to south). RFB = Resilience Flow and Biodiversity, RF = Resilience and Flow, sRB = secured Resilience and Biodiversity, sR = secured Resilient. GAP 1-2 = land permanently protected for biodiversity, GAP 3 = Land permanently secured for multiple uses. Tribal = Federally recognized tribal land.

**Ecoregion**		**RCN**					**% Securement RCN**			
Name	Acres	RFB	RF	sRB	sR	Total	GAP 1-2	GAP 3	Tribal	Total
Okanagan	5,729,072	57%	24%	1%	0%	82%	25%	31%	18%	74%
North Cascades	3,254,514	69%	8%	2%	0%	79%	74%	20%	0%	94%
West Cascades	10,637,880	54%	11%	2%	0%	67%	36%	51%	1%	89%
Middle Rockies - Blue Mountains	52,195,251	35%	23%	0%	0%	59%	55%	27%	0%	82%
Klamath Mountains	12,122,902	38%	15%	1%	0%	55%	40%	43%	0%	84%
Canadian Rocky Mountains	21,214,330	39%	11%	1%	0%	52%	57%	29%	3%	88%
Columbia Plateau	73,024,647	32%	18%	1%	0%	52%	19%	47%	2%	68%
Pacific Northwest Coast	10,741,830	35%	14%	1%	0%	50%	24%	41%	2%	67%
East Cascades - Modoc Plateau	16,758,972	29%	13%	1%	0%	43%	33%	47%	2%	82%
Willamette Valley - Puget Trough	9,413,643	18%	7%	0%	0%	25%	4%	14%	0%	19%
**Pacific Northwest Avg.**	**21,509,304**	**41%**	**14%**	**1%**	**0%**	**56%**	**37%**	**35%**	**3%**	**75%**
Sierra Nevada	12,347,296	68%	5%	2%	0%	75%	52%	35%	0%	87%
California North Coast	7,147,608	61%	5%	1%	0%	68%	17%	21%	1%	39%
California Central Coast	11,798,880	55%	9%	1%	0%	65%	21%	10%	0%	31%
California South Coast	9,340,226	49%	1%	3%	0%	53%	50%	25%	2%	77%
Great Central Valley	18,675,465	28%	8%	2%	0%	37%	16%	8%	0%	25%
**California Avg.**	**11,861,895**	**52%**	**6%**	**2%**	**0%**	**60%**	**31%**	**20%**	**1%**	**52%**
Utah-Wyoming Rocky Mountains	27,054,026	47%	5%	9%	1%	62%	67%	21%	3%	91%
Southern Rocky Mountains	39,928,155	35%	17%	5%	2%	59%	36%	38%	2%	75%
Utah High Plateaus	11,342,207	27%	16%	10%	5%	59%	31%	50%	4%	85%
Arizona-New Mexico Mountains	28,788,014	20%	20%	6%	3%	50%	22%	41%	8%	70%

Great Basin	72,408,220	25%	13%	8%	4%	49%	24%	65%	1%	89%
Colorado Plateau	48,553,547	21%	17%	4%	2%	44%	32%	35%	12%	79%
Wyoming Basins	33,023,264	26%	9%	6%	2%	44%	9%	59%	3%	71%
**Rocky Mountains Avg.**	**37,299,633**	**29%**	**14%**	**7%**	**3%**	**52%**	**31%**	**44%**	**5%**	**80%**
Apache Highlands	20,642,266	35%	23%	3%	1%	61%	20%	39%	10%	70%
Mojave Desert	32,274,829	43%	1%	6%	0%	50%	64%	18%	0%	82%
Sonoran Desert	28,658,578	23%	13%	4%	2%	42%	37%	31%	5%	72%
Chihuahuan Desert	38,573,739	20%	18%	1%	1%	40%	11%	16%	0%	26%
Tamaulipan Thorn Scrub	19,644,731	11%	8%	1%	0%	19%	13%	1%	0%	14%
**Warm Deserts/ Tamaulipan Avg.**	**27,958,828**	**26%**	**13%**	**3%**	**1%**	**43%**	**29%**	**21%**	**3%**	**53%**
Black Hills	3,277,203	51%	13%	0%	0%	64%	1%	11%	0%	12%
Fescue-Mixed Grass Prairie	3,645,860	45%	3%	1%	0%	49%	5%	11%	24%	40%
Northern Great Plains Steppe	123,648,080	26%	16%	0%	0%	43%	4%	20%	6%	30%
Edwards Plateau	23,495,864	28%	13%	0%	0%	41%	3%	1%	0%	4%
Crosstimbers /S. Tallgrass Prairie	49,093,591	14%	24%	0%	0%	38%	2%	1%	0%	3%
Southern Shortgrass Prairie	68,901,538	19%	19%	0%	0%	38%	1%	5%	0%	6%
Osage Plains/Flint Hills Prairie	19,786,397	24%	12%	0%	0%	37%	4%	0%	0%	5%
Central Mixed-Grass Prairie	59,104,439	28%	8%	0%	0%	36%	2%	1%	1%	4%
Central Shortgrass Prairie	55,701,748	25%	8%	0%	0%	33%	4%	11%	0%	15%
**Great Plains Avg.**	**45,183,858**	**29%**	**13%**	**0%**	**0%**	**42%**	**3%**	**7%**	**3%**	**13%**

Ecoregion		RCN					% Securement RCN			
Name	Acres	RFB	RF	sRB	sR	Total	GAP 1-2	GAP 3	Tribal	Total
Superior Mixed Forest	39,029,181	29%	10%	1%	0%	40%	21%	32%	2%	55%
Dakota Mixed-Grass Prairie	26,916,089	13%	4%	0%	0%	18%	5%	6%	2%	13%
Great Lakes	84,571,779	7%	6%	0%	0%	14%	20%	22%	0%	43%
Prairie-Forest Border	39,197,593	7%	3%	1%	0%	12%	12%	20%	0%	31%
Aspen Parkland	3,720,243	7%	3%	1%	0%	11%	21%	8%	1%	30%
Central Tallgrass Prairie	70,640,023	4%	5%	0%	0%	10%	7%	8%	0%	15%
Northern Tallgrass Prairie	42,376,970	4%	1%	1%	0%	7%	17%	24%	1%	41%
North Central Tillplain	30,472,964	2%	2%	0%	0%	5%	10%	20%	0%	29%
**Great Lakes/Tallgrass Prairie**	**42,115,605**	**9%**	**4%**	**1%**	**0%**	**14%**	**14%**	**17%**	**1%**	**32%**
Ozarks	34,342,106	18%	28%	0%	0%	46%	9%	14%	0%	23%
Ouachita Mountains	11,482,224	21%	20%	0%	0%	42%	35%	8%	0%	43%
West Gulf Coastal Plain	10,857,960	16%	11%	1%	0%	28%	15%	9%	0%	24%
Upper West Gulf Coastal Plain	26,045,292	8%	17%	0%	0%	26%	5%	5%	0%	10%
Mississippi River Alluvial Plain	27,060,839	8%	8%	3%	0%	19%	29%	10%	0%	39%
Gulf Coast Prairies and Marshes	18,980,727	2%	14%	0%	0%	17%	13%	3%	0%	16%
**Lower Mississippi and Ozarks**	**21,461,524**	**12%**	**17%**	**1%**	**0%**	**30%**	**18%**	**8%**	**0%**	**26%**
Northern Appalachian / Acadian	31,550,484	46%	21%	0%	0%	67%	21%	21%	0%	42%
Central Appalachian Forest	23,881,281	31%	6%	2%	0%	39%	13%	39%	0%	52%
High Allegheny Plateau	16,892,503	22%	13%	1%	1%	37%	10%	42%	0%	52%
St. Lawrence - Champlain Valley	4,037,883	18%	9%	1%	0%	28%	2%	20%	0%	22%
Lower New England/N. Piedmont	23,223,294	14%	5%	2%	1%	22%	10%	28%	0%	38%
Western Allegheny Plateau	26,673,076	4%	6%	1%	0%	11%	12%	17%	0%	29%
North Atlantic Coast	7,778,915	3%	3%	2%	0%	9%	29%	34%	0%	63%
Chesapeake Bay Lowlands	8,040,622	1%	5%	1%	0%	8%	13%	35%	0%	48%

**Northeast**	**17,759,757**	**17%**	**9%**	**1%**	**0%**	**28%**	**14%**	**29%**	**0%**	**43%**
Southern Blue Ridge	9,413,594	46%	2%	3%	0%	51%	23%	48%	0%	70%
Cumberlands/ S. Ridge & Valley	31,055,211	25%	14%	1%	0%	39%	5%	15%	0%	19%
Tropical Florida	9,305,433	12%	13%	1%	0%	26%	54%	29%	0%	83%
Florida Peninsula	14,791,069	17%	4%	3%	0%	23%	10%	46%	0%	56%
East Gulf Coastal Plain	42,005,669	12%	8%	1%	0%	20%	8%	24%	0%	32%
Upper East Gulf Coastal Plain	33,862,242	10%	9%	0%	0%	19%	6%	11%	0%	17%
Mid-Atlantic Coastal Plain	23,983,519	10%	7%	1%	0%	18%	15%	16%	0%	31%
South Atlantic Coastal Plain	23,548,663	9%	6%	1%	0%	17%	20%	14%	0%	35%
Interior Low Plateau	47,786,675	8%	7%	0%	0%	15%	11%	8%	0%	18%
Piedmont	42,345,785	4%	3%	1%	1%	9%	9%	27%	0%	36%
**Southeast**	**27,809,786**	**15%**	**7%**	**1%**	**0%**	**24%**	**16%**	**24%**	**0%**	**40%**
**TOTAL**	**1,967,818,721**	21%	12%	2%	<1%	35%	21%	23%	2%	46%

Table S8. Example of TNC Ecoregional Plan Conservation Targets List: Central Appalachian Ecoregion: APPENDIX II—List of Conservation Targets

1. Vertebrates

GNAME	GCOMNAME	GRANK
NEOTOMA MAGISTER	ALLEGHENY WOODRAT	G3G4
MYOTIS LEIBII	EASTERN SMALL-FOOTED MYOTIS	G3
GLAUCOMYS SABRINUS FUSCUS	VIRGINIA NORTHERN FLYING SQUIRREL	G5T2
CORYNORHINUS TOWNSENDII VIRGINIANUS	VIRGINIA BIG-EARED BAT	G4T2
ANEIDES AENEUS	GREEN SALAMANDER	G3G4
PLETHODON PUNCTATUS	WHITE-SPOTTED SALAMANDER	G3
MYOTIS SODALIS	INDIANA OR SOCIAL MYOTIS	G2
PLETHODON NETTINGI	CHEAT MOUNTAIN SALAMANDER	G2
PERCINA REX	ROANOKE LOGPERCH	G2
PLETHODON SHENANDOAH	SHENANDOAH SALAMANDER	G1
NOTURUS GILBERTI	ORANGEFIN MADTOM	G2
NOTROPIS SEMPERASPER	ROUGHHEAD SHINER	G2G3
RHINICHTHYS BOWERSI	CHEAT MINNOW	G1G2
SCARTOMYZON ARIOMMUS	BIGEYE JUMPROCK	G2
ETHEOSTOMA OSBURNI	CANDY DARTER	G3
CLEMMYS MUHLENBERGII	BOG TURTLE	G3
PLETHODON HUBRICHTI	PEAKS OF OTTER SALAMANDER	G2
THRYOMANES BEWICKII ALTUS	APPALACHIAN BEWICK'S WREN	G5T2Q
AMBLOPLITES CAVIFRONS	ROANOKE BASS	G3
GYRINOPHILUS SUBTERRANEUS	WEST VIRGINIA SPRING SALAMANDER	G1Q
SOREX PALUSTRIS PUNCTULATUS	SOUTHERN WATER SHREW	G5T3
MICROTUS CHROTORRHINUS CAROLINENSI	SOUTHERN ROCK VOLE	G4T3

2. Plants

GNAME	GCOMNAME	GRANK
PTILIMNIUM NODOSUM	HARPERELLA	G2
ILEX COLLINA	LONG-STALKED HOLLY	G3
ECHINACEA LAEVIGATA	SMOOTH CONEFLOWER	G2
HELENIUM VIRGINICUM	VIRGINIA SNEEZEWEED	G2
LIATRIS TURGIDA	TURGID GAY-FEATHER	G3
MARSHALLIA GRANDIFLORA	LARGE-FLOWERED BARBARA'S-BUTTONS	G2
PRENANTHES CREPIDINEA	NODDING RATTLESNAKE-ROOT	G3G4
RUDBECKIA TRILOBA VAR PINNATILOBA	PINNATE-LOBED BLACK-EYED SUSAN	G4T2?
SYNOSMA SUAVEOLENS	SWEET-SCENTED INDIAN-PLANTAIN	G3G4
ARABIS PATENS	SPREADING ROCKCRESS	G3
ARABIS SEROTINA	SHALE-BARREN ROCKCRESS	G2
CARDAMINE FLAGELLIFERA	BITTER CRESS	G3
PARONYCHIA VIRGINICA VAR VIRGINICA	YELLOW NAILWORT	G4T1T2
SILENE VIRGINICA VAR ROBUSTA		G5T1Q
PAXISTIMA CANBYI	CANBY'S MOUNTAIN-LOVER	G2
HYPERICUM MITCHELLIANUM	BLUE RIDGE ST. JOHN'S-WORT	G3
GAYLUSSACIA BRACHYCERA	BOX HUCKLEBERRY	G2G3
EUPHORBIA PURPUREA	GLADE SPURGE	G3
TRIFOLIUM STOLONIFERUM	RUNNING BUFFALO CLOVER	G3
TRIFOLIUM VIRGINICUM	KATE'S-MOUNTAIN CLOVER	G3
MONARDA FISTULOSA SSP 1	SMOKE HOLE BERGAMOT	G5T1
PYCNANTHEMUM TORREI	TORREY'S MOUNTAIN MINT	G2
STACHYS CLINGMANII	CLINGMAN'S HEDGE-NETTLE	G2
ILIAMNA REMOTA	KANKAKEE GLOBE-MALLOW	G1Q
ILIAMNA COREI	PETERS MOUNTAIN MALLOW	G1Q
SIDA HERMAPHRODITA	VIRGINIA MALLOW	G2
PHLOX BUCKLEYI	SWORD-LEAVED PHLOX	G2
POLEMONIUM VANBRUNTIAE	JACOB'S LADDER	G3
ACONITUM RECLINATUM	WHITE MONKSHOOD	G3

CLEMATIS ADDISONII	ADDISON'S LEATHERFLOWER	G2
CLEMATIS COACTILIS	VIRGINIA WHITE-HAIR LEATHER-FLOWER	G2G3
CLEMATIS VITICAULIS	MILLBORO LEATHERFLOWER	G1
DELPHINIUM EXALTATUM	TALL LARKSPUR	G3
SPIRAEA VIRGINIANA	VIRGINIA SPIRAEA	G2
BUCKLEYA DISTICHOPHYLLA	PIRATEBUSH	G2
HEUCHERA ALBA	WHITE ALUMROOT	G2Q
PARNASSIA GRANDIFOLIA	LARGE-LEAVED GRASS-OF-PARNASSUS	G3
VIOLA APPALACHIENSIS	APPALACHIAN BLUE VIOLET	G3
VITIS RUPESTRIS	ROCK GRAPE	G3
CAREX LUPULIFORMIS	FALSE HOP SEDGE	G3G4
CAREX POLYMORPHA	VARIABLE SEDGE	G3
CAREX SCHWEINITZII	SCHWEINITZ'S SEDGE	G3
SCIRPUS ANCISTROCHAETUS	NORTHEASTERN BULRUSH	G3
ALLIUM OXYPHILUM	LILLYDALE ONION	G2G3Q
CLINTONIA ALLEGHANIENSIS	HARNED'S CLINTONIA	G1Q
HELONIAS BULLATA	SWAMP-PINK	G3
TRILLIUM PUSILLUM	LEAST TRILLIUM	G3
TRILLIUM PUSILLUM VAR VIRGINIANUM	VIRGINIA LEAST TRILLIUM	G3T2
CLEISTES BIFARIA	SPREADING POGONIA	G3G4
ISOTRIA MEDEOLOIDES	SMALL WHORLED POGONIA	G2G3
PLATANTHERA LEUCOPHAEA	EASTERN PRAIRIE WHITE-FRINGED ORCHID	G2
POA LANGUIDA	DROOPING BLUEGRASS	G3G4Q
POA PALUDIGENA	BOG BLUEGRASS	G3
POTAMOGETON HILLII	HILL'S PONDWEED	G3
POTAMOGETON TENNESSEENSIS	TENNESSEE PONDWEED	G2
CYSTOPTERIS LAURENTIANA	LAURENTIAN BLADDER FERN	G3G4
GYMNOCARPIUM APPALACHIANUM	APPALACHIAN OAK FERN	G3
LYCOPODIELLA MARGUERITIAE	NORTHERN PROSTRATE CLUBMOSS	G2
STREPTOPUS AMPLEXIFOLIUS	CLASPING TWISTED-STALK	G4G5

ARETHUSA BULBOSA	SWAMP-PINK	G4G5
VACCINIUM OXYCOCCUS	SMALL CRANBERRY	G4G5
ASTRAGALUS DISTORTUS	OZARK MILK-VETCH	G4G5
LARIX LARICINA	AMERICAN LARCH	G4G5
CALLA PALUSTRIS	WILD CALLA	G4G5
CYPERUS HOUGHTONII	HOUGHTON'S UMBRELLA-SEDGE	G4G5
CYPERIPEDIUM CANDIDUM	SMALL WHITE LADY'S SLIPPER	G4G5
ERYSIMUM CAPITATUM	WESTERN WALLFLOWER	G4G5
HUDSONIA TOMENTOSA	SAND HEATHER	G4G5
ANDROMEDA POLIFOLIA GLAUCOPHYLL	BOG ROSEMARY	G5T5
CAREX PAUCIFLORA	FEW-FLOWERED SEDGE	G4G5
JUNCUS FILIFORMIS	THREAD RUSH	G4G5
JUNCUS TRIFIDUS	HIGHLAND RUSH	G4G5
AGROSTIS MERTENSII	ARCTIC BENTGRASS	G5
ORYZOPSIS CANADENSIS	CANADA MOUNTAIN-RICEGRASS	G4G5
CHIELANTHES EATONII	EATON LIPFERN	G5?
CRYPTOGRAMMA STELLERI	FRAGILE ROCKBRAKE	G4G5
ASPLENIUM SEPTENTRIONALE	NORTHERN SPLEENWORT	G4G5

Secondary Targets:

CAREX COLLINSII	COLLINS' SEDGE	G4G5
ABIES BALSAMEA	BALSAM FIR	G5
JUNIPERUS COMMUNIS	OLD-FIELD JUNIPER	G5
TAXUS CANADENSIS	CANADIAN YEW	G5

3. Invertebrates

GNAME	GCOMNAME	GRANK
TRICHODRILUS CULVERI		G1G2
STYLODRILUS BEATTIEI	A CAVE LUMBRICULID WORM	G1G2
CAECIDOTEA PRICEI	PRICE'S CAVE ISOPOD	G3
CAECIDOTEA FRANZI	FRANZ'S CAVE ISOPOD	G1
CAECIDOTEA HOLSINGERI	HOLSINGER'S CAVE ISOPOD	G3
CAECIDOTEA HENROTI	HENROT'S CAVE ISOPOD	G2
CAECIDOTEA CANNULUS	AN ISOPOD	G2
CAECIDOTEA SIMONINI		G1
CAECIDOTEA SP 1	ROCK SPRINGS CAVE ISOPOD	G1
CAECIDOTEA SP 3	JOHN FRIEND'S CAVE ISOPOD (MD)	G3
ANTROLANA LIRA	MADISON CAVE ISOPOD	G1
STYGOBROMUS BIGGERSI	BIGGERS' CAVE AMPHIPOD	G1G2
STYGOBROMUS GRACILIPES	SHENANDOAH VALLEY CAVE AMPHIPOD	G2
STYGOBROMUS PIZZINII	PIZZINI'S CAVE AMPHIPOD	G2
STYGOBROMUS FRANZI	FRANZ'S CAVE AMPHIPOD	G2
STYGOBROMUS EMARGINATUS	GREENBRIER CAVE AMPHIPOD	G3
STYGOBROMUS MORRISONI	MORRISON'S CAVE AMPHIPOD	G2
STYGOBROMUS STEGERORUM	MADISON CAVE AMPHIPOD	G1
STYGOBROMUS ABDITUS	JAMES CAVE AMPHIPOD	G1
STYGOBROMUS BAROODYI	ROCKBRIDGE COUNTY CAVE AMPHIPOD	G2
STYGOBROMUS CONRADI	BURNSVILLE COVE CAVE AMPHIPOD	G1G2
STYGOBROMUS ESTESI	CRAIG COUNTY CAVE AMPHIPOD	G1
STYGOBROMUS SPINOSUS	BLUE RIDGE MOUNTAIN AMPHIPOD	G2G3
STYGOBROMUS STELLMACKI	STELLMACK'S CAVE AMPHIPOD	G1
STYGOBROMUS SPINATUS	SPRING CAVE AMPHIPOD	G3
STYGOBROMUS PARVUS	MINUTE CAVE AMPHIPOD	G1
STYGOBROMUS REDACTUS	AN AMPHIPOD	G1
STYGOBROMUS CULVERI		G1
CRANGONYX DEAROLFI	PENNSYLVANIA CAVE AMPHIPOD	G1G2

CAMBARUS NERTERIUS	A CRAYFISH	G2
CAMBARUS ELKENSIS	ELK RIVER CRAYFISH	G2
MIKTONISCUS RACOVITZAE	RACOVITZA'S TERRESTRIAL CAVE ISOPOD	G2
SINELLA AGNA		G2
CICINDELA ANCOCISCONENSIS	A TIGER BEETLE	G3
CICINDELA PATRUELA	A TIGER BEETLE	G3
PSEUDANOPHTHALMUS LALLEMANTI	CAVE BEETLE	G1
PSEUDANOPHTHALMUS GRANDIS	A CAVE BEETLE	G3
PSEUDANOPHTHALMUS GRANDIS GRANDIS	A CAVE BEETLE	G3T3
PSEUDANOPHTHALMUS HYPERTRICHOSIS	A CAVE BEETLE	G3
PSEUDANOPHTHALMUS FUSCUS		G2
PSEUDANOPHTHALMUS POTOMACA POTOMACA	SOUTH BRANCH VALLEY CAVE BEETLE	G1T1
PSEUDANOPHTHALMUS AVERNUS	AVERNUS CAVE BEETLE	G1
PSEUDANOPHTHALMUS EGBERTI	NEW RIVER VALLEY CAVE BEETLE	G1
PSEUDANOPHTHALMUS HORTULANUS	GARDEN CAVE BEETLE	G1
PSEUDANOPHTHALMUS HUBBARDI	HUBBARD'S CAVE BEETLE	G1
PSEUDANOPHTHALMUS INTERSECTUS	CROSSROADS CAVE BEETLE	G1
PSEUDANOPHTHALMUS LIMICOLA	MUD-DWELLING CAVE BEETLE	G1
PSEUDANOPHTHALMUS NELSONI	NELSON'S CAVE BEETLE	G1
PSEUDANOPHTHALMUS PARVICOLLIS	THIN-NECK CAVE BEETLE	G1
PSEUDANOPHTHALMUS PETRUNKEVITCHI	PETRUNKEVITCH'S CAVE BEETLE	G1
PSEUDANOPHTHALMUS PONTIS	NATURAL BRIDGE CAVE BEETLE	G1
PSEUDANOPHTHALMUS PUNCTATUS	SPOTTED CAVE BEETLE	G1
PSEUDANOPHTHALMUS QUADRATUS	STRALEY'S CAVE BEETLE	G1
PSEUDANOPHTHALMUS HOFFMANI	A GROUND BEETLE	G1G2
PSEUDANOPHTHALMUS PUSIO	A GROUND BEETLE	G1?
PSEUDANOPHTHALMUS GRACILIS	A GROUND BEETLE	G1
PSEUDANOPHTHALMUS SP 6	A GROUND BEETLE	G1
PSEUDANOPHTHALMUS SP 7	A GROUND BEETLE	G1

PSEUDANOPHTHALMUS SP 8	A GROUND BEETLE (HUBBARDI GROUP)	G1
PSEUDANOPHTHALMUS SP 11	(PUSIO GROUP)	G1
PSEUDANOPHTHALMUS SP 15	MARYLAND CAVE BEETLE	G1
PYRGUS WYANDOT	SOUTHERN GRIZZLED SKIPPER	G2
SATYRIUM KINGI	KING'S HAIRSTREAK	G3G4
INCISALIA IRUS	FROSTED ELFIN	G3G4
SPEYERIA DIANA	DIANA	G3
SPEYERIA IDALIA	REGAL FRITILLARY	G3
MEROLONCHE DOLLI	DOLL'S MEROLONCHE	G3
PAPAIPEMA SP 1	FLYPOISON BORER MOTH	G2
PROPERIGEA SP 1	A NOCTUID MOTH	G2G3Q
CHAETAGLAEA CERATA	A NOCTUID MOTH	G3G4
GOMPHUS ABBREVIATUS	SPINE-CROWNED CLUBTAIL	G3G4
GOMPHUS VIRIDIFRONS	GREEN-FACED CLUBTAIL	G3
LANTHUS PARVULUS	NORTHERN PYGMY CLUBTAIL	G3G4
AESHNA MUTATA	SPATTERDOCK DARNER	G3G4
APOCHTHONIUS COECUS	A PSEUDOSCORPION	G1
KLEPTOCHTHONIUS HENROTI	GREENBRIER VALLEY CAVE PSEUDOSCORPION	G1
KLEPTOCHTHONIUS ANOPHTHALMUS	A PSEUDOSCORPION	G1
KLEPTOCHTHONIUS SP 1	A PSEUDOSCORPION	G1
CHITRELLA SUPERBA	A PSEUDOSCORPION	G1
MUNDOCHTHONIUS HOLSINGERI		G1
ALASMIDONTA VARICOSA	BROOK FLOATER	G3
ELLIPTIO LANCEOLATA	YELLOW LANCE	G2G3
FUSCONAIA MASONI	ATLANTIC PIGTOE	G2
LAMPSILIS CARIOSA	YELLOW LAMPMUSSEL	G3G4
LASMIGONA HOLSTONIA	TENNESSEE HEELSPLITTER	G2G3
LASMIGONA SUBVIRIDIS	GREEN FLOATER	G3
PLEUROBEMA COLLINA	JAMES SPINYMUSSEL	G1
POLYGYRISCUS VIRGINICUS	VIRGINIA COIL	G1
TRIODOPSIS PLATYSAYOIDES	CHEAT THREETOOTH	G1

FONTIGENS OROLIBAS	BLUE RIDGE SPRINGSNAIL	G3
FONTIGENS TARTAREA	ORGAN CAVESNAIL	G2
FONTIGENS BOTTIMERI	APPALACHIAN SPRINGSNAIL	G3
PROCOTYLA TYPHLOPS	A PLANARIAN	G1G2
SPHALLOPLANA PRICEI	REFTON CAVE PLANARIAN	G1
MACROCOTYLA HOFFMASTERI	HOFFMASTER'S CAVE PLANARIAN	G3
BUOTUS CAROLINUS	A MILLIPEDE	G1
DIXIORIA FOWLERI	A MILLIPEDE	G2
SEMIONELLUS PLACIDUS	A MILLIPEDE	G3
LYCAENA EPIXANTHES	BOG COPPER	G4G5
TRIODOPSIS PICEA	SPRUCE KNOB THREE-TOOTH	G3
LEUCORRHINA HUDSONICA	HUDSONIAN WHITEFACE	G5
HELICODISCUS DIADEMA	SHAGGY COIL	G1
HELICODISCUS LIRELLUS	RUBBLE COIL	G1

Secondary Targets:

ANTHROBIA MONMOUTHIA		G3G4
CALEPHELIS BOREALIS	NORTHERN METALMARK	G3G4
CALOPTERYX AMATA	SUPERB JEWELWING	G3G4
ERYNNIS PERSIUS PERSIUS	PERSIUS DUSKY WING	G4T2T3
OPHIOGOMPHUS ALLEGHANIENSIS	ALLEGHENY SNAKETAIL	G3Q
PHANETTA SUBTERRANEA		G3
PSEUDOSINELLA GISINI		G3

PSEUDOTREMIA ALECTO	A MILLIPEDE	G1
PSEUDOTREMIA FULGIDA	GREENBRIER VALLEY CAVE MILLIPEDE	G2
PSEUDOTREMIA PRINCEPS	SOUTH BRANCH VALLEY CAVE MILLIPEDE	G1
PSEUDOTREMIA SUBLEVIS	A MILLIPEDE	G1
STYGOBROMUS SP 7	SHERANDO SPINOSID AMPHIPOD	G2
STYLURUS SCUDDERI	ZEBRA CLUBTAIL	G3G4
TRICHOPETALUM PACKARDI	PACKARD'S BLIND CAVE MILLIPEDE	G3Q
TRICHOPETALUM WEYERIENSIS	GRAND CAVERNS BLIND CAVE MILLIPEDE	G3Q

4. Terrestrial and Palustrine Plant Communities

ECOGROUP	Descriptive name
MARSH	Baltic rush-tussock sedge marsh
CONIFER FOREST: MID/LOW ELEVATION:RIDGES & SLOPES	Shortleaf pine/heath forest of dry, acidic steep slopes
CONIFER FOREST: MID/LOW ELEVATION:RIDGES & SLOPES	Carolina hemlock forest
MIXED FOREST: MID/LOW ELEVATION: HEMLOCK	Eastern hemlock-yellow birch-black cherry forest
DECIDUOUS FOREST: MESIC: LOW SLOPES & COVES	Sugar maple-white ash-basswood-bluebead cove forest
RIVERSHORE: GRASSLAND	Torturous sedge gravel rivershore
AQUATIC: LAKE/POND	Mud plantain muddy ponds
WETLAND SHRUB THICKET	Smooth alder shrub thicket
CONIFER SWAMP: MID/LOW ELEVATION	Eastern hemlock-great laurel swamp
WETLAND SHRUB THICKET	Meadowsweet-dewberry shrub swamp
MARSH	Baltic rush-prairie sedge marsh
BARREN: GREENSTONE	White ash - Shagbark hickory woodlands
WETLAND SHRUB THICKET	Buttonbush semipermanantly flooded shrub swamp
BARREN: CALCAREOUS	Little bluestem calcareous grassy opening
MIXED FOREST: MID/LOW ELEVATION	Eastern hemlock-tuliptree forest
SHRUB SUMMIT:MID/LOW ELEVATION	Scrub oak summits
CONIFER FOREST: MID/LOW: MIDSLOPE: HEMLOCK-PINE	White pine-eastern hemlock dry forest:northern type
BARREN: PITCH PINE	Pitch pine/black chokeberry low-mid elevation ridgetop
BARREN: PITCH PINE	Pitch pine/scrub oak/black chokeberry low-mid elevation Ridgetop
MIXED FOREST: MID/LOW ELEVATION: HEMLOCK	Eastern hemlock-tuliptree-great laurel forest
MIXED FOREST: MID/LOW ELEVATION	Mixed pine-chestnut oak xeric forest (large patch to matrix)
DECIDUOUS FOREST: MESIC: N. HARDWOOD	Maple-Beech-Birch-Cherry northern hardwoods (matrix)
	Saxifrage-stonecrop rocky summit
SUMMIT: GRASS BALD	High elevation sparse summit

RIVERSHORE: SHRUB THICKET	Alder-ninebark thickets
DECIDUOUS FOREST: HIGH ELEVATION	High elevation red oak/blueberry-flame azalea forest
RIVERSHORE: SHRUB THICKET	River birch-willow thickets
SHRUB SUMMIT:HIGH ELEVATION	Mountain laurel-black huckleberry summit
SEEP: ACIDIC	Jewelweed-beebalm-coneflower seep
CLIFF: CALCAREOUS	Spleenwort-cliffbrake calcarous cliff
DECIDUOUS FOREST:MID/LOW XERIC:SLOPE:ALKALINE	Yellow oak-sugar maple-red bud forest of calcareous upper slopes and summits
BARREN: TALUS SLOPE	White ash-Basswood-dogwood alkaline talus slope
SWAMP: DECIDUOUS	Red maple-black gum swamp
BARREN: CALCAREOUS	Chinquapin oak-redbud calcareous woodland (northern type?)
SWAMP: DECIDUOUS	Pin oak-swamp white oak swamp
CONIFER SWAMP: MID/HIGH	Red spruce-hemlock/great laurel swamp
RIVERSHORE: GRASSLAND	Big bluestem-wild indigo riverside prairie
WOODED FEN	Red maple wooded fen
SWAMP: DECIDUOUS	Red maple-black ash swamp
MIXED SWAMP: MID/HIGH	Eastern hemlock-red maple-great laurel swamp
WOODED MARSH	Red maple wooded sedge/fern marsh
MIXED SWAMP: MID/HIGH	Red spruce-red maple/winterberry swamp
WETLAND SHRUB THICKET	Speckled alder-arrow wood-bluejoint shrub swamp
BARREN: TALUS SLOPE	Chestnut oak-black birch-virginia creeper wooded talus slopes
BARREN: TALUS SLOPE	Hemlock-black birch/mt maple wooded talus & scree
BARREN: PITCH PINE	Pitch pine-Scarlet oak low-mid elevation ridgetop
WETLAND SHRUB THICKET	Chokeberry-winterberry-mt holly shrub swamp
FEN: CALCAREOUS	Prairie sedge-tussock sedge fen
BARREN: CALCAREOUS	Side oats gramma calcareous glade opening
BARREN: PITCH PINE	Little bluestem-poverty grass low to mid elevation outcrop Opening
FEN: CALCAREOUS	sedge-cottongrass peatland fen
BOG	Sphagnum-cottongrass bog

BARREN: CALCAREOUS	White cedar/Red cedar wooded calcareous outcrops (southern type?)
BARREN: CALCAREOUS	Chinquapin oak-red cedar calcareous woodland (southern type?)
DECIDUOUS FOREST: XERIC: CHESTNUT OAK	Red oak-Chestnut oak acid mid-high elevation, rocky slopes
CONIFER FOREST: MID/LOW: MIDSLOPE: HEMLOCK-PINE	White pine-blueberry forest of low elevation slopes and hills
CONIFER FOREST: MID/LOW ELEVATION:VALLEY& FLATS	Red cedar successional forest
SHRUB SUMMIT:HIGH ELEVATION	Mountain laurel-great laurel summits
DECIDUOUS FOREST: HIGH ELEVATION	Yellow birch-skunk current/polypody forest
WETLAND SHRUB THICKET	Highbush blueberry shrub swamp
BOG	leatherleaf bog (reconstituted)
DECIDUOUS FOREST: FLOODPLAIN	Red maple-green ash forested swamp
DECIDUOUS FOREST:SUCCESSIONAL	Successional Paper birch forest
MIXED FOREST: MID/LOW ELEVATION	Pine-Northern hardwood forest
CONIFER FOREST:HIGH ELEVATION:STEEP SLOPES	Red Spruce-great laurel forest
MIXED FOREST: HIGH ELEVATION: SPRUCE	Red spruce-yellow birch-black cherry forest
CONIFER FOREST:HIGH ELEVATION:STEEP SLOPES	Red Spruce /Southern mt. Cranberry forest
SUMMIT: HEATH BALD	Blueberry-black chokeberry heath
BARREN: PINE	Table mt pine-pitch pine mid elevation xeric ridgetop
DECIDUOUS FOREST: XERIC: CHESTNUT OAK	Red oak-Chestnut oak acidic mid-high elevation, rocky summits
BARREN: CALCAREOUS	Chinquapin oak-ragwort calcareous woodland
FEN: MAFIC	Canada burnet mafic fen
CLIFF: GREENSTONE	Ninebark high elevation greenstone cliffbase
CONIFER FOREST: MID/LOW ELEVATION:CALCAREOUS SOILS	Northern white cedar forest
CONIFER SWAMP: MID/HIGH	Red spruce high elevation wooded wetland
SUMMIT: GRASS BALD	Wild oat-three seeded cinquefoil grassy opening

ROCKY SUMMIT: HIGH ELEVATION	Saxifrage-goldenrod rocky summit (acidic type?)
SUMMIT: GRASS BALD	Poverty grass-goldenrod grassy opening
ROCKY SUMMIT: HIGH ELEVATION	Saxifrage-goldenrod rocky summit (mafic type?)
BARREN: SHALE	Red cedar-white ash alkaline shale woodland
CONIFER FOREST: RED PINE	Red pine-poverty grass forest
MIXED FOREST: HIGH ELEVATION: SPRUCE	Red spruce-Mt ash woodlands
BARREN: SHALE	Chestnut oak-virginia pine/hairgrass acidic shale woodland (northern type)
BARREN: SHALE	Virginia pine-red cedar/Pennsylvania sedge shale woodlands (northern type)
BARREN: SHALE	Virginia pine/ragwort/houstonia shale woodland (southern type)
BARREN: SHALE	Chestnut oak-virginia pine/ragwort acidic shale woodland (southern type)
BARREN: PINE	Virginia pine -Chestnut oak low to mid elevation sandstone pavement barren
BARREN: SHALE	Pennsylvania sedge-poverty grass acidic shale opening
BARREN: CALCAREOUS	White cedar/Red cedar wooded calcareous outcrops (northern type)
DECIDUOUS FOREST:SUCCESSIONAL	Successional Tree-of-heaven forest
SEEP: CALCAREOUS	Skunk cabbage-marsh marigold seep
CONIFER FOREST: MID/LOW: MIDSLOPE: HEMLOCK-PINE	White pine-eastern hemlock/great laurel dry forest:southern Type
DECIDUOUS FOREST: MID/LOW: XERIC: OAK-HICKORY	Oak-hickory-Fraxinus dry-mesic, rich forests
MIXED FOREST: MID/LOW ELEVATION	Virginia pine - Oak xeric forest
MIXED FOREST: MID/LOW ELEVATION	Successional virginia pine-mixed oak forest
DECIDUOUS FOREST:SUCCESSIONAL	Red maple upland forest
DECIDUOUS FOREST: MESIC: LOW SLOPES & COVES	Beech-maple-tuliptree forest (matrix,large patch)
DECIDUOUS FOREST: MESIC: LOW SLOPES & COVES	Sugar maple-white ash-basswood cove forest (matrix/large

	patch)
MIXED FOREST: MID/LOW ELEVATION	White pine-oak-beech dry forest(large patch to matrix)
DECIDUOUS FOREST:MID/LOW XERIC:SLOPES	Black oak-white oak-hickory/dogwood forest:(matrix) dry, dry-mesic, low elevation
DECIDUOUS FOREST: MESIC: LOW SLOPES & COVES	Oak-maple-beech-tulip tree mesic forests (matrix)
DECIDUOUS FOREST: MID/LOW: XERIC: OAK-HICKORY	White oak-red oak-hickory/dogwood forests: (matrix) gentle to moderate slopes, valleys
DECIDUOUS FOREST: XERIC: CHESTNUT OAK	Chestnut oak-scarlet oak/ericad forest: (matrix) xeric, S & SW facing slopes
DECIDUOUS FOREST: XERIC: CHESTNUT OAK	Chestnut oak-black oak/ericad forest: (matrix) xeric, S & SW facing slopes
DECIDUOUS FOREST: MID/LOW: MIXED MESOPHYTIC	Mixed mesophytic forest (matrix)
DECIDUOUS FOREST: XERIC: CHESTNUT OAK	Chestnut oak-red oak/ericad forest: (matrix) N slopes
MIXED FOREST: MID/LOW ELEVATION	Hemlock/white pine-red oak-mixed hardwood forest
RIVERSHORE	Tapegrass submersed rivershore
SHRUB SUMMIT:HIGH ELEVATION	Bramble-goldenrod thicket
WET MEADOW	Canada bluejoint-Reed canarygrass meadow
WET MEADOW	Canada bluejoint meadow
RIVERSHORE:SHALLOWS	Tape-grass shallow shore
MARSH	Bulrush marsh
MARSH	Three way sedge basin marsh
WET MEADOW	Carex stricta wet meadow
RIVERSHORE:SHALLOWS	Water-willow shallow shore
AQUATIC: LAKE/POND	Pickerelweed-arrow arrum emergent vegetation
AQUATIC: LAKE/POND	Water lily emergent vegetation
AQUATIC: LAKE/POND	Spatterdock emergent vegetation
RIVERSHORE:SHALLOWS	River-weed shallow shore
OUTCROP	Lichen dominated shaded outcrops
OUTCROP	Lichen dominated sandstone cliff, outcrops and talus

RIVERSHORE: SHRUB THICKET	Black willow thickets
MARSH	Canada bluejoint-tussock sedge meadow
CONIFER FOREST: MID/LOW ELEVATION:RIDGES & SLOPES	Virginia pine/heath forest of extremely steep, dry, SW facing ridges
WETLAND SHRUB THICKET	Buttonbush shrub swamp
MARSH	Cattail marsh
SEEP: ACIDIC	Golden saxifrage forested seep
MIXED FOREST: MID/LOW ELEVATION	White pine-oak-tulip tree dry forest
RIVERSHORE: GRASSLAND	Reed canarygrass-bluejoint floodplain meadow
DECIDUOUS FOREST:SUCCESSIONAL	Successional tuliptree forest
DECIDUOUS FOREST:SUCCESSIONAL	Successional black Locust disturbed forests
DECIDUOUS FOREST:SUCCESSIONAL	Successional pin cherry forest
DECIDUOUS FOREST:SUCCESSIONAL	Successional aspen/grey birch forest
RIVERSHORE:SPARSE	Goldenrod-aster scoured rivershore
CLIFF: ACIDIC	Spleenwort acidic cliff
SEEP: ACIDIC	Nasturium-water speedwell-spring cress forested spring
WET MEADOW	Goldenrod-aster-dewberry wet field
RIVERSHORE:SPARSE	Loosestrife-dogbane scoured rivershore
DECIDUOUS FOREST: FLOODPLAIN	Silver maple-American elm-cottonwood floodplain forest
CONIFER FOREST: MID/LOW ELEVATION:VALLEY& FLATS	Virginia pine successional forest
DECIDUOUS FOREST: FLOODPLAIN	Sycamore-river birch-jewelweed floodplain forest

Table S9. LANDFIRE Undeveloped Biophysical System Representation within the Resilient and Connected Network (RCN)

Group	**LANDFIRE Biophysical System Name** (Source: LC16_BPS_200)	Percent of Total Acres of this Biophysical System within RCN	RCN Acres of this Biophysical System
Conifer	Atlantic Coastal Plain Fall-line Sandhills Longleaf Pine Woodland	38.7	1,228,323
Conifer	Atlantic Coastal Plain Upland Longleaf Pine Woodland	12.3	1,573,218
Conifer	Boreal Aspen-Birch Forest	42.6	38,092
Conifer	Boreal Jack Pine-Black Spruce Forest	83.4	625,202
Conifer	Boreal Jack Pine-Black Spruce Forest-Pine Barrens	35.2	204,614
Conifer	Boreal Jack Pine-Black Spruce Forest-Spruce-Fir	50.6	30,011
Conifer	Boreal White Spruce-Fir-Hardwood Forest-Aspen-Birch	68.5	2,126,139
Conifer	Boreal White Spruce-Fir-Hardwood Forest-Coastal	56.3	645,569
Conifer	Boreal White Spruce-Fir-Hardwood Forest-Inland	55.2	2,275,190
Conifer	California Coastal Closed-Cone Conifer Forest and Woodland	72.3	4,423
Conifer	California Coastal Redwood Forest	71.0	1,880,111
Conifer	California Montane Jeffrey Pine(-Ponderosa Pine) Woodland	62.6	2,185,929
Conifer	Central and Southern Appalachian Spruce-Fir Forest	98.2	566,151
Conifer	Colorado Plateau Pinyon-Juniper Shrubland	68.2	83,535
Conifer	Colorado Plateau Pinyon-Juniper Woodland	71.7	8,939,106
Conifer	Columbia Plateau Western Juniper Woodland and Savanna	54.8	1,042,483
Conifer	East Cascades Mesic Montane Mixed-Conifer Forest and Woodland	59.4	980,405
Conifer	East Cascades Oak-Ponderosa Pine Forest and Woodland	31.2	215,096
Conifer	East Gulf Coastal Plain Interior Upland Longleaf Pine Woodland	19.7	3,221,323
Conifer	East-Central Texas Plains Southern Pine Forest and Woodland	57.5	200,692
Conifer	Edwards Plateau Limestone Shrubland	48.0	2,363,031
Conifer	Florida Longleaf Pine Sandhill	23.9	531,925
Conifer	Great Basin Pinyon-Juniper Woodland	92.8	5,765,487
Conifer	Great Lakes Alvar	33.4	5,407
Conifer	Inter-Mountain Basins Juniper Savanna	55.4	286,852

Conifer	Inter-Mountain Basins Subalpine Limber-Bristlecone Pine Woodland	96.3	154,394
Conifer	Klamath-Siskiyou Lower Montane Serpentine Mixed Conifer Woodland	81.7	306,549
Conifer	Klamath-Siskiyou Upper Montane Serpentine Mixed Conifer Woodland	97.9	214,725
Conifer	Klamath-Siskiyou Xeromorphic Serpentine Savanna and Chaparral	31.6	13,527
Conifer	Laurentian Pine-Oak Barrens-Jack Pine	45.3	1,165,562
Conifer	Madrean Encinal	83.5	2,214,614
Conifer	Madrean Juniper Savanna	79.0	301,260
Conifer	Madrean Lower Montane Pine-Oak Forest and Woodland	83.8	1,376,314
Conifer	Madrean Pinyon-Juniper Woodland	95.4	2,783,124
Conifer	Madrean Upper Montane Conifer-Oak Forest and Woodland	97.5	19,294
Conifer	Mediterranean California Dry-Mesic Mixed Conifer Forest and Woodland	57.7	3,697,909
Conifer	Mediterranean California Lower Montane Black Oak-Conifer Forest and Woodland	45.1	609,215
Conifer	Mediterranean California Mesic Mixed Conifer Forest and Woodland	59.9	4,258,195
Conifer	Mediterranean California Mixed Evergreen Forest	56.7	1,744
Conifer	Mediterranean California Mixed Evergreen Forest-Coastal	65.7	697,456
Conifer	Mediterranean California Mixed Evergreen Forest-Interior	79.3	1,726,126
Conifer	Mediterranean California Red Fir Forest	77.8	763,757
Conifer	Mediterranean California Red Fir Forest-Cascades	75.4	796,646
Conifer	Mediterranean California Red Fir Forest-Southern Sierra	81.0	1,101,844
Conifer	Mediterranean California Subalpine Woodland	89.6	527,605
Conifer	Middle Rocky Mountain Montane Douglas-fir Forest and Woodland	72.1	4,770,998
Conifer	Middle Rocky Mountain Montane Douglas-fir Forest and Woodland-Fire-maintained Savanna	50.4	75,351
Conifer	North Pacific Dry Douglas-fir(-Madrone) Forest and Woodland	47.8	279,824
Conifer	North Pacific Dry-Mesic Silver Fir-Western Hemlock-Douglas-fir Forest	89.6	1,462,422
Conifer	North Pacific Hypermaritime Sitka Spruce Forest	39.5	938,729
Conifer	North Pacific Hypermaritime Western Red-cedar-Western Hemlock Forest	58.0	801,065
Conifer	North Pacific Maritime Dry-Mesic Douglas-fir-Western Hemlock Forest	63.1	3,309,694

Conifer	North Pacific Maritime Mesic Subalpine Parkland	98.5	200,803
Conifer	North Pacific Maritime Mesic-Wet Douglas-fir-Western Hemlock Forest	47.0	3,989,336
Conifer	North Pacific Mesic Western Hemlock-Silver Fir Forest	84.2	2,318,112
Conifer	North Pacific Mountain Hemlock Forest-Wet	95.9	823,047
Conifer	North Pacific Mountain Hemlock Forest-Xeric	94.2	808,445
Conifer	North Pacific Wooded Volcanic Flowage	44.5	2,560
Conifer	Northeastern Interior Pine Barrens	36.2	58,896
Conifer	Northern Atlantic Coastal Plain Pitch Pine Barrens	37.3	318,870
Conifer	Northern California Mesic Subalpine Woodland	85.3	41,307
Conifer	Northern Rocky Mountain Conifer Swamp	70.2	317,427
Conifer	Northern Rocky Mountain Dry-Mesic Montane Mixed Conifer Forest	44.0	3,079,625
Conifer	Northern Rocky Mountain Dry-Mesic Montane Mixed Conifer Forest-Grand Fir	60.6	2,144,081
Conifer	Northern Rocky Mountain Dry-Mesic Montane Mixed Conifer Forest-Larch	53.0	1,120,368
Conifer	Northern Rocky Mountain Dry-Mesic Montane Mixed Conifer Forest-Lodgepole Pine	90.4	168,200
Conifer	Northern Rocky Mountain Dry-Mesic Montane Mixed Conifer Forest-Ponderosa Pine-Douglas-fir	55.9	4,824,623
Conifer	Northern Rocky Mountain Foothill Conifer Wooded Steppe	41.7	62,382
Conifer	Northern Rocky Mountain Mesic Montane Mixed Conifer Forest	52.4	2,610,239
Conifer	Northern Rocky Mountain Mesic Montane Mixed Conifer Forest-Cedar Groves	86.3	19,601
Conifer	Northern Rocky Mountain Ponderosa Pine Woodland and Savanna	59.2	1,504,791
Conifer	Northern Rocky Mountain Ponderosa Pine Woodland and Savanna-Mesic	25.4	931,480
Conifer	Northern Rocky Mountain Ponderosa Pine Woodland and Savanna-Xeric	33.7	421,374
Conifer	Northern Rocky Mountain Subalpine Woodland and Parkland	91.0	5,939,004
Conifer	Northwestern Great Plains Highland White Spruce Woodland	67.2	60,960
Conifer	Northwestern Great Plains-Black Hills Ponderosa Pine Woodland and Savanna	54.3	286,741
Conifer	Northwestern Great Plains-Black Hills Ponderosa Pine Woodland and Savanna-Low Elevation Woodland	72.0	1,003,622

Conifer	Northwestern Great Plains-Black Hills Ponderosa Pine Woodland and Savanna-Savanna	78.1	1,481,544
Conifer	Ozark-Ouachita Shortleaf Pine-Oak Forest and Woodland	63.5	2,932,358
Conifer	Rocky Mountain Foothill Limber Pine-Juniper Woodland	78.8	418,625
Conifer	Rocky Mountain Lodgepole Pine Forest	64.0	1,338,785
Conifer	Rocky Mountain Poor-Site Lodgepole Pine Forest	32.9	462,587
Conifer	Rocky Mountain Subalpine Dry-Mesic Spruce-Fir Forest and Woodland	71.7	11,319,558
Conifer	Rocky Mountain Subalpine Mesic-Wet Spruce-Fir Forest and Woodland	81.2	7,741,768
Conifer	Rocky Mountain Subalpine-Montane Limber-Bristlecone Pine Woodland	84.2	167,985
Conifer	Sierra Nevada Subalpine Lodgepole Pine Forest and Woodland	96.4	36,071
Conifer	Sierra Nevada Subalpine Lodgepole Pine Forest and Woodland-Dry	85.0	62,188
Conifer	Sierra Nevada Subalpine Lodgepole Pine Forest and Woodland-Wet	82.0	131,857
Conifer	Sierran-Intermontane Desert Western White Pine-White Fir Woodland	45.2	65,162
Conifer	South Florida Pine Flatwoods	35.1	356,164
Conifer	South Florida Pine Rockland	75.8	29,556
Conifer	Southeastern Interior Longleaf Pine Woodland	47.3	274,193
Conifer	Southern Appalachian Low-Elevation Pine Forest	59.6	749,505
Conifer	Southern Appalachian Montane Pine Forest and Woodland	89.4	561,690
Conifer	Southern Rocky Mountain Dry-Mesic Montane Mixed Conifer Forest and Woodland	78.7	3,079,862
Conifer	Southern Rocky Mountain Juniper Woodland and Savanna	73.0	2,914,673
Conifer	Southern Rocky Mountain Mesic Montane Mixed Conifer Forest and Woodland	80.0	1,824,264
Conifer	Southern Rocky Mountain Pinyon-Juniper Woodland	85.6	2,284,258
Conifer	Southern Rocky Mountain Ponderosa Pine Savanna	46.6	1,590,799
Conifer	Southern Rocky Mountain Ponderosa Pine Savanna-North	78.6	4,783
Conifer	Southern Rocky Mountain Ponderosa Pine Savanna-South	93.7	181
Conifer	Southern Rocky Mountain Ponderosa Pine Woodland	64.4	6,523,991
Conifer	Southern Rocky Mountain Ponderosa Pine Woodland-North	67.6	82,181
Conifer	Southern Rocky Mountain Ponderosa Pine Woodland-South	72.7	231,669

Conifer	West Gulf Coastal Plain Upland Longleaf Pine Forest and Woodland	27.5	688,822
Grassland	Apacherian-Chihuahuan Semi-Desert Grassland and Steppe	51.4	9,767,088
Grassland	Arkansas Valley Prairie and Woodland-Prairie	19.5	128,707
Grassland	Arkansas Valley Prairie and Woodland-Woodland	30.4	175,589
Grassland	California Central Valley and Southern Coastal Grassland	60.6	592,392
Grassland	California Mesic Serpentine Grassland	72.8	6,948
Grassland	California Northern Coastal Grassland	77.4	50,577
Grassland	Central and Upper Texas Coast Dune and Coastal Grassland	77.4	85,092
Grassland	Central Interior Highlands Calcareous Glade and Barrens	30.8	369,264
Grassland	Central Mixedgrass Prairie	26.0	17,563,998
Grassland	Central Tallgrass Prairie	11.4	6,098,114
Grassland	Chihuahuan Gypsophilous Grassland and Steppe	10.9	141,485
Grassland	Chihuahuan Loamy Plains Desert Grassland	16.0	1,148,413
Grassland	Chihuahuan Sandy Plains Semi-Desert Grassland	13.8	360,017
Grassland	Chihuahuan-Sonoran Desert Bottomland and Swale Grassland	18.3	229,932
Grassland	Chihuahuan-Sonoran Desert Bottomland and Swale Grassland-Alkali Sacaton	34.0	356,270
Grassland	Chihuahuan-Sonoran Desert Bottomland and Swale Grassland-Tobosa Grassland	32.0	355,217
Grassland	Columbia Basin Foothill and Canyon Dry Grassland	44.8	120,366
Grassland	Columbia Basin Palouse Prairie	2.0	2,162
Grassland	Columbia Plateau Steppe and Grassland	44.1	6,458,592
Grassland	East Gulf Coastal Plain Dune and Coastal Grassland	33.0	1,339
Grassland	East Gulf Coastal Plain Jackson Plain Prairie and Barrens	16.3	1,813
Grassland	Eastern Great Plains Wet Meadow-Prairie-Marsh	7.3	34,491
Grassland	Eastern Highland Rim Prairie and Barrens	9.9	6,606
Grassland	Florida Dry Prairie	41.9	341,945
Grassland	Great Plains Prairie Pothole	31.2	266,218
Grassland	Inter-Mountain Basins Semi-Desert Grassland	23.8	2,036,355
Grassland	Lower Mississippi Alluvial Plain Grand Prairie	7.3	50,958
Grassland	Mediterranean California Alpine Dry Tundra	91.2	8,353
Grassland	Mediterranean California Subalpine Meadow	91.3	11,839
Grassland	North Pacific Alpine and Subalpine Dry Grassland	99.4	172,840

Grassland	North Pacific Montane Grassland	71.3	78,641
Grassland	North-Central Interior Sand and Gravel Tallgrass Prairie	20.2	591,506
Grassland	Northern Atlantic Coastal Plain Dune and Swale	40.9	71,313
Grassland	Northern Rocky Mountain Lower Montane-Foothill-Valley Grassland	51.6	2,666,903
Grassland	Northern Rocky Mountain Subalpine-Upper Montane Grassland	66.4	218,530
Grassland	Northern Tallgrass Prairie	7.3	2,426,816
Grassland	Northwestern Great Plains Mixedgrass Prairie	34.0	37,905,797
Grassland	Pennyroyal Karst Plain Prairie and Barrens	13.9	3,317
Grassland	Rocky Mountain Alpine Fell-Field	74.1	269,409
Grassland	Rocky Mountain Alpine Turf	60.6	217,653
Grassland	Rocky Mountain Subalpine-Montane Mesic Meadow	59.8	439,311
Grassland	South Texas Sand Sheet Grassland	49.0	494,355
Grassland	Southeastern Great Plains Tallgrass Prairie	30.1	7,877,263
Grassland	Southern Appalachian Grass and Shrub Bald	96.7	2,086
Grassland	Southern Atlantic Coastal Plain Dune and Maritime Grassland	26.7	972
Grassland	Southern Blackland Tallgrass Prairie	37.3	3,211,258
Grassland	Southern Coastal Plain Blackland Prairie and Woodland	21.9	228,456
Grassland	Southern Rocky Mountain Montane-Subalpine Grassland	54.6	641,446
Grassland	Tamaulipan Clay Grassland	28.8	6,274
Grassland	Tamaulipan Savanna Grassland	29.3	1,312,312
Grassland	Texas-Louisiana Coastal Prairie	25.5	1,967,560
Grassland	Texas-Louisiana Saline Coastal Prairie	43.3	110,348
Grassland	West Gulf Coastal Plain Northern Calcareous Prairie	27.8	67,430
Grassland	West Gulf Coastal Plain Southern Calcareous Prairie	31.6	62,666
Grassland	Western Great Plains Foothill and Piedmont Grassland	64.8	1,931,288
Grassland	Western Great Plains Sand Prairie	53.7	14,479,007
Grassland	Western Great Plains Shortgrass Prairie	35.7	21,238,336
Grassland	Western Great Plains Tallgrass Prairie	31.3	901,021
Grassland	Western Highland Rim Prairie and Barrens	45.2	4,590
Hardwood	Alabama Ketona Glade and Woodland	73.4	35,383
Hardwood	Allegheny-Cumberland Dry Oak Forest and Woodland	45.4	7,298,593
Hardwood	Appalachian (Hemlock-)Northern Hardwood Forest	46.9	4,620,285
Hardwood	Appalachian Shale Barrens	91.7	2,336

Hardwood	Atlantic Coastal Plain Dry and Dry-Mesic Oak Forest	19.0	348,198
Hardwood	Atlantic Coastal Plain Mesic Hardwood Forest	19.2	1,090,924
Hardwood	Bluegrass Savanna and Woodland	6.0	15,731
Hardwood	California Central Valley Mixed Oak Savanna	36.2	447,605
Hardwood	California Coastal Live Oak Woodland and Savanna	73.6	560,639
Hardwood	California Lower Montane Blue Oak-Foothill Pine Woodland and Savanna	70.4	5,286,512
Hardwood	Central and South Texas Coastal Fringe Forest and Woodland	50.3	588,071
Hardwood	Central and Southern Appalachian Montane Oak Forest	89.5	1,548,061
Hardwood	Central and Southern California Mixed Evergreen Woodland	87.7	2,442,199
Hardwood	Central Atlantic Coastal Plain Maritime Forest	26.8	49,281
Hardwood	Central Interior Highlands Dry Acidic Glade and Barrens	25.4	50,986
Hardwood	Crosstimbers Oak Forest and Woodland	36.0	4,622,601
Hardwood	East Gulf Coastal Plain Limestone Forest	14.3	15,873
Hardwood	East Gulf Coastal Plain Northern Dry Upland Hardwood Forest	25.8	1,144,179
Hardwood	East Gulf Coastal Plain Northern Loess Bluff Forest	32.2	539,103
Hardwood	East Gulf Coastal Plain Northern Loess Plain Oak-Hickory Upland	4.9	39,766
Hardwood	East Gulf Coastal Plain Northern Mesic Hardwood Slope Forest	21.1	120,158
Hardwood	East Gulf Coastal Plain Southern Loess Bluff Forest	58.8	369,174
Hardwood	East-Central Texas Plains Post Oak Savanna and Woodland	55.2	3,742,153
Hardwood	Eastern Boreal Floodplain	62.2	496,175
Hardwood	Eastern Great Plains Tallgrass Aspen Parkland	53.7	272,510
Hardwood	Edwards Plateau Dry-Mesic Slope Forest and Woodland	71.9	1,655,506
Hardwood	Edwards Plateau Limestone Savanna and Woodland	41.9	4,044,927
Hardwood	Edwards Plateau Mesic Canyon	84.7	80,776
Hardwood	Laurentian-Acadian Northern Hardwoods Forest	55.0	11,911,887
Hardwood	Laurentian-Acadian Northern Hardwoods Forest-Hemlock	36.2	6,547,380
Hardwood	Laurentian-Acadian Northern Hardwoods Forest-Northern Sugar Maple-Basswood	66.0	64,254
Hardwood	Llano Uplift Acidic Forest-Woodland-Glade	47.7	290,388
Hardwood	Lower Mississippi River Dune Woodland and Forest	8.5	23,737
Hardwood	Lower Mississippi River Flatwoods	9.7	278,693

Hardwood	Mediterranean California Mixed Oak Woodland	77.6	905,634
Hardwood	Mississippi River Alluvial Plain Dry-Mesic Loess Slope Forest	55.5	63,375
Hardwood	North Pacific Broadleaf Landslide Forest and Shrubland	39.5	25,323
Hardwood	North Pacific Oak Woodland	34.5	363,341
Hardwood	North-Central Interior Beech-Maple Forest	3.8	672,518
Hardwood	North-Central Interior Dry Oak Forest and Woodland	29.7	1,471,048
Hardwood	North-Central Interior Dry-Mesic Oak Forest and Woodland	14.5	4,352,371
Hardwood	North-Central Interior Maple-Basswood Forest	21.7	2,386,415
Hardwood	North-Central Interior Oak Savanna	25.8	3,468,052
Hardwood	North-Central Interior Wet Flatwoods	15.0	205,249
Hardwood	North-Central Oak Barrens	26.2	674,295
Hardwood	Northeastern Interior Dry-Mesic Oak Forest	30.6	7,328,784
Hardwood	Northern Atlantic Coastal Plain Hardwood Forest	19.2	476,589
Hardwood	Northwestern Great Plains Aspen Forest and Parkland	61.7	1,606
Hardwood	Ouachita Montane Oak Forest	97.4	7,528
Hardwood	Ozark-Ouachita Dry Oak Woodland	51.5	7,244,134
Hardwood	Ozark-Ouachita Dry-Mesic Oak Forest	51.9	5,281,486
Hardwood	Ozark-Ouachita Mesic Hardwood Forest	74.1	1,156,733
Hardwood	Piedmont Hardpan Woodland and Forest	3.0	709
Hardwood	Rocky Mountain Aspen Forest and Woodland	67.8	4,555,146
Hardwood	Rocky Mountain Bigtooth Maple Ravine Woodland	62.5	360,181
Hardwood	South Florida Hardwood Hammock	26.2	13,593
Hardwood	South-Central Interior Mesophytic Forest	43.2	8,151,332
Hardwood	South-Central Interior/Upper Coastal Plain Flatwoods	12.6	13,212
Hardwood	South-Central Interior/Upper Coastal Plain Wet Flatwoods	15.6	12,487
Hardwood	Southeast Florida Coastal Strand and Maritime Hammock	3.2	409
Hardwood	Southern and Central Appalachian Cove Forest	77.5	1,978,310
Hardwood	Southern Appalachian Northern Hardwood Forest	94.5	240,703
Hardwood	Southern Appalachian Oak Forest	75.6	3,899,533
Hardwood	Southern California Oak Woodland and Savanna	80.4	487,777
Hardwood	Southern Coastal Plain Dry Upland Hardwood Forest	30.7	2,169,777
Hardwood	Southern Coastal Plain Mesic Slope Forest	26.0	1,806,846
Hardwood	Southern Crowley`s Ridge Mesic Loess Slope Forest	55.2	50,761

Hardwood	Southern Interior Low Plateau Dry-Mesic Oak Forest	30.0	4,834,913
Hardwood	Southern Piedmont Mesic Forest	19.3	1,440,744
Hardwood	Southern Ridge and Valley/Cumberland Dry Calcareous Forest	32.2	1,092,294
Hardwood	Southwest Florida Coastal Strand and Maritime Hammock	33.8	12,026
Hardwood	West Gulf Coastal Plain Chenier and Upper Texas Coastal Fringe Forest and Woodland	59.5	47,981
Hardwood	West Gulf Coastal Plain Mesic Hardwood Forest	40.8	760,647
Hardwood	West Gulf Coastal Plain Nonriverine Wet Hardwood Flatwoods	24.1	249,748
Hardwood	Western Great Plains Dry Bur Oak Forest and Woodland	61.5	1,111,161
Hardwood	Willamette Valley Upland Prairie and Savanna	11.3	115,020
Hardwood-Conifer	Acadian Low-Elevation Spruce-Fir-Hardwood Forest	64.9	6,230,676
Hardwood-Conifer	Acadian-Appalachian Montane Spruce-Fir Forest	95.9	2,552,408
Hardwood-Conifer	Central Appalachian Dry Oak-Pine Forest	41.1	4,876,315
Hardwood-Conifer	Central Appalachian Pine-Oak Rocky Woodland	57.0	431,179
Hardwood-Conifer	East Gulf Coastal Plain Interior Shortleaf Pine-Oak Forest	26.9	1,033,903
Hardwood-Conifer	East Gulf Coastal Plain Maritime Forest	50.3	51,591
Hardwood-Conifer	Eastern Serpentine Woodland	30.1	391
Hardwood-Conifer	Inter-Mountain Basins Aspen-Mixed Conifer Forest and Woodland	70.0	3,678,661
Hardwood-Conifer	Inter-Mountain Basins Aspen-Mixed Conifer Forest and Woodland-High Elevation	59.6	803,166
Hardwood-Conifer	Inter-Mountain Basins Aspen-Mixed Conifer Forest and Woodland-Low Elevation	62.5	418,211
Hardwood-Conifer	Laurentian Pine-Oak Barrens	29.6	419,034
Hardwood-Conifer	Laurentian-Acadian Northern Pine(-Oak) Forest	39.4	1,990,014
Hardwood-Conifer	Laurentian-Acadian Northern Pine(-Oak) Forest-Pine Dominated	51.1	1,879,926
Hardwood-Conifer	Laurentian-Acadian Pine-Hemlock-Hardwood Forest	45.9	4,100,560
Hardwood-Conifer	Mississippi Delta Maritime Forest	0.0	0
Hardwood-Conifer	Northern Atlantic Coastal Plain Maritime Forest	48.4	89,456
Hardwood-Conifer	Northern Crowley`s Ridge Sand Forest	17.0	62,493
Hardwood-Conifer	Ozark-Ouachita Shortleaf Pine-Bluestem Woodland	52.4	1,877,155
Hardwood-Conifer	Paleozoic Plateau Bluff and Talus	48.0	683,122
Hardwood-Conifer	Southern Atlantic Coastal Plain Maritime Forest	32.0	32,351
Hardwood-Conifer	Southern Piedmont Dry Oak(-Pine) Forest	13.8	3,023,759

Hardwood-Conifer	West Gulf Coastal Plain Pine-Hardwood Flatwoods	26.5	598,150
Hardwood-Conifer	West Gulf Coastal Plain Pine-Hardwood Forest	20.7	2,217,445
Hardwood-Conifer	West Gulf Coastal Plain Sandhill Oak and Shortleaf Pine Forest and Woodland	23.5	143,106
Riparian	Atlantic Coastal Plain Clay-Based Carolina Bay Wetland	46.6	34,921
Riparian	Atlantic Coastal Plain Peatland Pocosin and Canebrake	71.1	711,551
Riparian	Atlantic Coastal Plain Streamhead Seepage Swamp-Pocosin-Baygall	49.6	262,542
Riparian	Boreal Acidic Peatland Systems	59.9	5,091,262
Riparian	California Central Valley Riparian Woodland and Shrubland	17.5	630,618
Riparian	California Montane Riparian Systems	71.7	1,706,968
Riparian	Caribbean Coastal Wetland Systems	0.5	1,604
Riparian	Caribbean Swamp Systems	57.2	339,810
Riparian	Central Atlantic Coastal Plain Nonriverine Swamp and Wet Hardwood Forest	46.9	373,611
Riparian	Central Atlantic Coastal Plain Wet Longleaf Pine Savanna and Flatwoods	29.0	718,038
Riparian	Central Florida Pine Flatwoods	39.1	1,495,265
Riparian	Central Interior and Appalachian Floodplain Systems	26.6	4,720,883
Riparian	Central Interior and Appalachian Floodplain Systems-Large Floodplains	39.7	647,951
Riparian	Central Interior and Appalachian Riparian Systems	32.3	4,753,266
Riparian	Central Interior and Appalachian Shrub-Herbaceous Wetland Systems	37.2	388,911
Riparian	Central Interior and Appalachian Swamp Systems	21.4	1,211,843
Riparian	East Gulf Coastal Plain Near-Coast Pine Flatwoods	42.9	1,489,063
Riparian	East Gulf Coastal Plain Southern Loblolly-Hardwood Flatwoods	22.8	183,956
Riparian	Eastern Great Plains Floodplain Systems	21.8	802,671
Riparian	Edwards Plateau Riparian	55.1	200,321
Riparian	Floridian Highlands Freshwater Marsh	61.7	412,308
Riparian	Great Lakes Coastal Marsh Systems	42.8	83,542
Riparian	Great Lakes Wet-Mesic Lakeplain Prairie	18.3	29,721
Riparian	Great Lakes Wooded Dune and Swale	57.7	52,315
Riparian	Gulf and Atlantic Coastal Plain Floodplain Systems	43.7	14,965,970
Riparian	Gulf and Atlantic Coastal Plain Small Stream Riparian Systems	32.7	4,659,247
Riparian	Gulf and Atlantic Coastal Plain Swamp Systems	39.7	5,583,896

Riparian	Gulf and Atlantic Coastal Plain Tidal Marsh Systems	26.8	1,178,810
Riparian	Inter-Mountain Basins Montane Riparian Systems	33.3	1,869,748
Riparian	Laurentian-Acadian Alkaline Conifer-Hardwood Swamp	56.9	3,369,494
Riparian	Laurentian-Acadian Floodplain Systems	51.1	1,202,856
Riparian	Laurentian-Acadian Shrub-Herbaceous Wetland Systems	56.3	972,237
Riparian	Laurentian-Acadian Swamp Systems	64.1	1,210,351
Riparian	North American Warm Desert Riparian Systems	41.5	1,173,832
Riparian	North American Warm Desert Riparian Systems-Stringers	62.4	1,300,714
Riparian	North Pacific Lowland Riparian Forest and Shrubland	45.6	811,429
Riparian	North Pacific Montane Riparian Woodland and Shrubland-Dry	61.8	121,258
Riparian	North Pacific Montane Riparian Woodland and Shrubland-Wet	53.8	298,145
Riparian	North Pacific Swamp Systems	62.2	66,162
Riparian	Pacific Coastal Marsh Systems	80.1	74,791
Riparian	Rocky Mountain Montane Riparian Systems	51.3	6,798,709
Riparian	Rocky Mountain Subalpine/Upper Montane Riparian Systems	57.6	1,759,291
Riparian	South Florida Cypress Dome	53.3	45,381
Riparian	South Florida Everglades Sawgrass Marsh	65.2	1,579,742
Riparian	Southern Atlantic Coastal Plain Wet Pine Savanna and Flatwoods	31.4	848,238
Riparian	Southern Coastal Plain Nonriverine Cypress Dome	57.8	640,645
Riparian	Southern Coastal Plain Seepage Swamp and Baygall	40.3	1,012,255
Riparian	Tamaulipan Floodplain	17.9	524
Riparian	Tamaulipan Riparian Systems	30.1	640,169
Riparian	West Gulf Coastal Plain Wet Longleaf Pine Savanna and Flatwoods	25.7	959,397
Riparian	Western Great Plains Depressional Wetland Systems	48.1	1,632,194
Riparian	Western Great Plains Depressional Wetland Systems-Playa	23.2	77,014
Riparian	Western Great Plains Depressional Wetland Systems-Saline	25.0	5,035
Riparian	Western Great Plains Floodplain Systems	43.1	8,199,644
Savanna	Central Appalachian Alkaline Glade and Woodland	31.7	38,987
Savanna	East Gulf Coastal Plain Savanna and Wet Prairie	29.1	93,096
Savanna	Nashville Basin Limestone Glade and Woodland	5.7	12,201
Savanna	South Florida Dwarf Cypress Savanna	94.9	44,741

Shrubland	Acadian-Appalachian Alpine Tundra	76.5	34,042
Shrubland	Acadian-Appalachian Subalpine Woodland and Heath-Krummholz	99.1	76,446
Shrubland	Apacherian-Chihuahuan Mesquite Upland Scrub	29.4	324,642
Shrubland	California Maritime Chaparral	86.2	11,087
Shrubland	California Mesic Chaparral	83.9	821,139
Shrubland	California Montane Woodland and Chaparral	78.6	382,148
Shrubland	California Xeric Serpentine Chaparral	80.8	29,090
Shrubland	Chihuahuan Creosotebush Desert Scrub	51.8	1,201,508
Shrubland	Chihuahuan Mixed Desert and Thorn Scrub	73.9	1,359,122
Shrubland	Chihuahuan Mixed Desert and Thorn Scrub-Shrubland	22.8	414,821
Shrubland	Chihuahuan Mixed Desert and Thorn Scrub-Steppe	39.9	1,318,273
Shrubland	Chihuahuan Mixed Salt Desert Scrub	28.6	211,471
Shrubland	Chihuahuan Stabilized Coppice Dune and Sand Flat Scrub	16.7	84,648
Shrubland	Chihuahuan Succulent Desert Scrub	61.6	4,240,978
Shrubland	Colorado Plateau Blackbrush-Mormon-tea Shrubland	60.7	1,155,684
Shrubland	Colorado Plateau Mixed Low Sagebrush Shrubland	29.4	673,377
Shrubland	Columbia Plateau Low Sagebrush Steppe	54.7	491,197
Shrubland	Columbia Plateau Scabland Shrubland	66.0	771,656
Shrubland	Florida Peninsula Inland Scrub	25.8	71,260
Shrubland	Great Basin Semi-Desert Chaparral	89.5	498,358
Shrubland	Great Basin Xeric Mixed Sagebrush Shrubland	78.7	17,712,326
Shrubland	Inter-Mountain Basins Big Sagebrush Shrubland	40.9	703,355
Shrubland	Inter-Mountain Basins Big Sagebrush Shrubland-Basin Big Sagebrush	31.8	1,046,716
Shrubland	Inter-Mountain Basins Big Sagebrush Shrubland-Semi-Desert	41.5	2,382,264
Shrubland	Inter-Mountain Basins Big Sagebrush Shrubland-Upland	49.5	10,119,418
Shrubland	Inter-Mountain Basins Big Sagebrush Shrubland-Wyoming Big Sagebrush	45.9	9,315,297
Shrubland	Inter-Mountain Basins Big Sagebrush Steppe	47.1	18,303,902
Shrubland	Inter-Mountain Basins Curl-leaf Mountain Mahogany Woodland and Shrubland	85.6	1,560,441
Shrubland	Inter-Mountain Basins Greasewood Flat	21.7	2,258,255
Shrubland	Inter-Mountain Basins Mat Saltbush Shrubland	33.2	1,041,161
Shrubland	Inter-Mountain Basins Mixed Salt Desert Scrub	31.6	9,844,431

Shrubland	Inter-Mountain Basins Montane Sagebrush Steppe	61.0	17,002,937
Shrubland	Inter-Mountain Basins Montane Sagebrush Steppe-Low Sagebrush	42.5	238,819
Shrubland	Inter-Mountain Basins Montane Sagebrush Steppe-Mountain Big Sagebrush	48.6	1,657,143
Shrubland	Inter-Mountain Basins Semi-Desert Shrub-Steppe	25.6	1,216,948
Shrubland	Madrean Oriental Chaparral	92.7	50,247
Shrubland	Mediterranean California Alpine Fell-Field	90.2	10,687
Shrubland	Mediterranean California Mesic Serpentine Woodland and Chaparral	80.0	54,516
Shrubland	Mogollon Chaparral	80.5	3,614,125
Shrubland	Mojave Mid-Elevation Mixed Desert Scrub	64.5	8,022,257
Shrubland	North Pacific Avalanche Chute Shrubland	98.9	35,812
Shrubland	North Pacific Dry and Mesic Alpine Dwarf-Shrubland or Fell-field or Meadow	99.8	278,451
Shrubland	North Pacific Montane Shrubland	92.4	46,242
Shrubland	Northern and Central California Dry-Mesic Chaparral	86.1	3,727,081
Shrubland	Northern California Coastal Scrub	69.0	115,158
Shrubland	Northern Rocky Mountain Montane-Foothill Deciduous Shrubland	36.0	395,273
Shrubland	Northwestern Great Plains Shrubland	61.1	2,234,868
Shrubland	Rocky Mountain Alpine Dwarf-Shrubland	85.7	129,936
Shrubland	Rocky Mountain Gambel Oak-Mixed Montane Shrubland	53.3	1,919,247
Shrubland	Rocky Mountain Gambel Oak-Mixed Montane Shrubland - Continuous	66.0	492,919
Shrubland	Rocky Mountain Gambel Oak-Mixed Montane Shrubland-Patchy	52.7	195,804
Shrubland	Rocky Mountain Lower Montane-Foothill Shrubland	57.6	898,251
Shrubland	Rocky Mountain Lower Montane-Foothill Shrubland-No True Mountain Mahogany	58.3	16,096
Shrubland	Rocky Mountain Lower Montane-Foothill Shrubland-True Mountain Mahogany	84.3	4,404
Shrubland	Sierra Nevada Alpine Dwarf-Shrubland	90.0	23,332
Shrubland	Sonora-Mojave Creosotebush-White Bursage Desert Scrub	42.7	10,996,103
Shrubland	Sonora-Mojave Mixed Salt Desert Scrub	19.0	902,691
Shrubland	Sonora-Mojave Semi-Desert Chaparral	75.5	1,450,860
Shrubland	Sonoran Granite Outcrop Desert Scrub	67.2	3,847,365
Shrubland	Sonoran Mid-Elevation Desert Scrub	82.1	1,994,489

Shrubland	Sonoran Paloverde-Mixed Cacti Desert Scrub	52.5	1,277,863
Shrubland	South Texas Lomas	64.6	25,024
Shrubland	Southern California Coastal Scrub	78.7	1,483,133
Shrubland	Southern California Dry-Mesic Chaparral	77.4	1,720,603
Shrubland	Southern Colorado Plateau Sand Shrubland	21.5	450,015
Shrubland	Tamaulipan Calcareous Thornscrub	34.5	578,491
Shrubland	Tamaulipan Mixed Deciduous Thornscrub	23.0	1,984,730
Shrubland	Western Great Plains Mesquite Woodland and Shrubland	46.5	582,034
Shrubland	Western Great Plains Sandhill Steppe	42.7	5,890,706
Shrubland	Western Great Plains Wooded Draw and Ravine	52.1	1,125,235
Shrubland	Wyoming Basins Dwarf Sagebrush Shrubland and Steppe	29.9	31,752
Sparse	Gulf and Atlantic Coastal Plain Sparsely Vegetated Systems	36.8	283
Sparse	Inter-Mountain Basins Sparsely Vegetated Systems	39.9	4,164,460
Sparse	Mediterranean California Sparsely Vegetated Systems	60.6	3,376
Sparse	North American Warm Desert Sparsely Vegetated Systems	54.4	644,271
Sparse	North Pacific Sparsely Vegetated Systems	96.6	10,743
Sparse	Northwestern Great Plains Canyon	29.7	1,245
Sparse	Rocky Mountain Alpine/Montane Sparsely Vegetated Systems	80.9	998,270
Sparse	Western Great Plains Sparsely Vegetated Systems	54.2	221,461
Barren-Rock/Sand/Clay	Barren-Rock/Sand/Clay	45.9	9,804,032

Table S10. List of scientists and conservationists who contributed time or participated on a steering committee to create the Resilient and Connected Network for the Continental U.S.

Organization and position may have changed since the time of participation.

Name	Organization
Aaron Jones	The Nature Conservancy: OR
Abby Weinberg	Open Space Institute
Abigail Derby Lewis	Chicago Wilderness
Aimee Roberson	Rio Grande Joint Venture
Aimee Weldon	U.S. Fish and Wildlife Service
Albert George	South Carolina Aquarium
Alex Jospe	The Nature Conservancy: ECS
Alex Wyss	The Nature Conservancy: TN
Alison Bowden	The Nature Conservancy: MA
Allison Vogt	The Nature Conservancy: MD
Amie Treuer-Kuehn	Texas Parks and Wildlife Department
Amy Cimarolli	The Nature Conservancy: WV
Amy Crouch	The Nature Conservancy: IA
Amy Keister	U.S. Fish and Wildlife Service
Amy Pocewicz	US Fish and Wildlife Service: LCC
Analie Barnett	The Nature Conservancy: ECS
Andrea Brandon	The Nature Conservancy: MN
Andrew Cutko	The Nature Conservancy: ME
Andrew Milliken	U.S. Fish and Wildlife Service: LCC
Andy Finton	The Nature Conservancy: MA
Angela Watland	The Nature Conservancy: VA
Anne Deaton	NC Department of Natural Resources
Anne Gage	World Wildlife Fund, Northern Great Plains Program
Anthony Gonzon	Delaware NHP/DNR:
Arlen Lancaster	The Nature Conservancy: WY
Arlene Olivero	The Nature Conservancy: ECS
Art Bettis	University of Iowa
Ashby Worley	The Nature Conservancy: SC
August Froehlich	The Nature Conservancy: OH
Barbara Charry	The Nature Conservancy: MO
Barbara Vickery	The Nature Conservancy: ME
Bartholomew Wilson	U.S. Fish and Wildlife Service: LCC
Becca Benner	The Nature Conservancy: NY
Betsy Neely	The Nature Conservancy: CO
Bill Romme	Colorado State University
Blaik Keppler	South Carolina Department of Natural Resources
Blair Tirpak	US Geological Survey
Bob Allen	The Nature Conservancy: NJ
Bob Unnasch	The Nature Conservancy: ID
Brad Compton	University of Massachusetts
Brad McCrae	The Nature Conservancy: NA
Brad Potter	U.S. Fish and Wildlife Service: LCC
Brian Boutin	The Nature Conservancy: NC
Brian Cohen	The Nature Conservancy: CA
Brian Martin	The Nature Conservancy: MT
Brian Obermeyer	The Nature Conservancy: KS
Brian Tavernia.	The Nature Conservancy: CO
Bruce Carlson	Minnesota Department of Natural Resources
Bryan Piazza	The Nature Conservancy: LA

Carl Ferraro	Alabama Department of Conservation
Carol McCartney	Wisconsin Geological Survey
Carolyn Currin	National Oceanic and Atmospheric Administration
Carrie Schloss	The Nature Conservancy: CA
Cary Hamel	Nature Conservancy of Canada
Cat Burns	The Nature Conservancy: NC
Charles Ferree	The Nature Conservancy: ECS
Charlotte Reemts	The Nature Conservancy: TX
Chester Jackson	Georgia Southern University
Chris Bruce	The Nature Conservancy: VA
Chris Helzer	The Nature Conservancy: NE
Chris Hise	The Nature Conservancy: OK
Chris Pague	The Nature Conservancy: CO
Christi Lambert	The Nature Conservancy: GA
Christine Shepard	The Nature Conservancy: SE
Christopher McGuire	The Nature Conservancy: MA
Colette Degarady	The Nature Conservancy: SE
Corey Anderson	Florida Fish & Wildlife Commission
Corrina Riginos	The Nature Conservancy: WY
Courtney Larson	The Nature Conservancy: WY
Craig Allen	USGS Nebraska Cooperative Fish and Wildlife Unit
Craig Harding	Nature Conservancy of Canada
Dan Coker	The Nature Conservancy: ME
Dan Krause	Nature Conservancy of Canada
Daniel Brown	Great Lakes Integrated Science Assessment
Darlene Finch	National Oceanic and Atmospheric Administration
Dave Theobald	Conservation Science Partners
David Harlan	The Nature Conservancy: LA
David Kozak	Connecticut DEEP:
David Patrick	The Nature Conservancy: NH
David Pyke	US Geological Survey
David Ray	Open Space Institute
Dick Cameron	The Nature Conservancy: CA
Dirk Bryant	The Nature Conservancy: NY
Donnelle Keech	The Nature Conservancy: MD
Doria Gordon	The Nature Conservancy: FL
Doug Ladd	The Nature Conservancy: MO
Doug Pearsall	The Nature Conservancy: MI
Doug Samson	The Nature Conservancy: MD
Doug Zollner	The Nature Conservancy: AR
Ed Sherwood	Tampa Bay National Estuary Program
Elizabeth Kalies	The Nature Conservancy: NC
Elizabeth Kitchens	The Nature Conservancy: WI
Elizabeth Thompson	Vermont Land Trust
Eric Krueger	The Nature Conservancy: SC
Erik Martin	The Nature Conservancy: ECS
Eric Odell	Colorado Division of Wildlife
Eric Sorenson	Natural Heritage Program: Vermont
Eric Sparks	Mississippi State University
Esther Rubin	Arizona Game and Fish
Gabriel De Jong	The Nature Conservancy: LA
Gary Knight	Natural Heritage Program: Florida
Geoffrey Smith	The Nature Conservancy: ME
Greg Suba	California Native Plant Society
Gwen White	U.S. Fish and Wildlife Service
Gwynn Crichton	The Nature Conservancy: VA

Hannah Texler	Minnesota Department of Natural Resources
Heather Reading	The Nature Conservancy: AZ
Holly Copeland	The Nature Conservancy: WY
Jacqueline Ferrato	The Nature Conservancy: TX
James Broska	U.S. Fish and Wildlife Service: LCC
James Cole	The Nature Conservancy: MO
Jamie Carter	National Oceanic and Atmospheric Administration
Jason Bird	CH2M & Jacobs
Jay Odell	The Nature Conservancy: NA
Jay Osenkowski	Rhode Island DEM:
Jay Pruett	The Nature Conservancy: OK
Jeff Sole	The Nature Conservancy: KY
Jeff Walk	The Nature Conservancy: IL
Jeffry Evans	The Nature Conservancy: NA
Jennifer Roberts	Mississippi State University
Jenny Gleeson	Great Lakes Integrated Science Assessment
Jeremy Bell	The Nature Conservancy: ME
Jerry Lorenz	Florida Audubon
Jessica Dyson	The Nature Conservancy: MA
Jill Gambill	University of Georgia
Jim Bergan	The Nature Conservancy: LA
Jim Platt	The Nature Conservancy: MN
Jimi Gragg	Utah Division of Wildlife
Jodie LaPoint	The Nature Conservancy: NC
Joe Fargione	The Nature Conservancy: NA
Joel Tuhy	The Nature Conservancy: UT
John Herron	The Nature Conservancy: TX
John Karges	The Nature Conservancy: TX
John Prince	The Nature Conservancy: NC
John Sanderson	The Nature Conservancy: CO
John Shuey	The Nature Conservancy: ID
John Tull	U.S. Fish and Wildlife Service
Jon Ambrose	Georgia DNR
Jon Oetting	Florida Natural Areas
Jorge Brenner	The Nature Conservancy: TX
Joseph Wisby	The Nature Conservancy: TN
Josh Royte	The Nature Conservancy: ME
Judy Dunscomb	The Nature Conservancy: VA
Judy Haner	The Nature Conservancy: AL
Justin Schlawin	Natural Heritage Program: Maine
Kathy Chase	USGS Wyoming-Montana Water Science Center
Kathy Freeman	The Nature Conservancy: FL
Katie Frerker	USFS Superior National Forest
Katie Gillies	The Nature Conservancy: OK
Kaylan Carrlson	Ducks Unlimited
Keith Laakkonen	Florida Department of Envir. Protection
Ken Popper	The Nature Conservancy: OR
Kevin Ruddock	The Nature Conservancy: RI
Kim Hall	The Nature Conservancy: MI
Kim Lutz	The Nature Conservancy: MA
Kirsten Puryear	Natural Heritage Program: Maine
Kirstin Dow	University of South Carolina
Kristin Szabo	Natural Heritage Program: Nevada
Kristina Serbesoff-King	The Nature Conservancy: FL
Kyle Steele	Natural Resources Conservation Service
Laura Geselbracht	The Nature Conservancy: FL

Laura Mitchell	U.S. Fish and Wildlife Service
Lauren Alleman	The Nature Conservancy: LA
Lily Swansboro-Becker	Florida Fish & Wildlife Commission
Lily Verdone	The Nature Conservancy: TX
Linda Pearsall	Natural Heritage Program: North Carolina
Lisa Morris	The Nature Conservancy: KY
Lisa Williams	The Nature Conservancy: TX
Lise Hanners	The Nature Conservancy: NA
Liz Fly	The Nature Conservancy: SC
Louis Iverson	USFS Northern Research Station
Louis Provencher	The Nature Conservancy: NV
Louise Gratton	Nature Conservancy of Canada
Malcolm Hodges	The Nature Conservancy: GA
Marc Carullo	Massachusetts CZM
Marci Bortman	The Nature Conservancy: NY
Marcos Robles	The Nature Conservancy: AZ
Margaret Fields	The Nature Conservancy: NC
Maria Whitehead	The Nature Conservancy: SC
Marissa Ahlering	The Nature Conservancy: MN
Mark Anderson	The Nature Conservancy: ECS
Mark Bryer	The Nature Conservancy: MD
Mark White	The Nature Conservancy: MN
Mark Woodrey	Mississippi State University
Mark Zankel	The Nature Conservancy: NH
Marta Ribera	The Nature Conservancy: ECS
Mary Conley	The Nature Conservancy: SE
Mary Kate Brown	The Nature Conservancy: AL
Matt Dallman	The Nature Conservancy: WI
Matt Hurteau	University of New Mexico
Matt Kauffman	University of Wyoming
Matthew Braun	Nature Conservancy of Canada
Megan de Graaf	Community Forest International
Megan N. Sutton	The Nature Conservancy: NC
Megan Tyrrell	U.S. Fish and Wildlife Service: LCC
Melissa Clark	The Nature Conservancy: ECS
Meredith Cornett	The Nature Conservancy: MN
Metthea Yepsen	The Nature Conservancy: NJ
Mhairi McFarlane	Nature Conservancy of Canada
Michael Notaro	University of Wisconsin-Madison
Michael Pressman	The Nature Conservancy: MN
Michael Schindel	The Nature Conservancy: OR
Michael Schwartz	Conservation Fund
Michael Shirley	Florida Department of Environmental Protection
Michele DePhilip	The Nature Conservancy: PA
Michelle Canick	The Nature Conservancy: MD
Moses Katkowski	The Nature Conservancy: NJ
Nancy Fishbein	The Nature Conservancy: CO
Nancy Sferra	The Nature Conservancy: ME
Naomi Fraga	Rancho Santa Ana Botanic Garden
Nate Herold	National Oceanic and Atmospheric Administration
Neil Jordan	Open Space Institute
Nels Johnson	The Nature Conservancy: NA
Nick Jensen	California Native Plant Society
Nick Miller	The Nature Conservancy: WI
Nicole Athearn	US National Park Service, Great Rivers Cooperative
Nicole Carlozo	Massachusetts CZM

NOAA:	National Oceanic and Atmospheric Administration
Norman Bliss	U.S. Geological Society, EROS
Paige Lewis	The Nature Conservancy: CO
Pat Comer	NatureServe
Patricia Dalyander	US Geological Survey
Patricia Doerr	The Nature Conservancy: NJ
Patricia Harveson	Borderlands Research Institute at Sol Ross State University
Paul Beier	Northern Arizona University
Paul Freeman	The Nature Conservancy: AL
Peter Steckler	The Nature Conservancy: MH
Phil Gerla	University of North Dakota
Philip B. Huffman	The Nature Conservancy: VT
Ralph Grundel	U.S. Geological Survey
Rebecca Benner	The Nature Conservancy: NY
Rebecca Shirer	The Nature Conservancy: NY
Regina Lyons	Environmental Protection Agency
Reide Corbett	East Carolina University
Rhode Island DEM:	Rhode Island DEM:
Rich Kostecke	The Nature Conservancy: TX
Rick Gorsira	CH2M & Jacobs
Rick Nelson	U.S. Fish and Wildlife Service: LCC
Rick Schneider	Natural Heritage Program: Nebraska
Rick Studenmund	The Nature Conservancy: NC
Rodney Bartgis	The Nature Conservancy: WV
Ron Sutherland	Wildlands Institute
Ronnie Drever	The Nature Conservancy: Canada
Rose Paul	The Nature Conservancy: VT
Rua Mordecai	U.S. Fish and Wildlife Service: LCC
Ruth Thornton	The Nature Conservancy: WV
Ryan Haugo	The Nature Conservancy: OR
Ryan O’Connor	Wisconsin Natural Heritage Inventory
Sally McGee	The Nature Conservancy: NE
Sally Palmer	The Nature Conservancy: TN
Sara Gottlieb	The Nature Conservancy: GA
Sarah Hagen	The Nature Conservancy: IL
Sarah Watson	South Carolina Sea Grant
Scott Bearer.	The Nature Conservancy: PA
Scott Lemmons	The Nature Conservancy: MI
Scott Schwenk	U.S. Fish and Wildlife Service: LCC
Sharon Richardson	South Carolina Audubon
Shonene Scott	The Nature Conservancy: OR
Sonia Hall	The Nature Conservancy: WA
Sonia Najera	The Nature Conservancy: TX
Sophie Parker	The Nature Conservancy: CA
Stacey Clark	Natural Resources Conservation Service
Stephen Handler	Northern Institute of Applied Climate Science
Steve Ashby	Mississippi State University
Steve Bassett	The Nature Conservancy: NM
Steve Buttrick	The Nature Conservancy: OR
Steve Fuller	U.S. Fish and Wildlife Service
Steve Herrington	The Nature Conservancy: MO
Steve Traxler	U.S. Fish and Wildlife Service
Steve Walker	The Nature Conservancy: NH
Susan Adamowicz	U.S. Fish and Wildlife Service
Susan Rees	Alabama US Army Corps of Engineers
Susanne Hickey	The Nature Conservancy: IA

Suzanne Perdeaux	Ontario Centre for Climate Impacts
Tamara Gagnolet	The Nature Conservancy: PA
Teresa Chapman	The Nature Conservancy: CO
Terri Schulz	The Nature Conservancy: CO
Thomas Minney	The Nature Conservancy: WV
Thomas Morhman	The Nature Conservancy: MS
Tiffany Troxler	Florida International University
Tim Jones	U.S. Fish and Wildlife Service
Todd Hopkins	U.S. Fish and Wildlife Service
Tracie Sempier	Mississippi/Alabama Sea Grant
Wade Harrison	The Nature Conservancy: GA
Walker Golder	National Audubon Society
Wendy Ledbetter.	The Nature Conservancy: TX
Whitney Gray	Florida Department of Environmental Protection
Zach Ferdana	The Nature Conservancy: NA

Supplementary References

M. G. Anderson, M. Clark, A. O. Sheldon, Estimating Climate Resilience for Conservation across Geophysical Settings. *Conservation Biology* **28**, 959–970 (2014).

Fels, J.E. Landscape Position and Classified Landtype Mapping for the Statewide DRASTIC Mapping Project. Raleigh, NC: North Carolina State University. Technical report to the North Carolina Department of Environment, Health, and Natural Resources, Division of Environmental Management. (1995)

McCune B and Keon D. Equations for potential annual direct incident radiation and heat load. Journal of Vegetation Science 13(4):603–606 (2002).

Moore, ID., P.E. Gessler, G.A. Nielsen, and G.A. Petersen. Terrain attributes: estimation methods and scale effects. In Modeling Change in Environmental Systems, edited by A.J. Jakeman M.B. Beck and M. McAleer Wiley , London, pp. 189-214 (1993).

Wilson J. P. and Gallant J. C. Digital Terrain Analysis in Wilson J P and Gallant J d (eds) Terrain analysis; principles and Applications. New York: John Wiley and Sons 1-27 (2000).
